# Ion-Pair Selective Conformational Rearrangement of Sulfonamide Calix[6]arene-Based Pseudorotaxanes

**DOI:** 10.1021/acs.orglett.0c01191

**Published:** 2020-04-14

**Authors:** Gianpiero Cera, Margherita Bazzoni, Arturo Arduini, Andrea Secchi

**Affiliations:** Dipartimento di Scienze Chimiche, della Vita e della Sostenibilità Ambientale, Università di Parma, Parco Area delle Scienze 17/A, I-43124 Parma, Italy

## Abstract

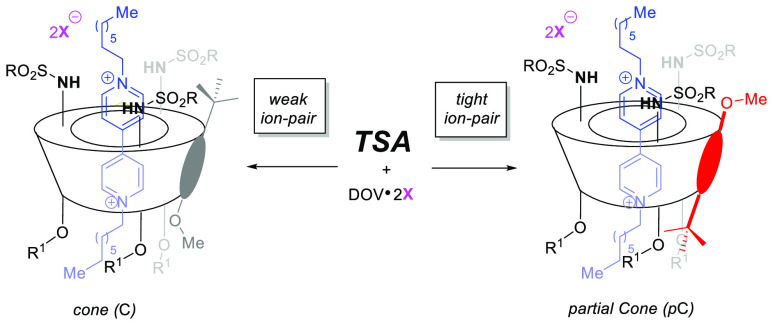

We describe the synthesis of a new class of trisulfonamide calix[6]arene-based wheels that can bind dialkylviologen salts, in apolar media. The threading process occurs through a selective ion-pair recognition, established by the sulfonamide groups with the counterions of the bipyridinium salts, that dictates a conformational rearrangement of the corresponding pseudorotaxanes.

For many decades, organic chemistry has tackled the challenge of building synthetic stimulus-responsive devices and prototypes of receptors and working devices for the development of nanotechnologies.^1^ In this context, triphenylureido calix[6]arene (TPU) has already proven to be a versatile scaffold for the formation of inclusion complexes with 4,4′-bipyridinium (viologen) salts, leading to oriented (pseudo)rotaxanes, in apolar media.^2^ Under these conditions, this process is largely dictated by hydrogen bonding interactions established by weak acidic phenylureido groups (p*K*_a_ ≈ 18), which can efficiently separate the ion pair of bipyridinium salts, promoting threading of cationic axles inside the π-rich aromatic wheel.^3^ Threading of the axles through the upper rim of the macrocycles is usually independent of the features of the ion pair, leading TPU (pseudo)rotaxanes to always adopt a cone conformation. With the aim of understanding in more detail the role of the binding sites in the conformational control of calix[6]arene-based pseudorotaxanes, our group recently reported on a new 1,4-diphenylureido calix[6]arene (DPU) receptor that adopts a predominant 1,2,3-alternate conformation in low-polarity solvents.^4^ Quite unexpectedly, although this latter wheel can form viologen-based pseudorotaxanes, an unselective 1:1 mixture, with cone and 1,2,3-alternate geometries, was obtained.^5^ Because one of the most important goals of supramolecular chemistry is the control of a stimulus-induced event, which triggers a conformational preference of synthetic receptors,^6^ we speculated that a calix[6]arene bearing more acidic NH-sulfonamide groups (p*K*_a_ ≈ 14) would be able to respond to the complexation with a selective conformational rearrangement. We thus started our investigation with the synthesis of a small library of trisulfonamide calix[6]arenes (TSA) **1** from the known trioctyloxy trinitro derivative TN.^7^ Reduction of the nitro groups with hydrazine in the presence of catalytic amounts of Pd/C led to the formation of the triamino derivative that was not isolated but further reacted in the presence of an excess of the corresponding arylsulfonyl chloride and triethylamine, in CH_2_Cl_2_ at room temperature. Hence, the targeted compounds **1a–d** were isolated in moderate to good yields after flash column chromatography (Figure 1).

**Figure 1:**
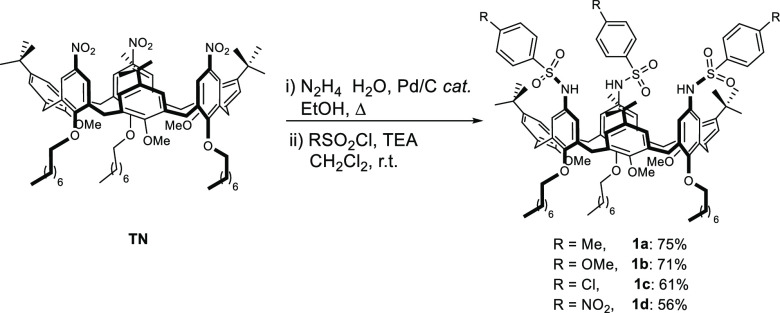
Synthesis of sulfonamido calix[6]arenes (TSA) **1**.

Their conformation in solution was investigated by one- and two-dimensional NMR analysis. In particular, in apolar solvents such as CDCl_3_ and CD_2_Cl_2_, **1a–d** adopt, on the NMR time scale, a pseudocone conformation with their methoxy groups pointing toward the center of the cavity as reflected by their unusual upfield shift (approximately 2.5 ppm) of the ^1^H NMR resonance. This geometry is further confirmed by the AX system of two doublets at δ 4.4 and 3.4 (^2^*J* = 15.5 Hz), related to the bridging axial and equatorial methylene groups of the macrocycle. The ability of TSA **1a** to form a pseudorotaxane was subsequently verified by equilibrating a solution of the former in CDCl_3_ with a dioctylviologen ditosylate DOV·2OTs at 298 K. A deep yellow solution was afforded, and the mixture analyzed by NMR spectroscopy. The main features of the ^1^H NMR spectrum are the extensive upfield shift of the aromatic C–H and N–CH_2_ bonds signals of the guest that are suggestive of the inclusion in the cavity of a calixarene macrocycle. Interestingly, as a consequence of the threading, the protons of the methoxy groups suffered a substantial downfield shift (1.5–2 ppm), giving rise to a new pattern of signals in a 2:1 ratio (*£* + *$*), which are suggestive of a new conformation, different from the typical cone displayed by the well-established TPU-based (pseudo)rotaxanes.^2e,2f^ A new pattern for the methylene bridging protons was also found (Figure 2).

**Figure 2:**
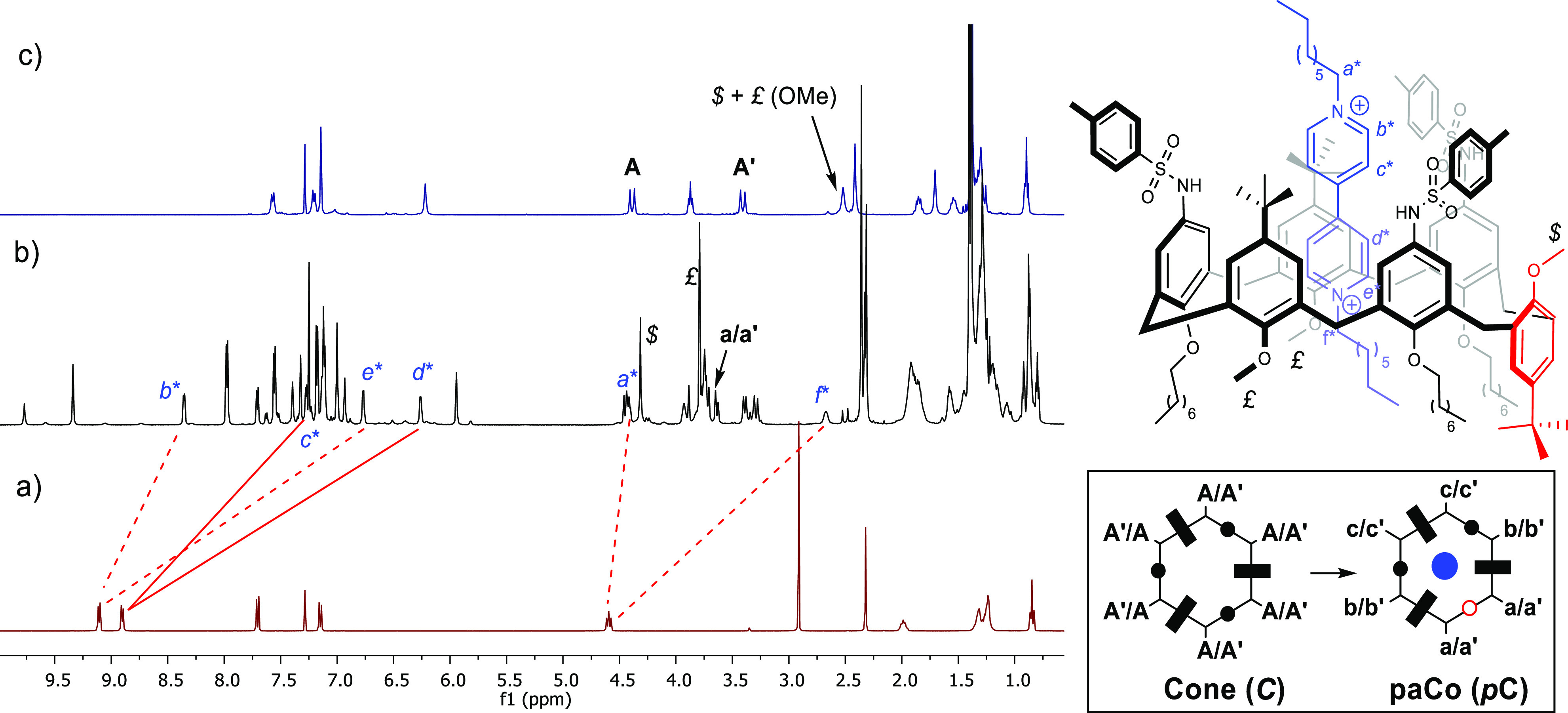
^1^H NMR spectra (400 MHz, 298 K) of (a) DOV·2OTs in CD_3_CN, (b) pseudorotaxane P[**1a**(*p*C)⊃DOV]2OTs in CDCl_3_, and (c) calixarene **1a** in CDCl_3_. At the bottom right are schematic representations of **1a** and P[**1a**(*p*C)⊃DOV]2OTs. The colors of the ovals and rectangles indicate the relative position of the phenolic substituent with respect to the plane defined by the bridging methylene groups (hexagon) (black, upward; white, downward). The rectangle identifies the phenolic ring substituted with the octyloxy chains, while the circle those with the methoxy groups.

Indeed, the two doublets (ax+eq, **A**+**A′**) of the methylene bridge of **1a** split in six doublets, with geminal coupling, three for the axial and three for the equatorial, in a 1:1 ratio. HSQC-NMR analysis further revealed a substantial shift of the ^13^C NMR resonances to δ 35.7 for the **a/a′** couple typical of an *anti* orientation and indicative of a single inversion point (see Figure S1). A complete characterization by NMR analysis^8^ was consistent with the formation of pseudorotaxane P[**1a**(*p*C)⊃DOV]2OTs in which the host molecule adopts a partial cone (*pa*Co or *p*C) conformation, with the inversion associated with a ring bearing a methoxy group (see the inset of Figure 2 and Figure S7). Spectrophotometric titrations were conducted to measure the stability of this pseudorotaxane complex. The calculated apparent stability constant log *K*_1:1_ of ∼4 (Table 1 and Supporting Information for the collection of spectra) was found to be 2 orders of magnitude lower than that of the corresponding pseudorotaxane obtained employing a TPU analogue.^3^ This finding can be explained considering that, as opposed to those of TPU, the sulfonamide H-bond donor groups in TSA cannot form bifurcated hydrogen bonds with the anions. Moreover, the stronger EWG nature of the sulfonamide moiety somehow depletes the π-rich aromatic cavity of the wheel, thus reducing the cation−π and charge transfer interactions usually operating between the host and the bipyridinium dication.^9^ Intrigued by this result, we subsequently exploited the ability of **1a** to form pseudorotaxanes in the presence of viologen base axles with different counterions. Using DOV·2I as the salt, a different situation was observed. Analysis of ^1^H NMR spectra (see Figures S9 and S10) highlighted two different conformations existing on the NMR time scale; the dominant one was attributed to a pseudorotaxane P[**1a**(C)⊃DOV]2I in which the calixarene host adopts a cone conformation. The stability constant associated with this mixture (Table 1, entry 2) is almost one order of magnitude lower than that associated with P[**1a**(*p*C)⊃DOV]2OTs, which might seem reasonable because the ion pair associated with DOV·2I is weaker and the counterion itself is a poor H-bond acceptor.

**Table 1:**
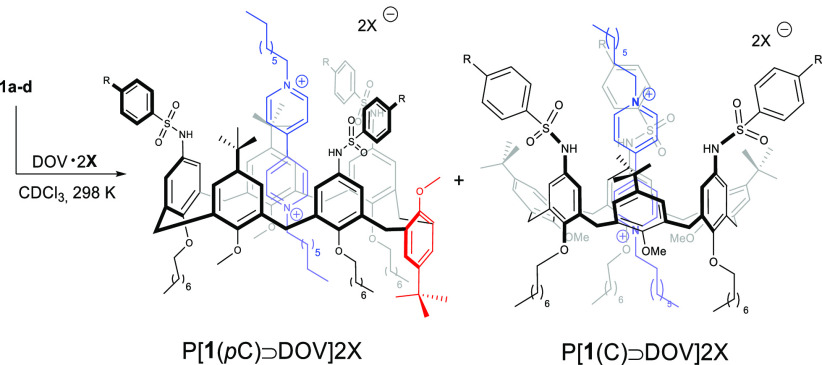
Partial Cone (*p*C):Cone (C) Ratios in the Formation of Pseudorotaxane P[**1a**–**d**⊃DOV]2X (CDCl_3_, 298 K) and Related Apparent Stability Constants Log *K*_1:1_ (CH_2_Cl_2_, 298 K)

entry	**1**	X	*p*C:C	log *K*_1:1_
1	**1a** (R = Me)	TsO	6.3:1	4.4
2	**1a** (R = Me)	I	1:1.7	3.2
3	**1a** (R = Me)	Cl	1:3.0	3.0
4	**1a** (R = Me)	Br	1:2.2	nd
5	**1a** (R = Me)	PF_6_	–	–
6	**1a** (R = Me)	ClO_4_	1:1.6	nd
7	**1b** (R = OMe)	TsO	5.8:1	4.8
8	**1c** (R = Cl)	TsO	3.8:1	4.1
9	**1d** (R = NO_2_)	TsO	2.7:1	3.8

Intuitively, using DOV·2Cl and DOV·2Br as the axles, whose ion pairs are even weaker than the parental iodide, the selectivity was substantially shifted toward pseudorotaxane P[**1a**(C)⊃DOV]2Cl/Br (Table 1, entries 3 and 4). DOV·2PF_6_ was found to be not suitable for complexation. This could be attributed to the weak ability of PF_6_ to engage efficient hydrogen bonding with the sulfonamide group, necessary to overcome the limited π-density of the aromatic core. Interestingly, using perchlorate (ClO_4_) as the counterion whose tetrahedral geometry parallels that of the tosylate, a 1:1.6 *p*C:C distribution was observed (Table 1, entry 6).

Hence, the selectivity (*p*C vs Cone) observed along this series (TsO > ClO_4_ > I > Br > Cl) follows an *anti*-Hofmeister trend.^10^ This could be rationalized with the formation of ligand-separated ion pairs in which an increased number of interactions of the counterions with acidic sulfonamide groups by H-bonding determines not only the stabilization of the pseudorotaxane complex but also the conformational rearrangement of the TSA host.

Subsequently, all of the other TSA derivatives **1b–d** were equilibrated in CDCl_3_ in the presence of DOV·2OTs to determine the effect of the substituent in the *para* position on the formation of pseudorotaxanes and log *K*_1:1_ calculated (Table 1).

In all of the cases studied, the predominant formation of pseudorotaxanes with a partial cone conformation was established by NMR analysis. In particular, the presence of a strong EDG such as the methoxy in TSA **1b** did not affect the outcome of the threading process with the selective formation of P[**1b**(*p*C)⊃DOV]2OTs (>5:1) and a higher apparent constant associated with the former. On the contrary, for TSA bearing EWGs such as chloride and nitro groups, the formation of P[**1c**–**d**(*p*C)⊃DOV]2OTs was associated with a small yet detectable decrease in selectivity. In parallel, the formation of these two new pseudorotaxanes occurred with a diminished log *K*_1:1_.

In analogy to similar studies,^11^ we have constructed a Hammett-type plot correlating log[*K*_P(**1b**–**d**)_/*K*_P(**1a**)_] versus σ_p_. Thus, a linear correlation was observed between the log *K*_1:1_ values and the electronic nature of the substituents at the *para* position of the sulfonamide moiety. This correlation highlights how the enhanced electronic π-density operated by EDGs on the aromatic cavity has a positive effect in increasing the stability of the complexes (Figure 3).

**Figure 3:**
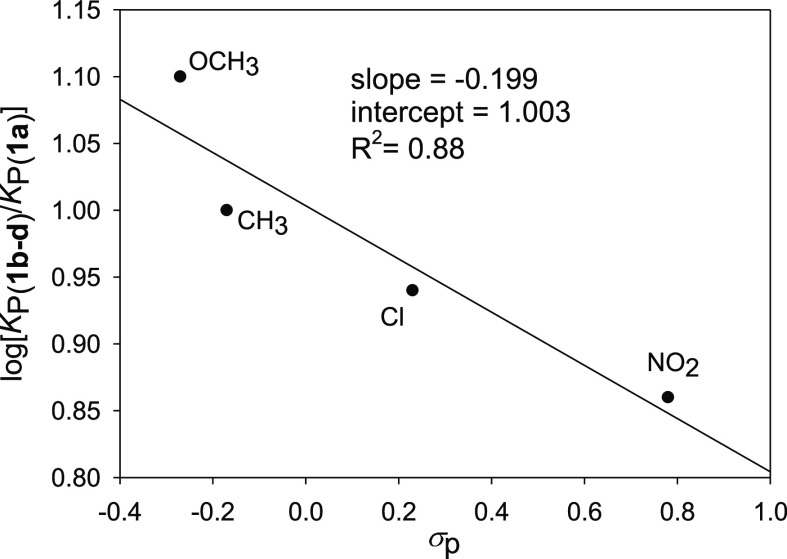
Hammett-type plot.

To gain a more detailed understanding of the host–guest interaction stabilizing these complexes, a preliminary geometry optimization of pseudorotaxanes P[**1a**(C)⊃DOV]2TsO and P[**1a**(*p*C)⊃DOV]2TsO was carried out at the PM6-DH+ level^12^ using the Mopac 2016 program (Figure 4).^13^

**Figure 4:**
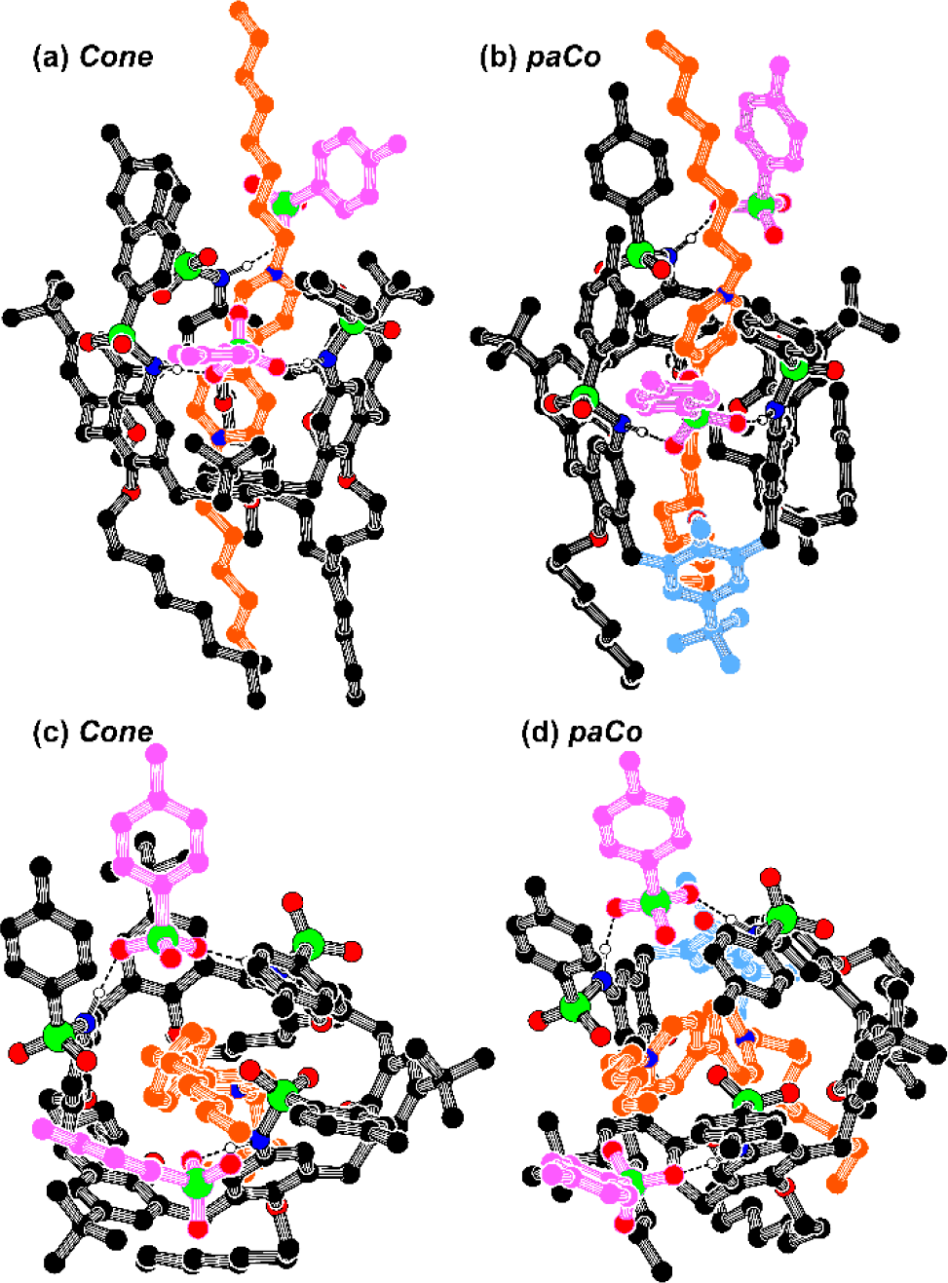
Side and top views of the minimized structures (PM6-DH+) of pseudorotaxanes (a and c) P[**1a**(C)⊃DOV]2TsO (Δ*H*_f_° = −888.88 kcal/mol) and (b and d) P[**1a**(*p*C)⊃DOV]2TsO (Δ*H*_f_° = −912.85 kcal/mol). For the sake of clarity, the backbones of the dioctylviologen thread and the tosylate anions are colored brown and violet, respectively, all hydrogen atoms except those participating in H-bonding have been omitted, and the “flipped” aromatic ring of the paCo conformation in panels b and d are highlighted with a light blue color. Colors: C, black; N, blue; O, red; H, white; S, green; H-bonds, dashed black lines.

The structure of both complexes (see Figure 4) shows that the two tosylate anions are H-bonded with the host tosylamide moieties. However, if one of these anions acts as a bridge between two convergent host NH groups and remains in intimate contact with one of the viologen pyridinium rings, the second one remains fully separated from the other pyridinium ring deeply engulfed in the π-rich aromatic cavity of **1a**. The calculated heat of formation (Δ*H*_f_°) for pseudorotaxane P[**1a**(pC)⊃DOV]2TsO (Figure 4b), which is the more abundant species in solution (see Table 1), was ∼24 kcal/mol lower than that calculated for the corresponding cone isomer (see Figure 4a). This might be rationalized in terms of an appreciable strain release in the whole complex. The weak intermolecular interactions operating in the stabilization of the host/guest adduct were evaluated by NCI-plot analysis (see Figure S16).^14^

In conclusion, we report on the synthesis of a new class of trisulfonamide calix[6]arenes TSA, exploiting their ability to form conformationally controlled pseudorotaxanes by threading viologen-based axles. This unprecedented selectivity is dictated by a subtle interplay of H-bonding interactions that induces the inversion of a phenolic ring. While this rearrangement is driven by thermodynamics, further investigations will be devoted to gaining more insight into the mechanisms behind it. Finally, the synthetic robustness and versatility of this platform of calix[6]arenes will push more efforts toward the synthesis of more complex architectures, i.e., supramolecular cages,^15^ and the eventual application of these systems as Brønsted acid catalysts.^16^

## Supporting Information Available

Synthesis, characterizations, NMR spectroscopy, and spectrophotometric titrations (PDF)
