# Supplementary material for: Ion-Pair Selective Conformational Rearrangement of Sulfonamide Calix[6]arene-Based Pseudorotaxanes
Source: Org Lett. 2020 Apr 14;22(9):3702–5. doi: 10.1021/acs.orglett.0c01191 (PMC7997628; doi:10.1021/acs.orglett.0c01191)
Supplement: Supplementary file 1 — ol0c01191_si_001.pdf [file ol0c01191_si_001.pdf]

# Supporting Information

# Ion-Pair Selective Conformational Rearrangement of Sulphonamide Calix[6]arene-based Pseudorotaxanes

Gianpiero Cera,[a] Margherita Bazzoni,[a] Arturo Arduini,[a] and Andrea Secchi[a]

*[a] Università di Parma, Dipartimento di Scienze Chimiche, della Vita e della Sostenibilità Ambientale, Parco Area delle Scienze 17/A, 43124 Parma, Italy.*

# Table of Contents


| | |
|---|---|
| **General Remarks and Materials** | **S2** |
| **Synthesis of TSA calixarenes 1a-d** | **S3** |
| **Detailed NMR analysis of model pseudorotaxane** P[**1a**⊃DOV]2OTs | **S6** |
| **NMR Spectra** | **S18** |
| **Spectrophotometric Titrations** | **S37** |
| **Computational studies** | **S45** |
| **References** | **S47** |

## General Remarks and Materials

All chemicals those syntheses are not reported hereafter were purchased from commercial sources and used as received. Solvents were dried and stored over molecular sieves previously activated in an oven (450 °C overnight). Column chromatography was performed on silica gel 60 (70-230 mesh). NMR spectra were recorded on a Bruker 400 MHz and JEOL 600 MHz using solvents as internal standards (7.26 ppm for $^{1}$H NMR and 77.00 ppm for $^{13}$C-NMR for $CDCl_3$). The terms m, s, d, t, q and quint represent multiplet, singlet, doublet, triplet, quadruplet and quintuplet respectively, and the term br means a broad signal. Exact masses were recorded on a LTQ ORBITRAP XL Thermo Mass Spectrometer (ESI source). Intermediate **A** was synthesized according to a known procedure.[1] Dioctylviologen salts DOV•2X (OTs,[2] I,[3] Br,[1] Cl,[3] $PF_6$,[4] $ClO_4$[5]) were synthesized following conventional routes. Spectrophotometric titrations (air-equilibrated $CH_2Cl_2$, room temperature) were performed with a JASCO V-750 UV-visible spectrophotomer by adding small aliquots (typically 10 μL) of a concentrated solution of calixarene **1a-d** to a dilute solution of dioctylviologen salts. The titration curves were fitted according to a 1:1 association model by using the SPECFIT/32 software.[6]

## Synthesis of TSA calixarenes 1a-d

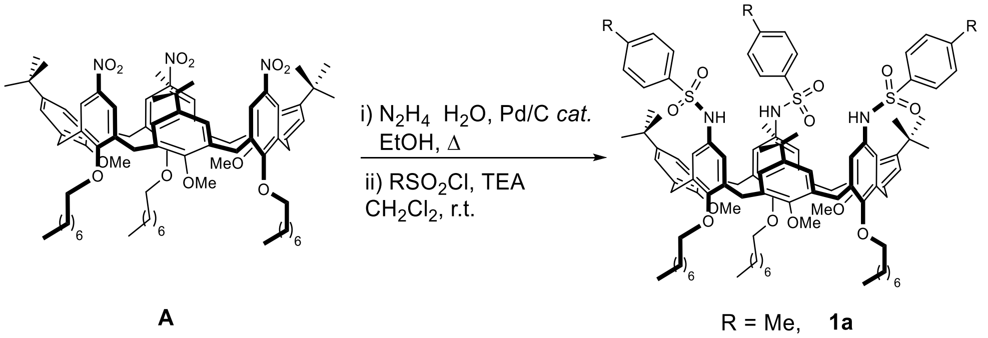


R = Me, **1a**
R = OMe, **1b**
R = Cl, **1c**
R = $NO_2$, **1d**

In a two-necked round bottomed flask, under $N_2$ atmosphere, Pd/C (10 mol %) was added to a suspension of **A** (0.3 mmol) in EtOH (200 ml). Subsequently, $NH_2NH_2$•$H_2O$ (6 mmol, 20 eq.) was added dropwise and the reaction placed in a oil-bath where it was refluxed at 95 °C for 24 hs. After completion determined by TLC analysis, the reaction was cooled-down to room temperature, and then filtered through a Celite pad to remove the Pd/C catalyst. The mixture was concentrated at reduced pressure and water (30 ml) was added. After extraction with $CH_2Cl_2$ (3 x 30 ml), organic phases were dried over $Na_2SO_4$ and concentrated at reduced pressure to afford a pale yellow solid. The crude was dissolved with dry $CH_2Cl_2$ (20 ml) under $N_2$ atmosphere and TEA (1.5 mmol, 5.0 eq.) was added. Subsequently, the corresponding arylsulphonylchloride (1.2 mmol, 4.0 eq.) was added at 0 °C. The reaction was stirred for 4 hs. After completion, a solution of sat. $NH_4Cl$ (20 ml) was added and the mixture extracted with $CH_2Cl_2$ (3 x 30 ml). The organic layers were dried over $Na_2SO_4$, concentrated at reduced pressure and the crude purified by column chromatography on silica gel (*n*-Hex/AcOEt: 80:20).

**1a.** Purification by column chromatography on silica gel (*n*-Hex/AcOEt 80:20) yielded **1a** (383 mg, 75%) as a white solid. **M. p.** = 127-130 °C. **$^1$H NMR** (400 MHz, $CDCl_3$) δ = 7.57 (d, *J* = 7.8 Hz, 6H, Ar-*H*), 7.21 (d, *J* = 8.0 Hz, 6H, Ar-*H*), 7.14 (bs, 6H, Ar-*H*), 6.21 (bs, 6H, Ar-*H*), 4.39 (d, *J* = 15.6 Hz, 6H, Ar-$CH_2$-Ar), 3.87 (t, *J* = 6.6 Hz, 6H, O$CH_2$($CH_2$)$_6$$CH_3$), 3.41 (d, *J* = 15.5 Hz, 6H, Ar-$CH_2$-Ar), 2.53 (bs, 9H, O$CH_3$), 2.41 (bs, 9H, Ar-$CH_3$), 1.90 – 1.79 (m, 6H, O O$CH_2$$CH_2$($CH_2$)$_5$$CH_3$), 1.59 – 1.48 (m, 6H, O($CH_2$)$_3$$CH_2$($CH_2$)$_3$$CH_3$), 1.37 (s, 27H, Ar-*tBu*), 1.35 – 1.19 (m, 24H, O($CH_2$)$_3$($CH_2$)$_4$$CH_3$), 0.90 (t, *J* = 6.5 Hz, 9H, O($CH_2$)$_7$$CH_3$). **$^{13}$C NMR** (101 MHz, $CDCl_3$) δ = 154.6 ($C_q$), 151.8 ($C_q$), 146.4 ($C_q$), 143.1 ($C_q$), 136.6 ($C_q$), 135.6 ($C_q$), 132.9 ($C_q$), 131.9 ($C_q$), 129.3 (CH), 127.8 (CH), 127.5 (CH), 120.7 (CH), 72.9 ($CH_2$), 60.0 ($CH_3$), 34.2 ($C_q$), 31.9 ($CH_3$), 31.6 ($CH_2$), 30.7 ($CH_2$), 30.5 ($CH_2$), 29.6 ($CH_2$), 29.3 ($CH_2$), 26.3 ($CH_2$), 22.7 ($CH_2$), 21.6 ($CH_3$), 14.1 ($CH_3$). **LC-MS**

(ESI): 1712.9 [M+Na]$^+$, 1728.9 [M+K]$^+$. **HR-MS** (ESI) *m/z*: [M+H]$^+$ calcd. for $C_{102}H_{136}N_3O_{12}S_3$ 1690.9286; found 1690.9280.

**1b.** Purification by column chromatography on silica gel (*n*-Hex/AcOEt 80:20→75:25) yielded **1b** (367 mg, 71%) as a white solid. **M. p.** = 134-137 °C. **$^1$H NMR** (400 MHz, $CDCl_3$) δ = 7.62 (d, *J* = 7.5 Hz, 6H), 7.18 – 7.13 (bs, 6H), 6.87 (d, *J* = 8.4 Hz, 6H), 6.26 – 6.18 (bs, 6H), 4.39 (d, *J* = 15.4 Hz, 6H), 3.89 – 3.82 (bs, 15H, $OCH_2(CH_2)_6CH_3$ + $ArOCH_3$), 3.41 (d, *J* = 15.5 Hz, 6H), 2.60 – 2.46 (bs, 9H), 1.91 – 1.80 (m, 6H), 1.61 – 1.48 (m, 6H), 1.38 (s, 21H), 1.37 – 1.25 (m, 24H), 0.96 – 0.84 (m, 9H). **$^{13}$C NMR** (101 MHz, $CDCl_3$) δ = 162.6 ($C_q$), 154.6 ($C_q$), 151.8 ($C_q$), 146.3 ($C_q$), 135.6 ($C_q$), 132.8 ($C_q$), 132.0 ($C_q$), 131.4 ($C_q$), 129.6 (CH), 127.8 (CH), 120.7 (CH), 113.8 (CH), 73.0 ($CH_2$), 60.0 ($CH_3$), 55.5 ($CH_3$), 34.2 ($C_q$), 31.9 ($CH_2$), 31.6 ($CH_3$), 30.7 ($CH_2$), 30.4 ($CH_2$), 29.6 ($CH_2$), 29.3 ($CH_2$), 26.3 ($CH_2$), 22.7 ($CH_2$), 14.1 ($CH_3$). **LC-MS** (ESI): 1760.6 [M+Na]$^+$, 1776.7 [M+K]$^+$. **HR-MS** (ESI) *m/z*: [M+H]$^+$ calcd. for $C_{102}H_{136}N_3O_{15}S_3$ 1738.9134; found 1738.9128.

**1c.** Purification by column chromatography on silica gel (*n*-Hex/AcOEt 80:20) yielded **1c** (322 mg, 61%) as a white solid. **M. p.** = 131-134 °C**.** **$^1$H NMR** (400 MHz, $CDCl_3$) δ = 7.62 (d, *J* = 8.6 Hz, 6H), 7.39 (d, *J* = 8.6 Hz, 6H), 7.15 (bs, 6H), 6.15 (bs, 6H), 4.37 (d, *J* = 15.7 Hz, 6H), 3.88 (t, *J* = 6.4 Hz, 6H), 3.43 (d, *J* = 15.7 Hz, 6H), 2.58 (bs, 9H), 1.94 – 1.82 (m, 6H), 1.61 – 1.50 (m, 6H), 1.38 (s, 27H), 1.33 – 1.11 (m, 24H), 0.90 (t, *J* = 6.8 Hz, 9H). **$^{13}$C NMR** (101 MHz, $CDCl_3$) δ = 154.5 ($C_q$), 152.1 ($C_q$), 146.6 ($C_q$), 139.0 ($C_q$), 137.8 ($C_q$), 135.9 ($C_q$), 132.8 ($C_q$), 131.3 ($C_q$), 129.0 (2xCH), 127.7 (CH), 120.5 (CH), 72.9 ($CH_2$), 60.0 ($CH_3$), 34.2 ($C_q$), 31.9 ($CH_2$), 31.6 ($CH_3$), 30.8 (2x $CH_2$), 29.6 ($CH_2$), 29.3 ($CH_2$), 26.3 ($CH_2$), 22.7 ($CH_2$), 14.1 ($CH_3$). **LC-MS** (ESI): 1772.8 [M+Na]$^+$, 1788.7 [M+K]$^+$. **HR-MS** (ESI) *m/z*: [M+H]$^+$ calcd. for $C_{99}H_{127}Cl_3N_3O_{12}S_3$1750.7647; found 1750.7642.

**1d**. Purification by column chromatography on silica gel (*n*-Hex/AcOEt 85:15→80:20) yielded **1d** (300 mg, 56%) as yellow solid. **M. p.** = 128-131 °C. **$^1$H NMR** (400 MHz, $CDCl_3$) δ = 8.28 (d, *J* = 8.8 Hz, 6H), 7.88 (d, *J* = 8.7 Hz, 6H), 7.15 (bs, 6H), 6.17 (bs, 6H), 4.37 (d, *J* = 15.7 Hz, 6H), 3.88 (t, *J* = 6.5 Hz, 6H), 3.45 (d, *J* = 15.8 Hz, 6H), 2.64 (bs, 9H), 1.93 – 1.83 (m, 6H), 1.65 – 1.51 (m, 6H), 1.44 – 1.24 (m, 24H), 1,34 (s, 27H), 0.89 (t, *J* = 6.8 Hz, 9H). **$^{13}$C NMR** (101 MHz, $CDCl_3$) δ = 154.5 ($C_q$), 152.7 ($C_q$), 150.1 ($C_q$), 146.8 ($C_q$), 144.8 ($C_q$), 136.3 ($C_q$), 132.8 ($C_q$), 130.5 ($C_q$), 128.8 (CH), 127.7 (CH), 124.0 (CH), 120.7 (CH), 73.0 ($CH_2$), 60.0 ($CH_3$), 34.2 ($C_q$), 31.9 ($CH_2$), 31.5 ($CH_3$), 30.8 ($CH_2$), 30.5 ($CH_2$), 29.6 ($CH_2$), 29.3 ($CH_2$), 26.3 ($CH_2$), 22.7 ($CH_2$), 14.1 ($CH_3$). **LC-MS** (ESI) *m/z*: 1805.8 [M+Na]$^+$, 1821.6 [M+K]$^+$. **HR-MS** (ESI) *m/z*: [M+$NH_4$]$^+$ calcd. for $C_{99}H_{130}N_7O_{18}S_3$ 1800.8634; found 1800.8629.

## Gram-scale synthesis of TSA calixarenes 1a

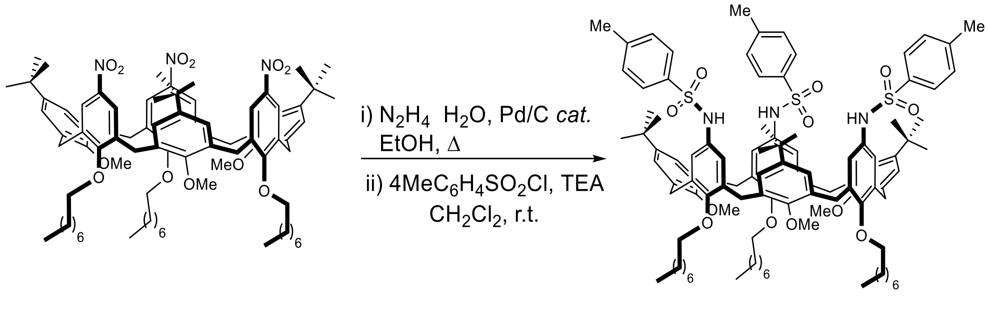


**A** **1a**

In a two-necked round bottomed flask, under $N_2$ atmosphere, Pd/C (10 mol %) was added to a suspension of **A** (1.0 g, 0.8 mmol) in EtOH (500 ml). Subsequently, $NH_2NH_2$•$H_2O$ (16 mmol, 20 eq.) was added dropwise and the reaction placed in a oil-bath where it was refluxed at 95 °C for 24 hs. After completion determined by TLC analysis, the reaction was cooled-down to room temperature, and then filtered through a Celite pad to remove the Pd/C catalyst. The mixture was concentrated at reduced pressure and water (50 ml) was added. After extraction with $CH_2Cl_2$ (3 x 50 ml), organic phases were dried over $Na_2SO_4$ and concentrated at reduced pressure to afford a pale yellow solid. The crude was dissolved with dry $CH_2Cl_2$ (50 ml) under $N_2$ atmosphere and TEA (600 µl, 4.0 mmol, 5.0 eq.) was added. Subsequently, the *para*-toluenesulphonylchloride (530 mg, 2.8 mmol, 3.5 eq.) was added portionwise over 10 min at 0 °C. The reaction was stirred for 4 hs. After completion, a solution of sat. $NH_4Cl$ (50 ml) was added and the mixture extracted with $CH_2Cl_2$ (3 x 50 ml). The organic layers were dried over $Na_2SO_4$, concentrated at reduced pressure and the crude purified by column chromatography on silica gel (*n*-Hex/AcOEt: 80:20) to yield **1a** (1.0 g, 74%) as a white solid ($^1$H-NMR, 400 MHz, $CDCl_3$).

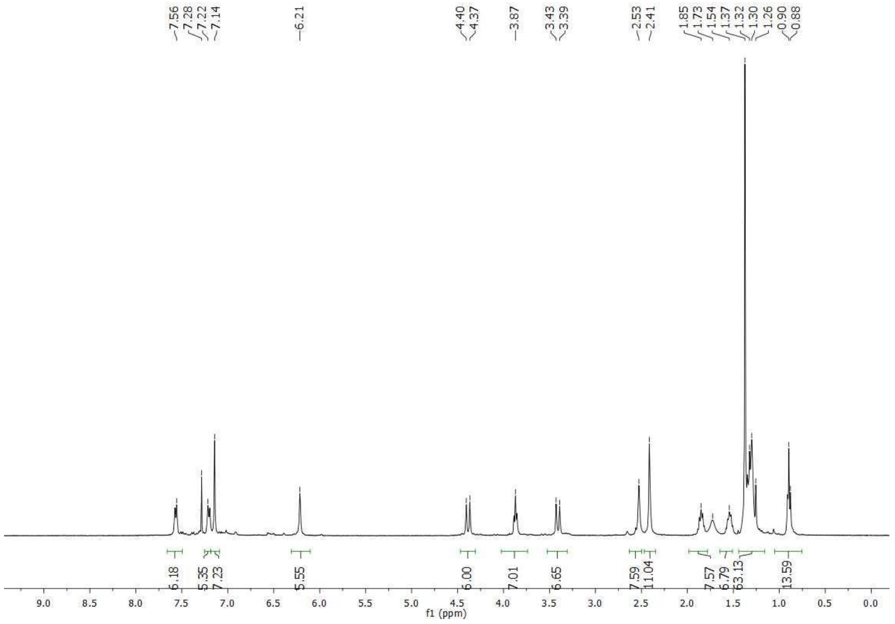

## NMR analysis of model pseudorotaxane P[1a(*p*C)⊃DOV]2OTs

**Figure S1.** Mid-field expanded region of the 2D HSQC NMR ($CDCl_3$, 600 MHz) spectrum of P[**1a**(*p*C)⊃DOV]2OTs showing the downfield shift of $^{13}$C-NMR resonances of methylene protons a/a' due to a partial-cone configuration. The circles identify N-$CH_2$ methylene protons of bipyridinium axle.

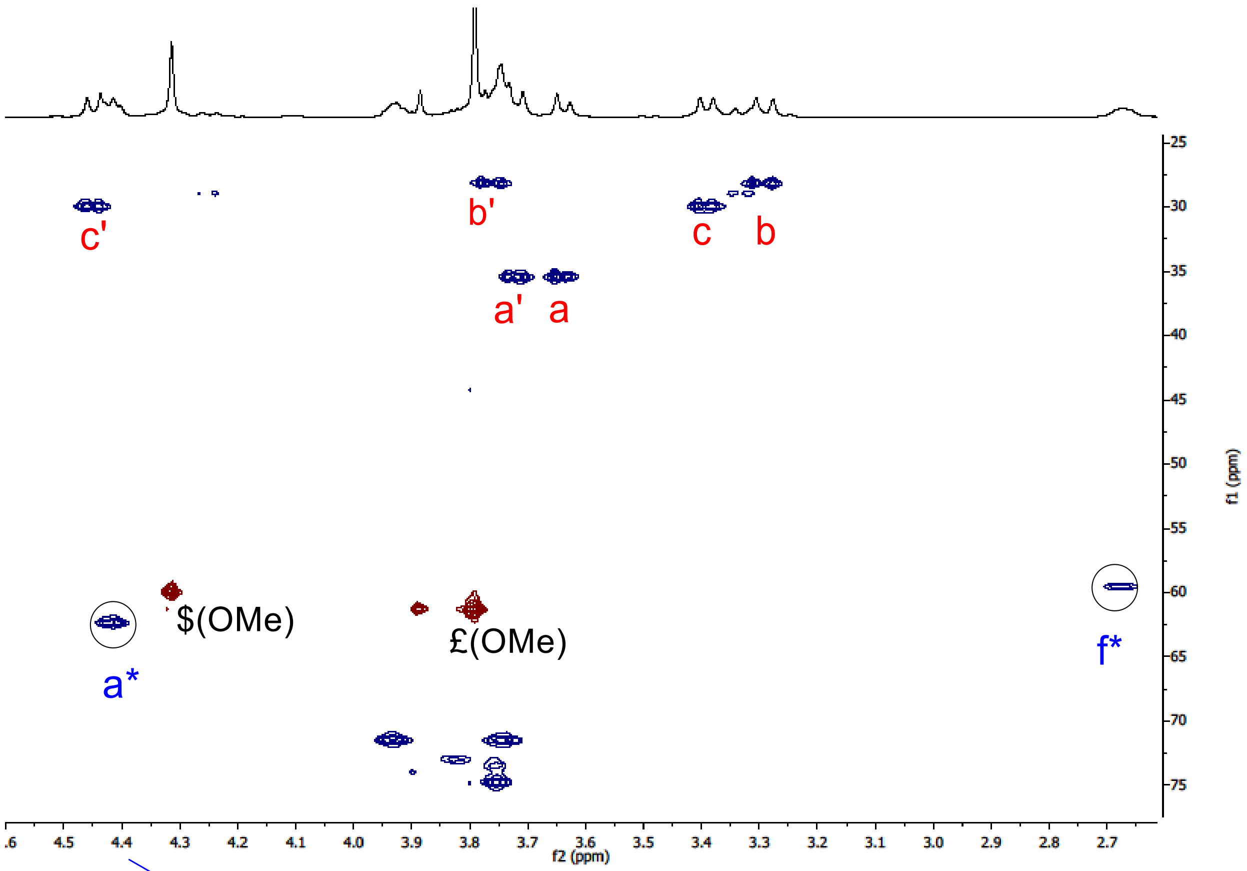


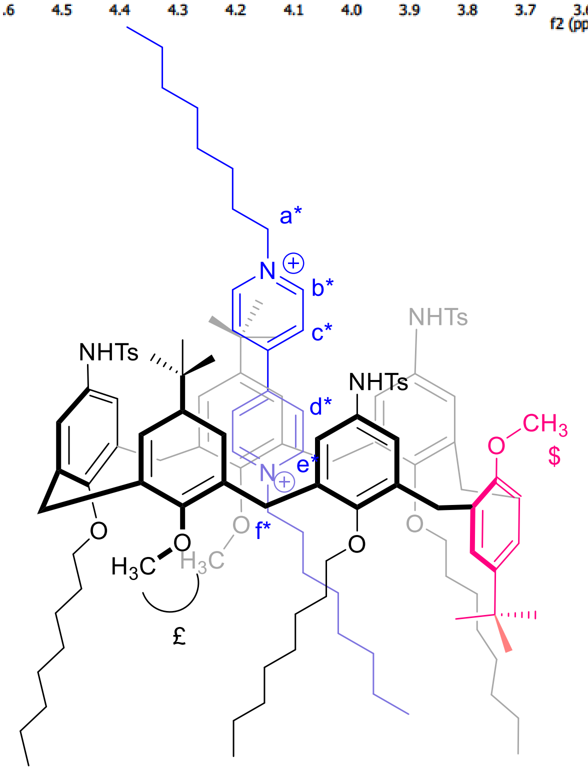


=

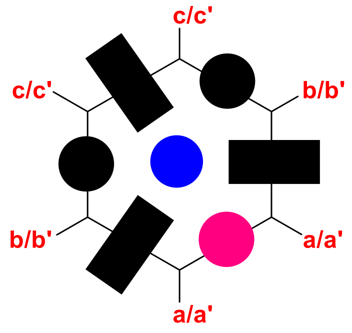

**Figure S2.**·Mid-field expanded region of the 2D $^{1}$H-$^{1}$H COSY NMR ($CDCl_3$, 600 MHz) spectrum of P[**1a**(*p*C)⊃DOV]2OTs showing the *J*-coupling correlations of the macrocycle “bridging” equatorial and axial methylene protons.

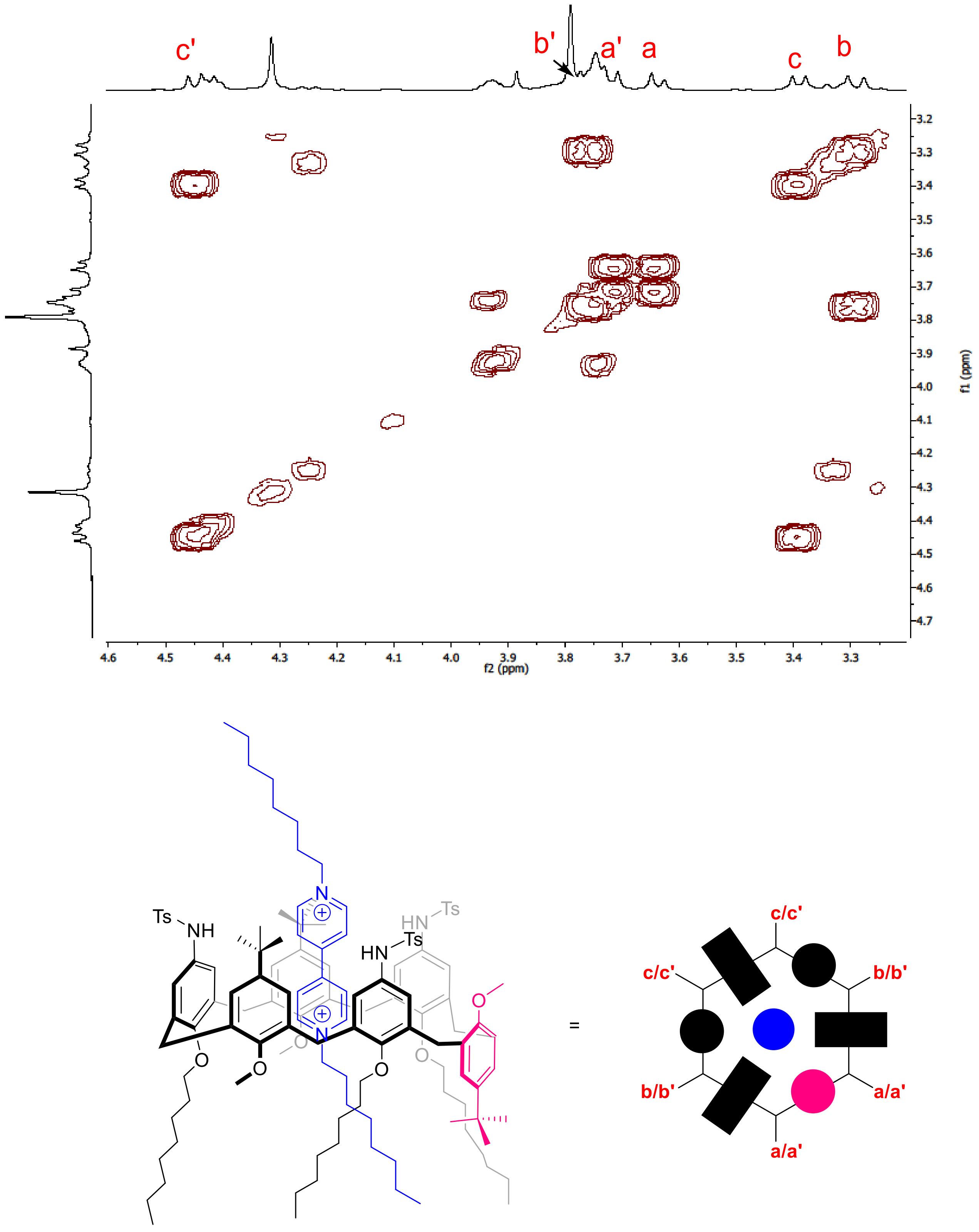

**Figure S3.**·Low-field expanded region of the 2D $^{1}H$-$^{1}H$ COSY NMR ($CDCl_3$, 400 MHz) spectrum of P[**1a**(*p*C)⊃DOV]2OTs showing the *J*-coupling correlations between the aromatic protons of calixarene **1a** .

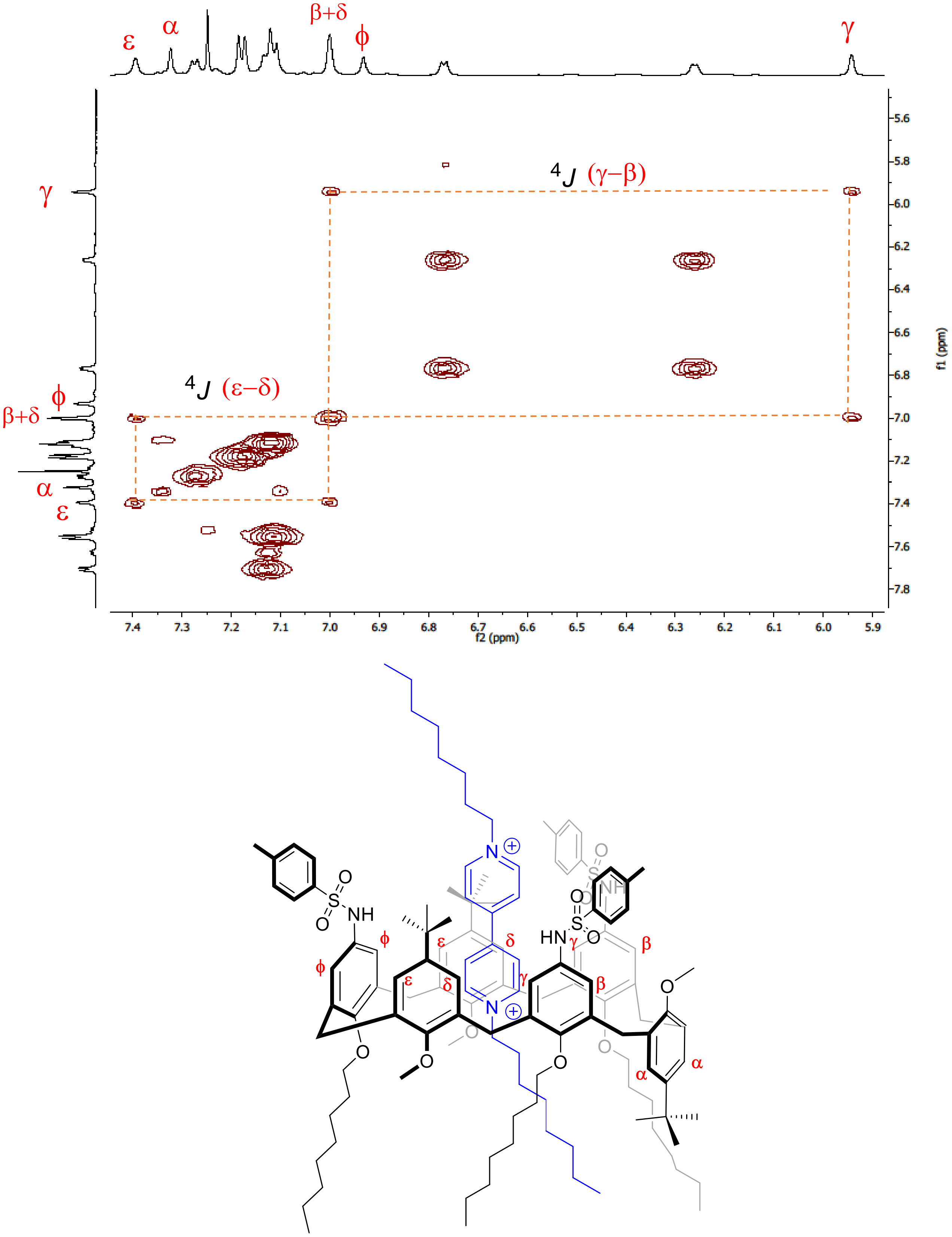

**Figure S4.** Low-field expanded region of the 2D ROESY-NMR ($CDCl_3$, 600 MHz, spin-lock = 200 ms) spectrum of P[**1a**(*p*C)⊃DOV]2OTs showing the dipolar correlations of the aromatic protons of calixarene **1a** with methylene protons:

- α correlate with a/a’
- ε correlate with c
- β correlate with a/a’
- δ correlate with b

Correlations of α with t-Bu in purple; correlation of ε and δ with 2t-Bu in green.

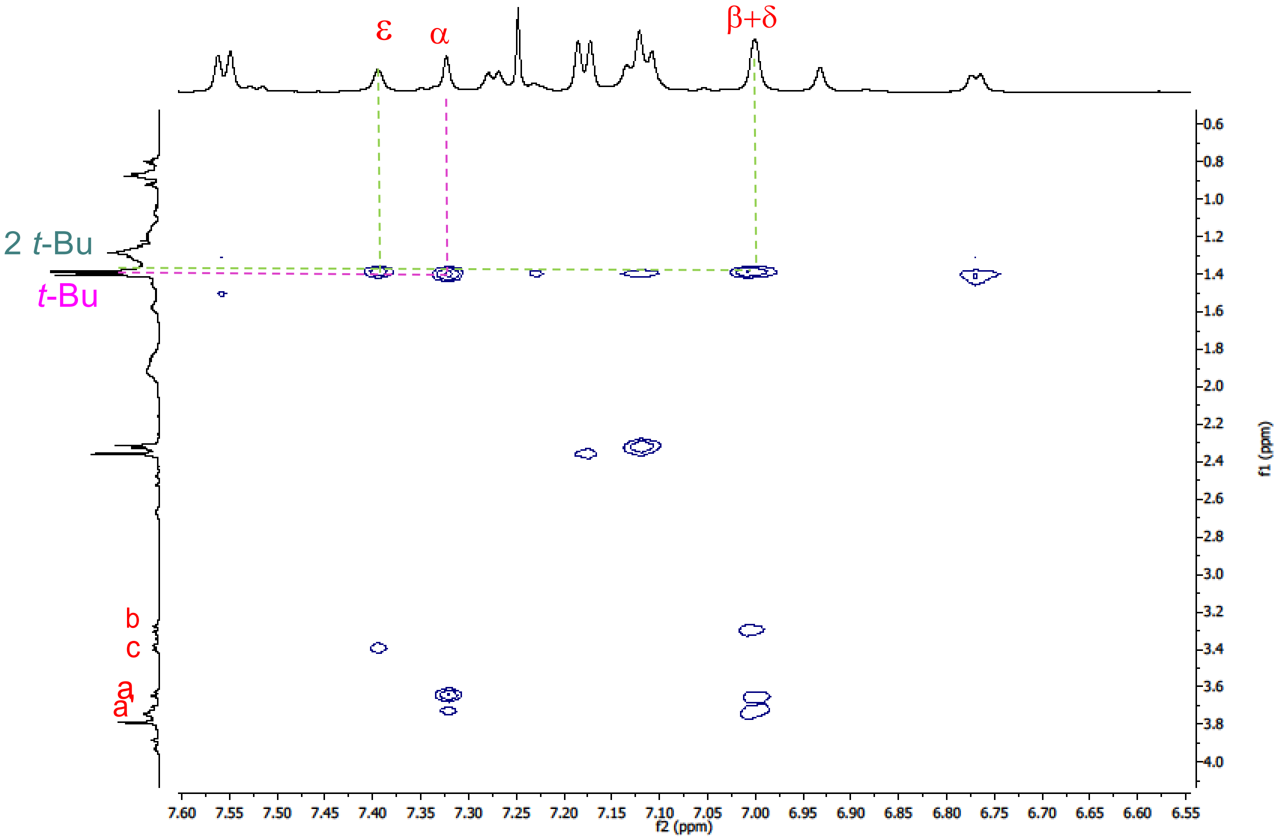


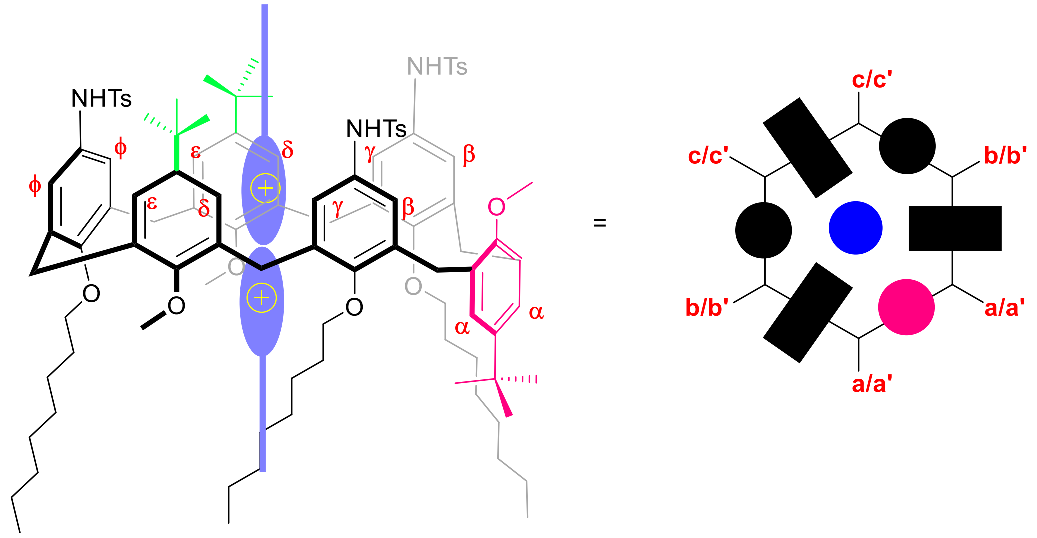

**Figure S5.** Low-field expanded region of the 2D ROESY-NMR ($CDCl_3$, 600 MHz, spin-lock = 200 ms) spectrum of P[**1a**(*p*C)⊃DOV]2OTs showing the dipolar correlations of the aromatic protons of calixarene **1a** with methylene protons and *t*-Bu. A ROE contact between the aromatic CH e* of DOV salt and *t*-Bu is highlighted in red.

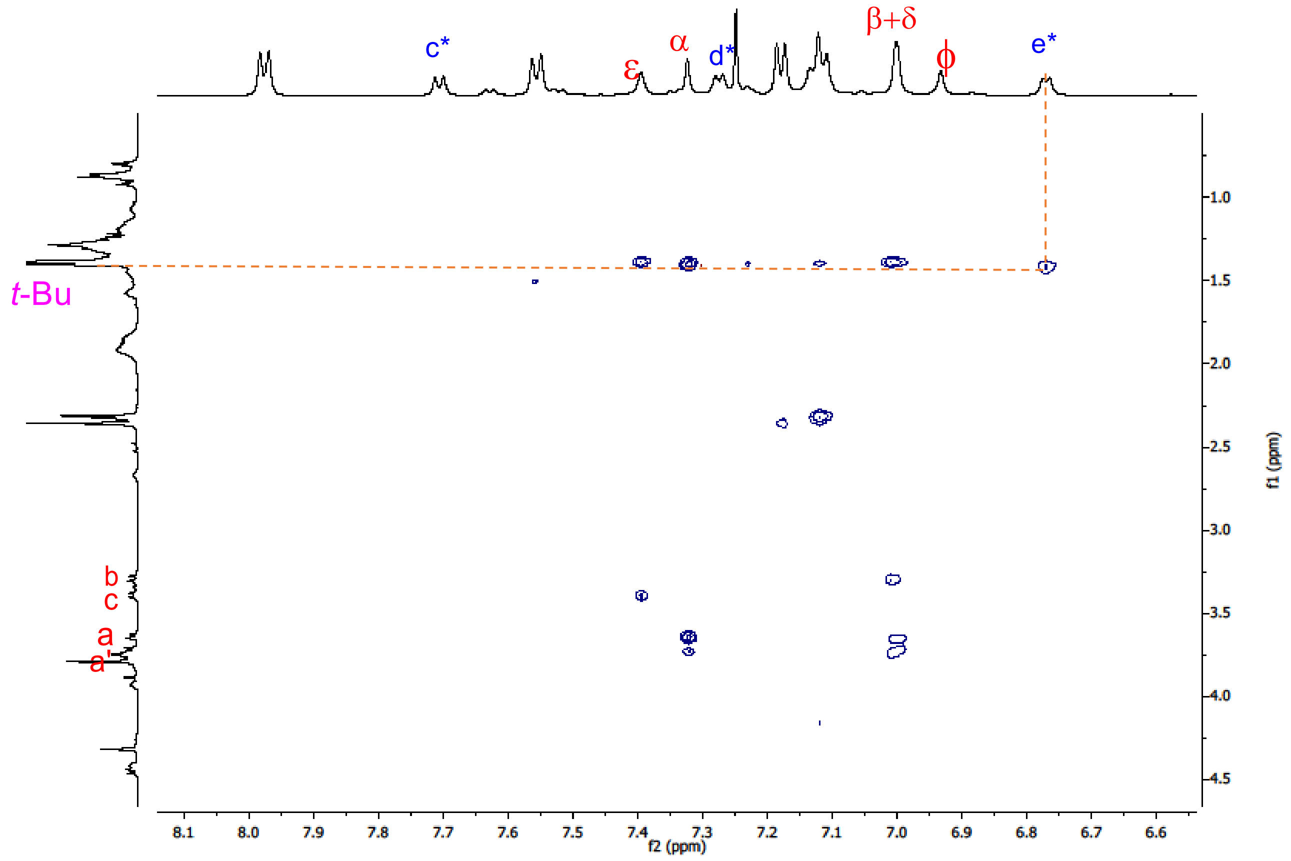


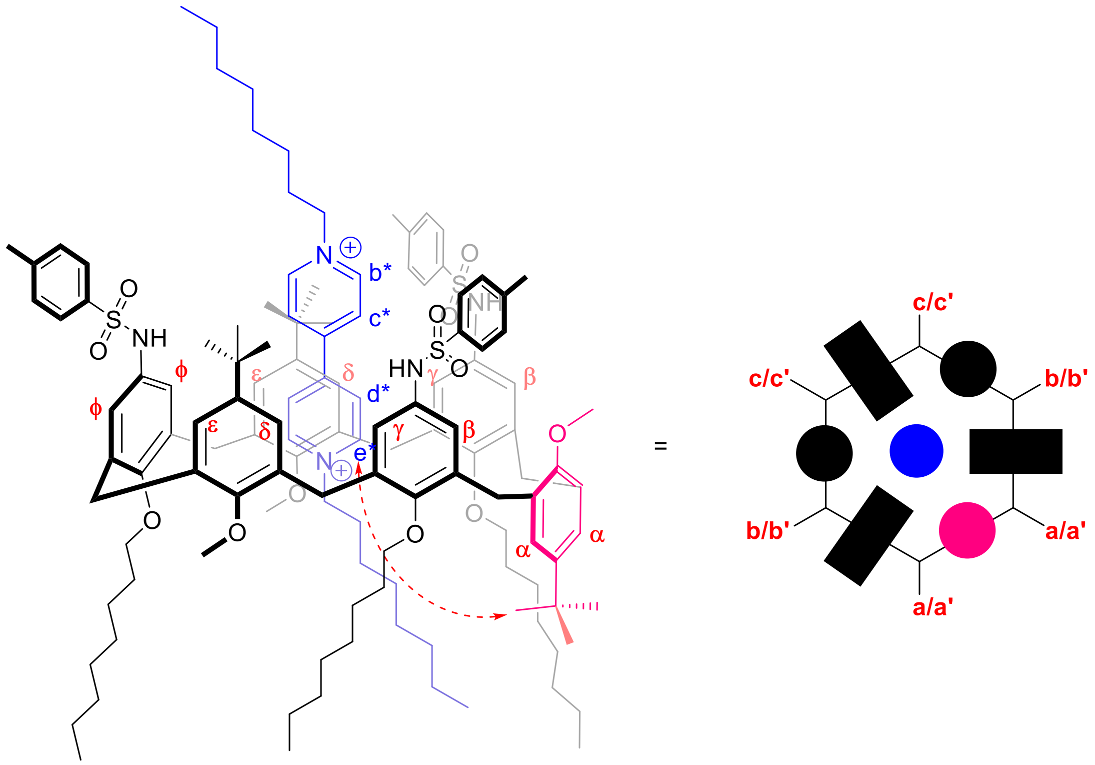

**Figure S6.** Expanded region of the 2D ROESY-NMR ($CDCl_3$, 600 MHz, spin-lock = 200 ms) spectrum of P [**1a**(*p*C)⊃DOV]2OTs showing the spatial correlation between ϕ and ε proton signals.

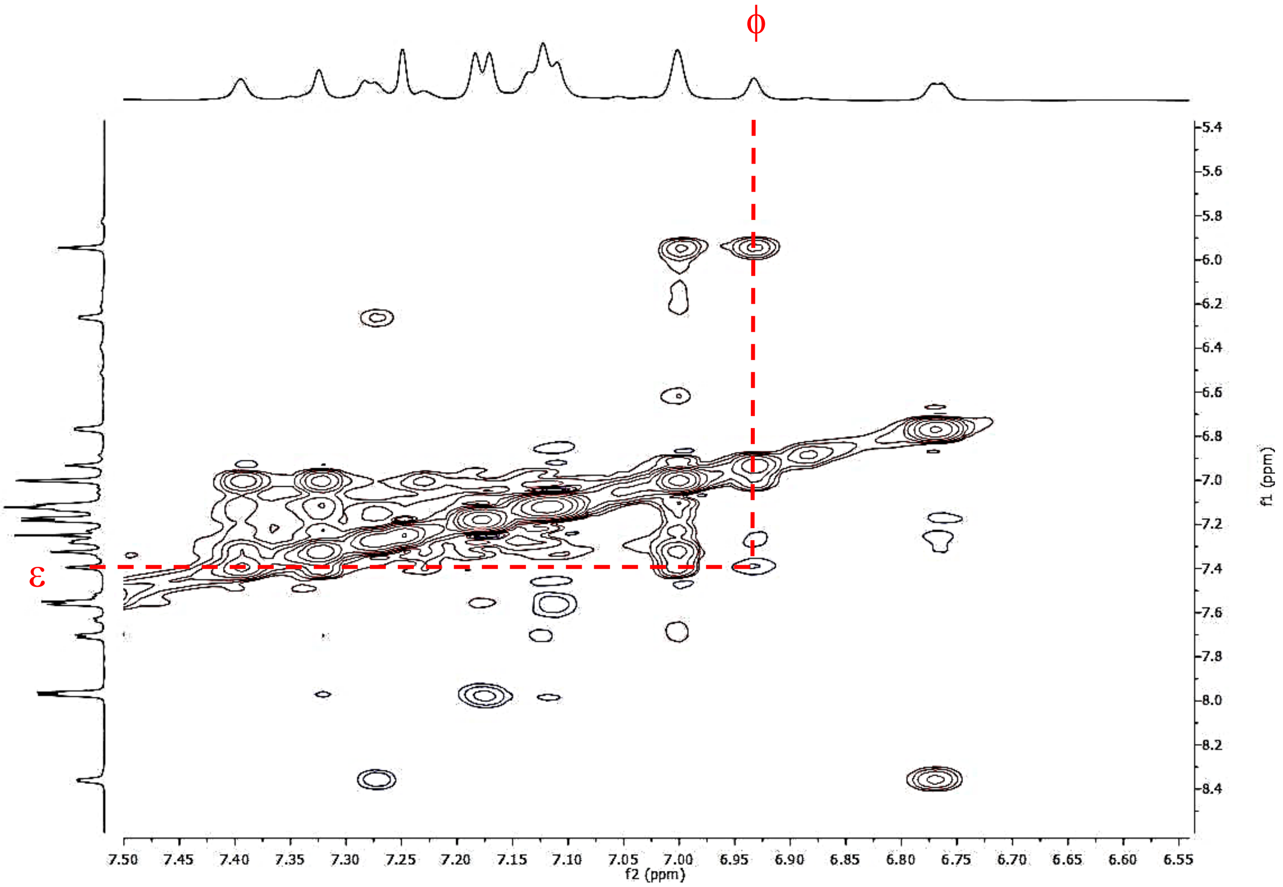


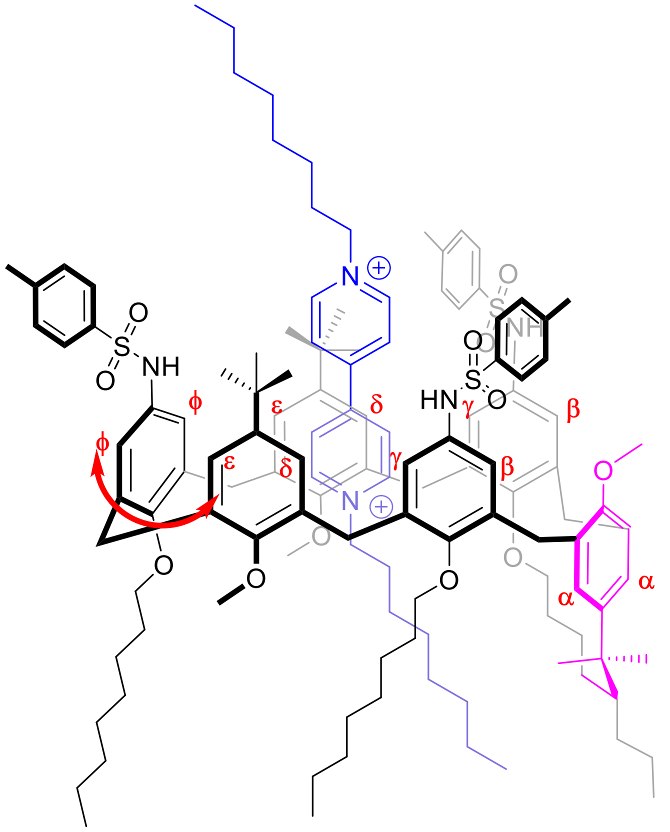

**Figure S7.** Expanded region of the 2D ROESY-NMR ($CDCl_3$, 600 MHz, spin-lock = 200 ms) spectrum of P [**1a**(*p*C)⊃DOV]2OTs showing ROE contacts between: 1) the aromatic CH β of calixarene **1a** and OMe $ (in red); 2) the aromatic CH δ with equatorial methylene CH b (in green).

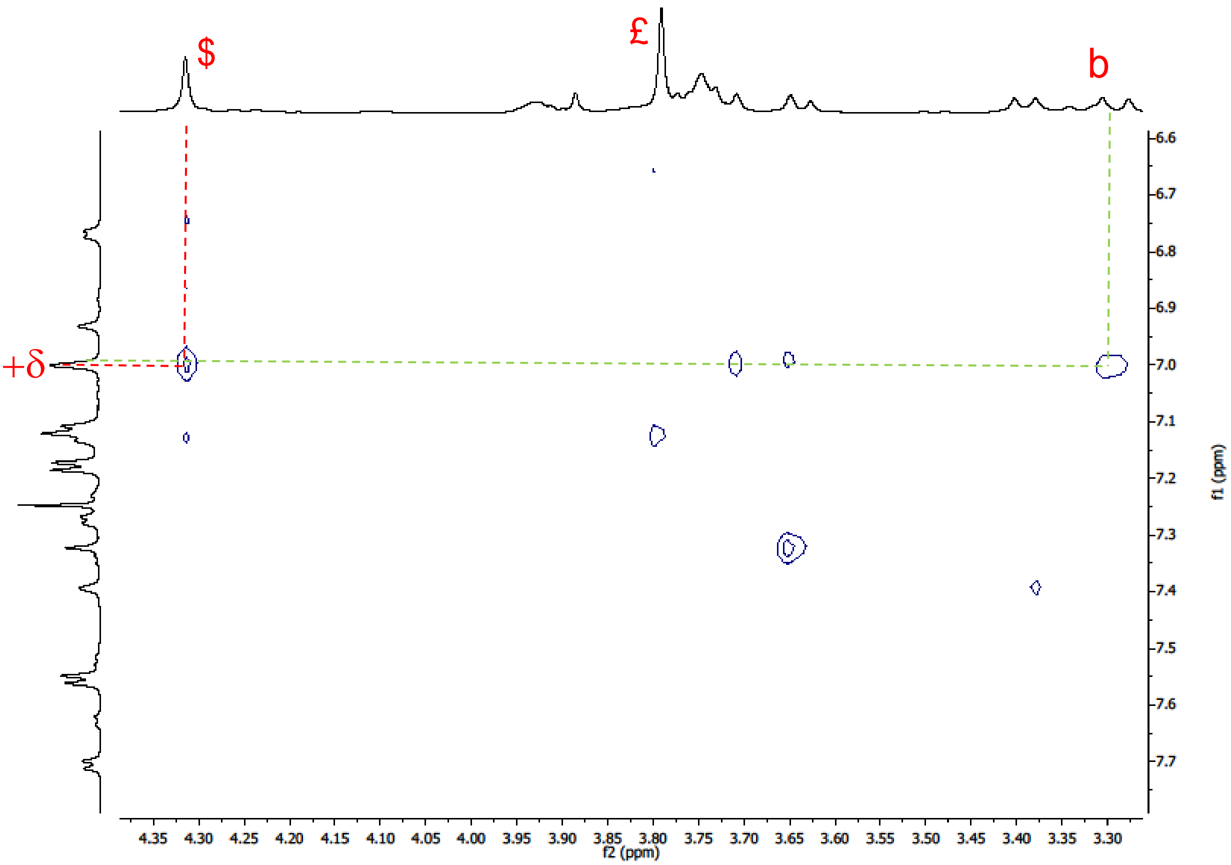


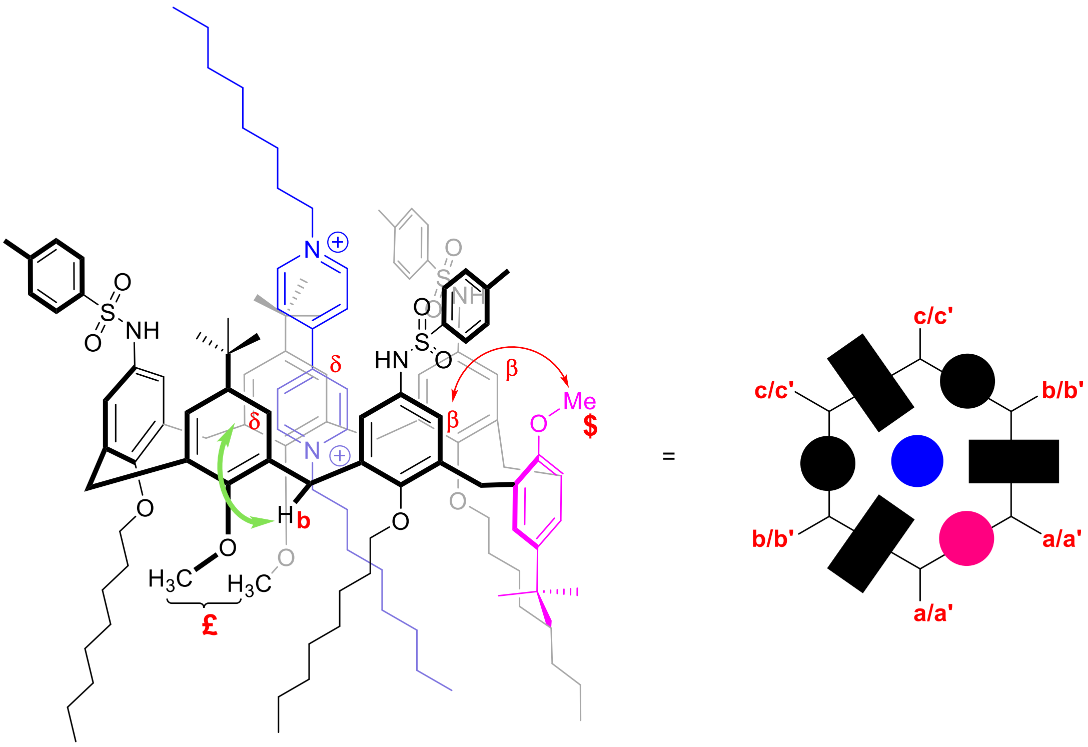

**Figure S8.** 1D-ROESY spectra recorded with selective irradiation of aromatic CH resonance of DOV salt at 6.26 (**d***) and 6.77 (**e***) ppm. The observed ROE contacts are indicated with the corresponding colors (green, red) in the schematic representation of the complexes.

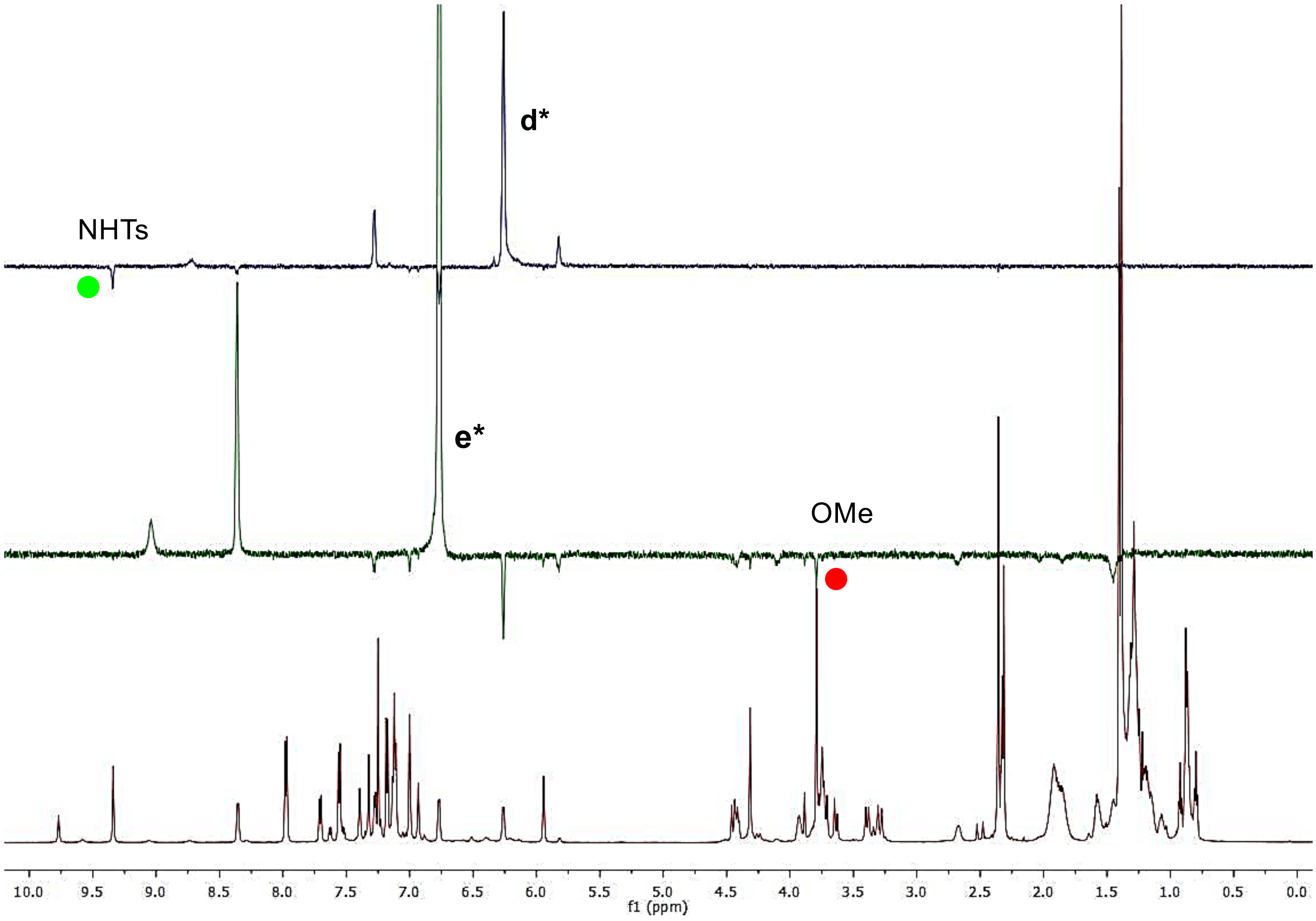


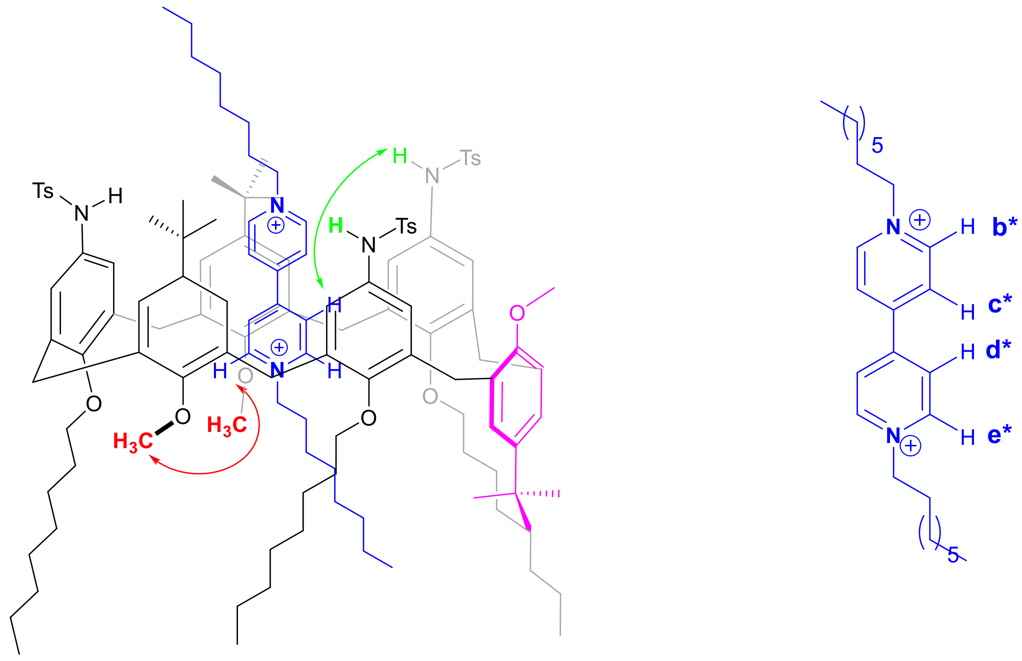

## NMR analysis of model pseudorotaxane P[1a(C)⊃DOV]2I

**Figure S9.** Mid-field expanded region of the 2D HSQC NMR ($CDCl_3$, 400 MHz) spectrum of P[**1a**(C)⊃DOV]2I showing the resonances of methylene protons a/a' due to a cone configuration. The circles identify N-$CH_2$ methylene protons of complexed bipyridinium axle.

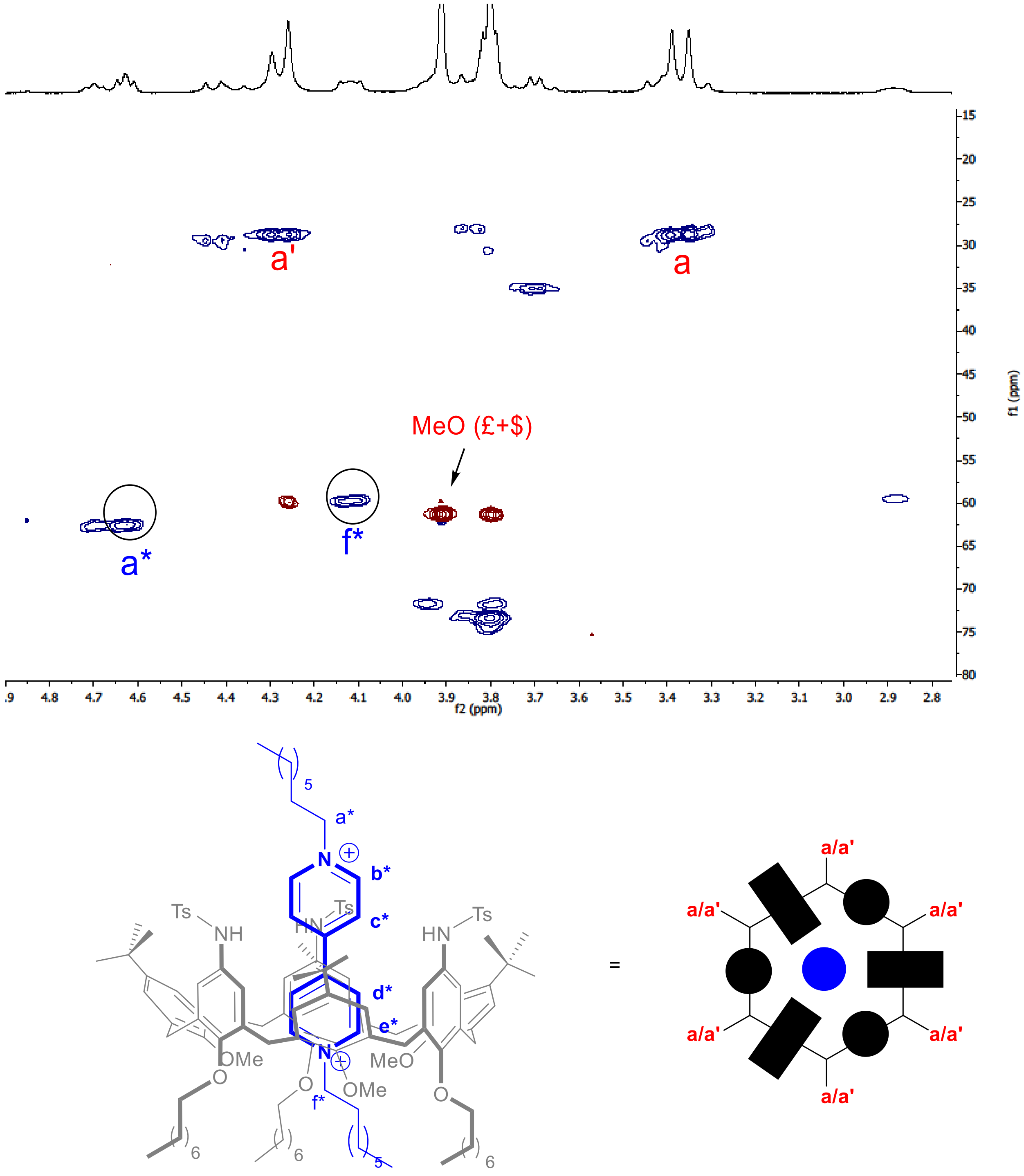

**Figure S10.** Low-field expanded region of the 2D $^{1}H$-$^{1}H$ COSY NMR ($CDCl_3$, 400 MHz) spectrum of P[**1a**(C)⊃DOV]2I showing the *J*-coupling correlations between the aromatic protons of bipyridinium axle**.** Aromatic protons of TSA calixarene in cone conformations (α, β) are also shown.

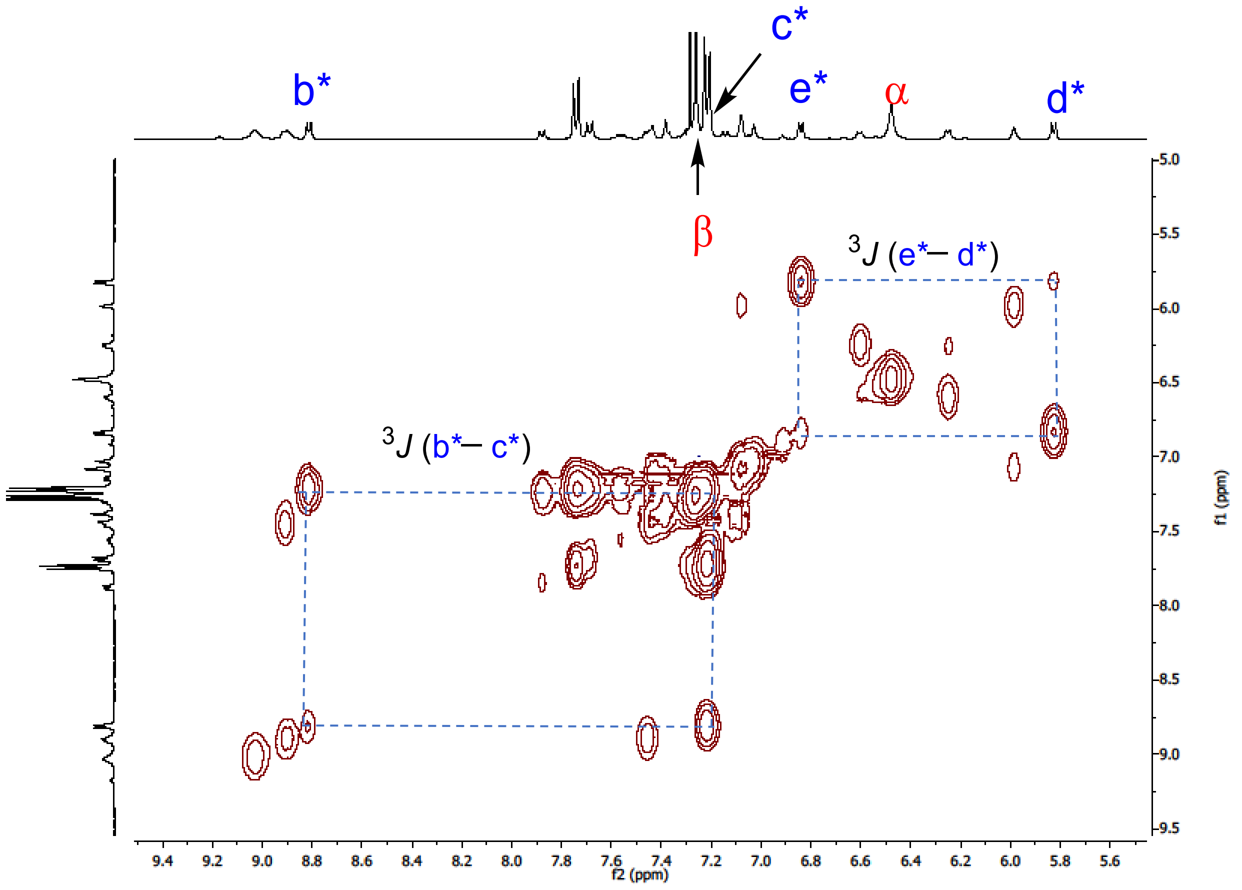


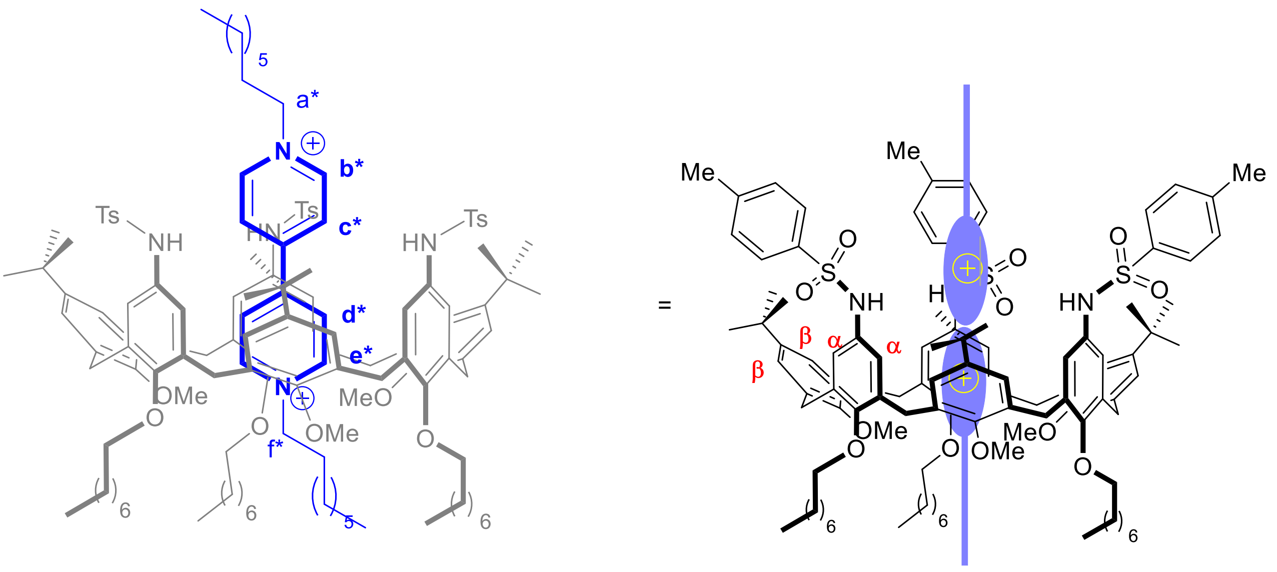

**Figure S11.** Expanded region of [1]H-NMR ($CDCl_3$, 400 MHz) of P[**1**(*p*C/C)⊃DOV]2OTs. The ratio of pseudorotaxanes in solution was given by integration of N-$CH_2$ signals of complexed dioctylviologen salts.

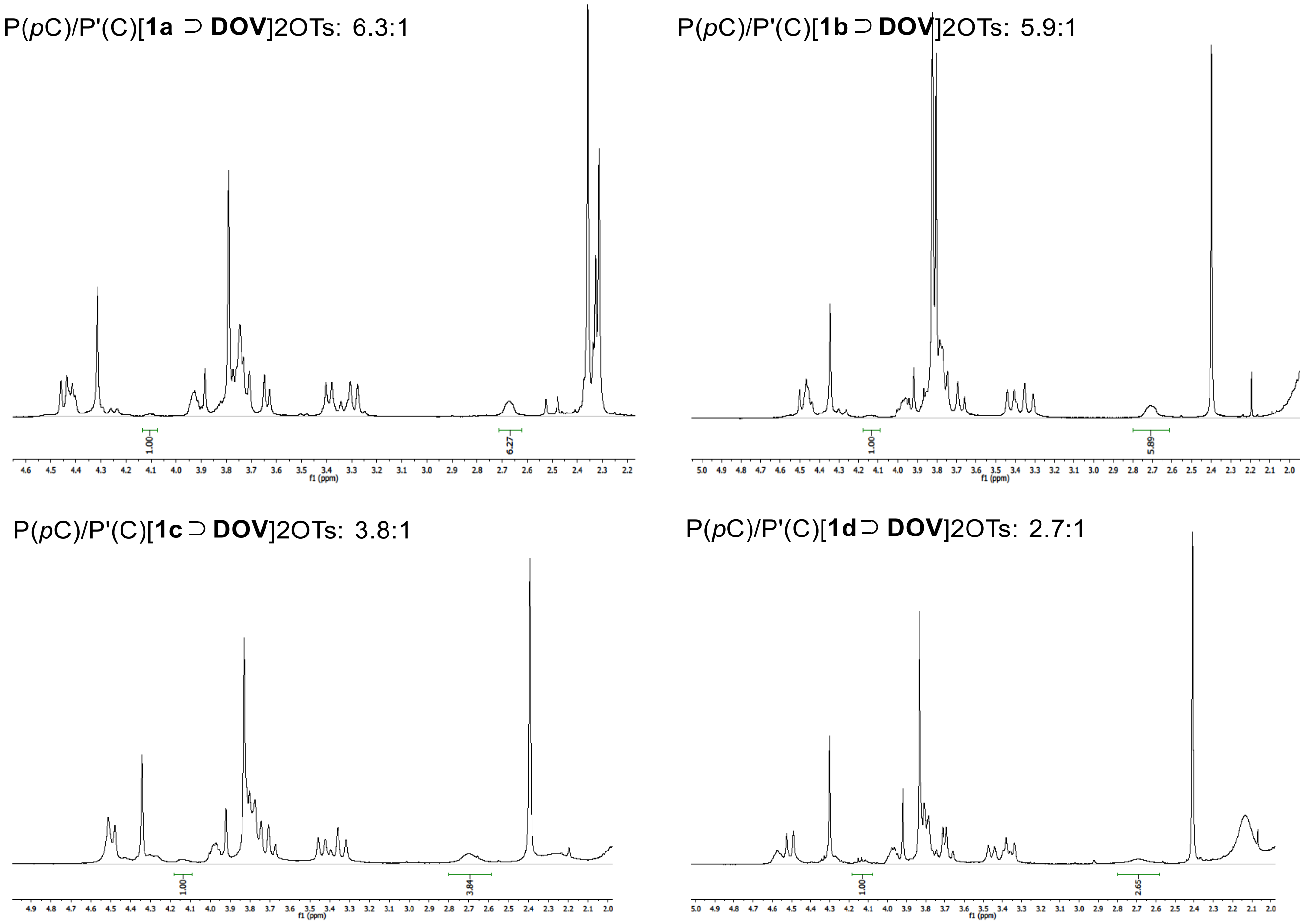

**Figure S12.** Expanded region of $^{1}$H-NMR ($CDCl_3$, 400 MHz) of P[**1a**(*p*C/C)⊃DOV]2X. The ratio of pseudorotaxanes in solution was given by integration of N-$CH_2$ signals of complexed dioctylviologen salts.

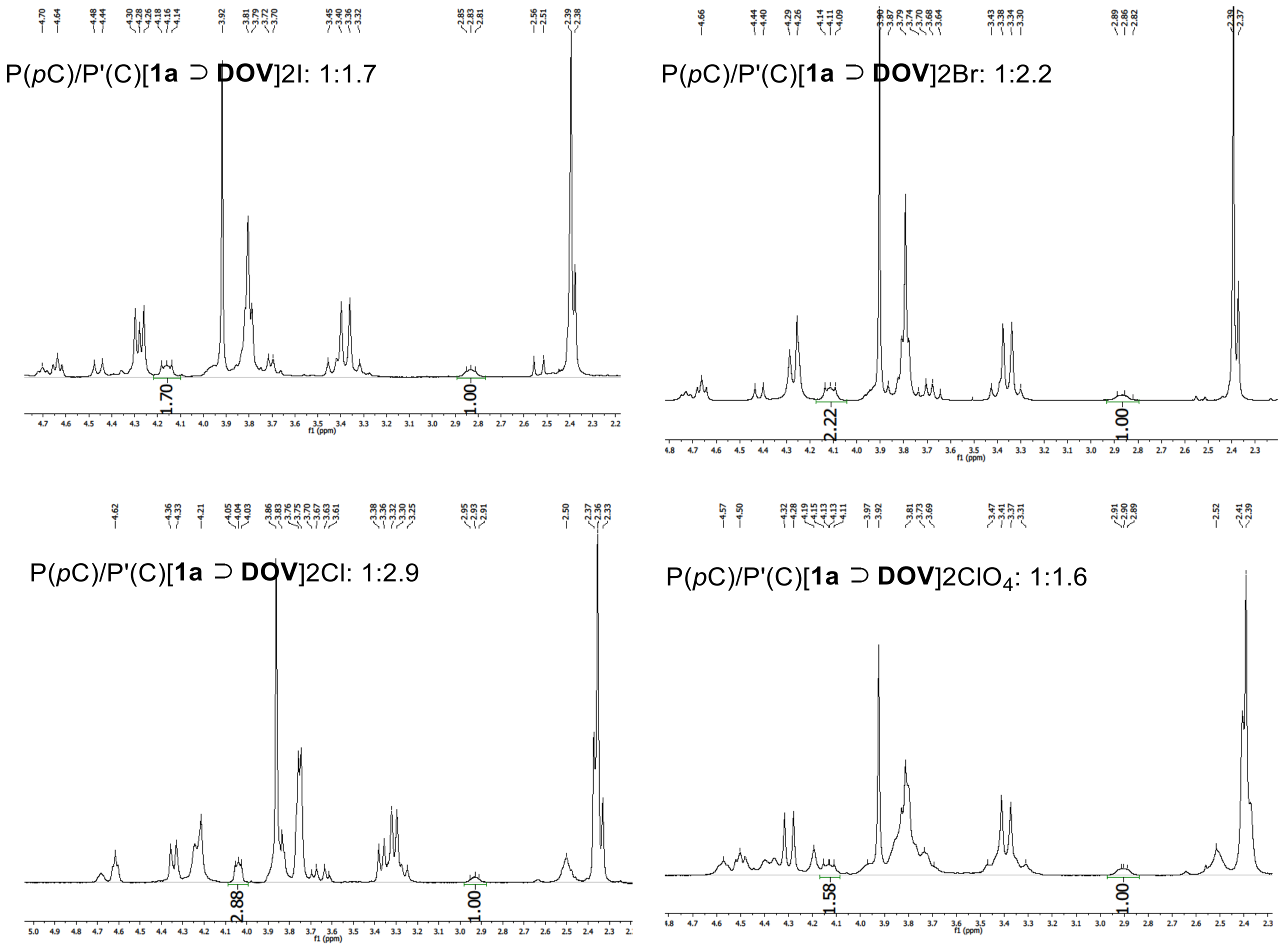

# NMR Spectra

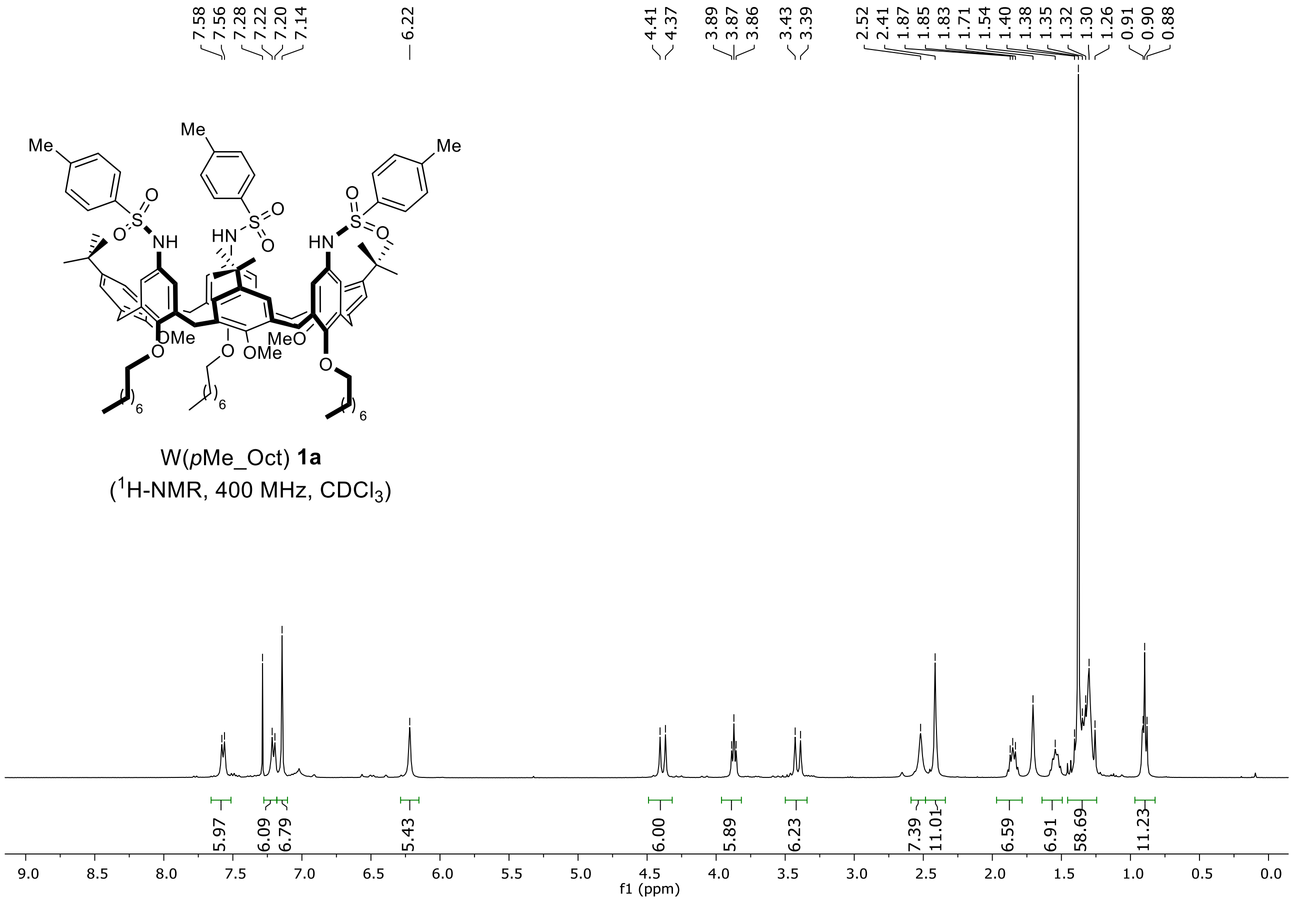


W(*p*Me_Oct) **1a**
($^{1}$H-NMR, 400 MHz, $CDCl_3$)

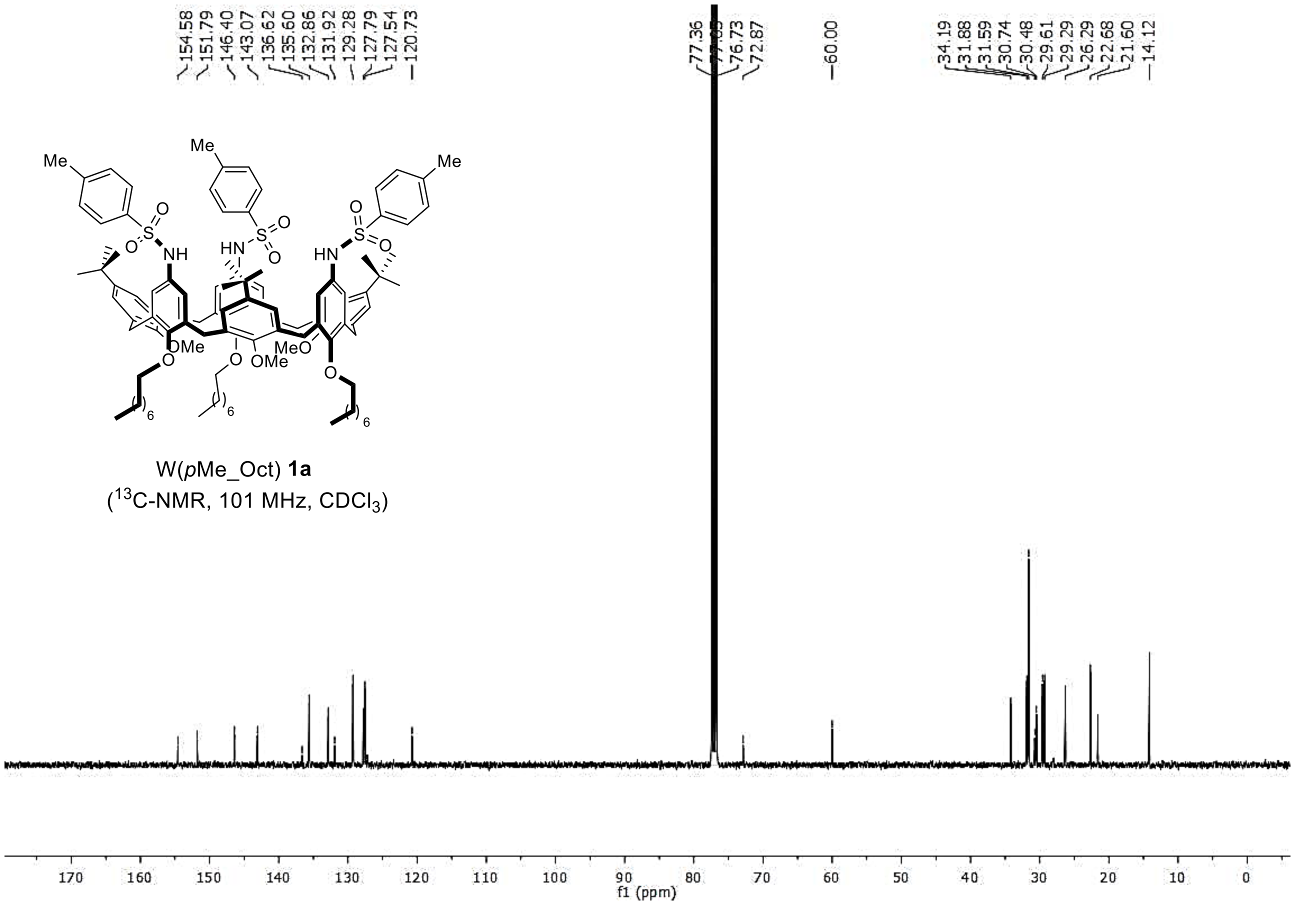


W(*p*Me_Oct) **1a**
($^{13}$C-NMR, 101 MHz, $CDCl_3$)

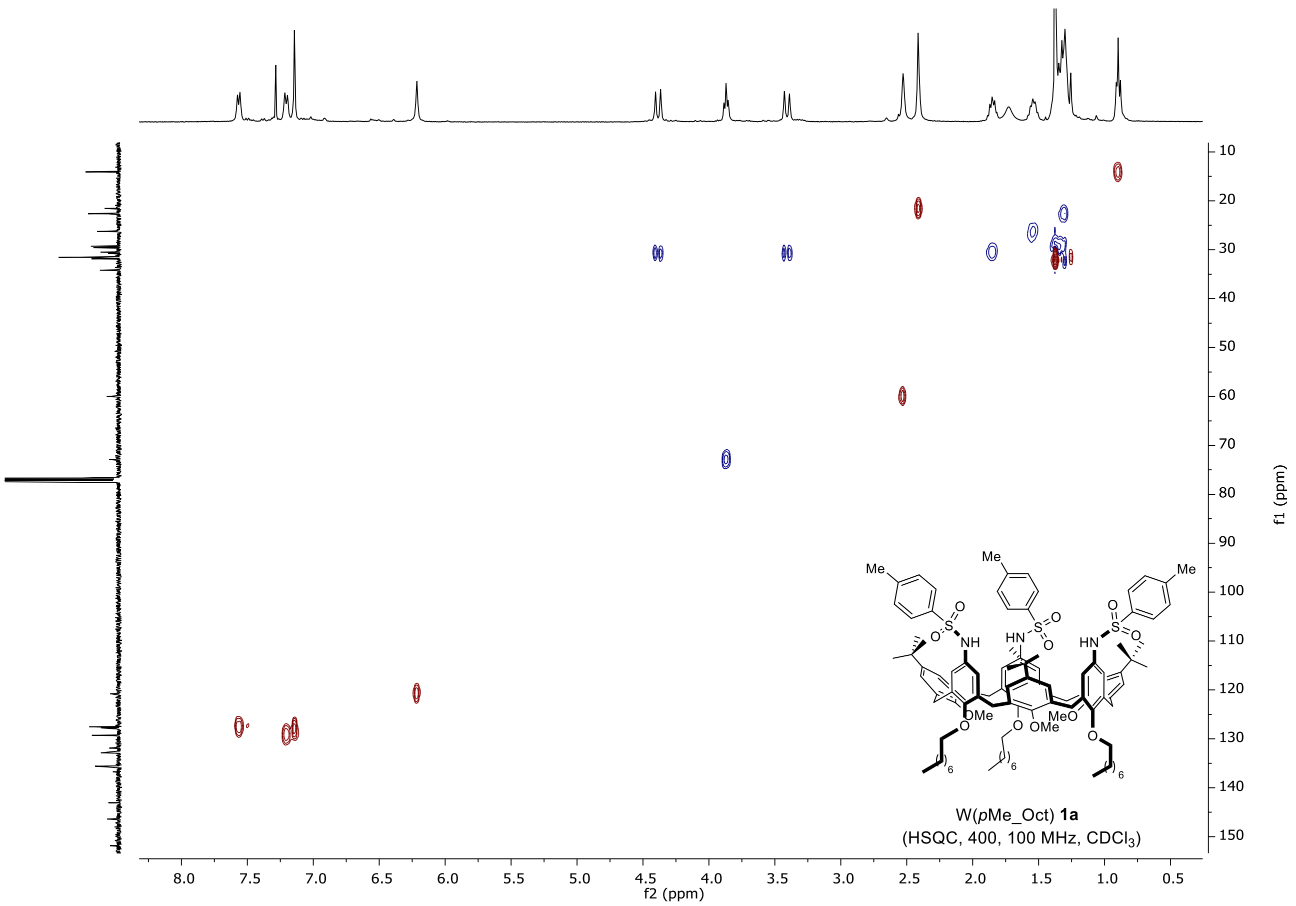
W(pMe_Oct) 1a
(HSQC, 400, 100 MHz, CDCl3)
f2 (ppm)
f1 (ppm)

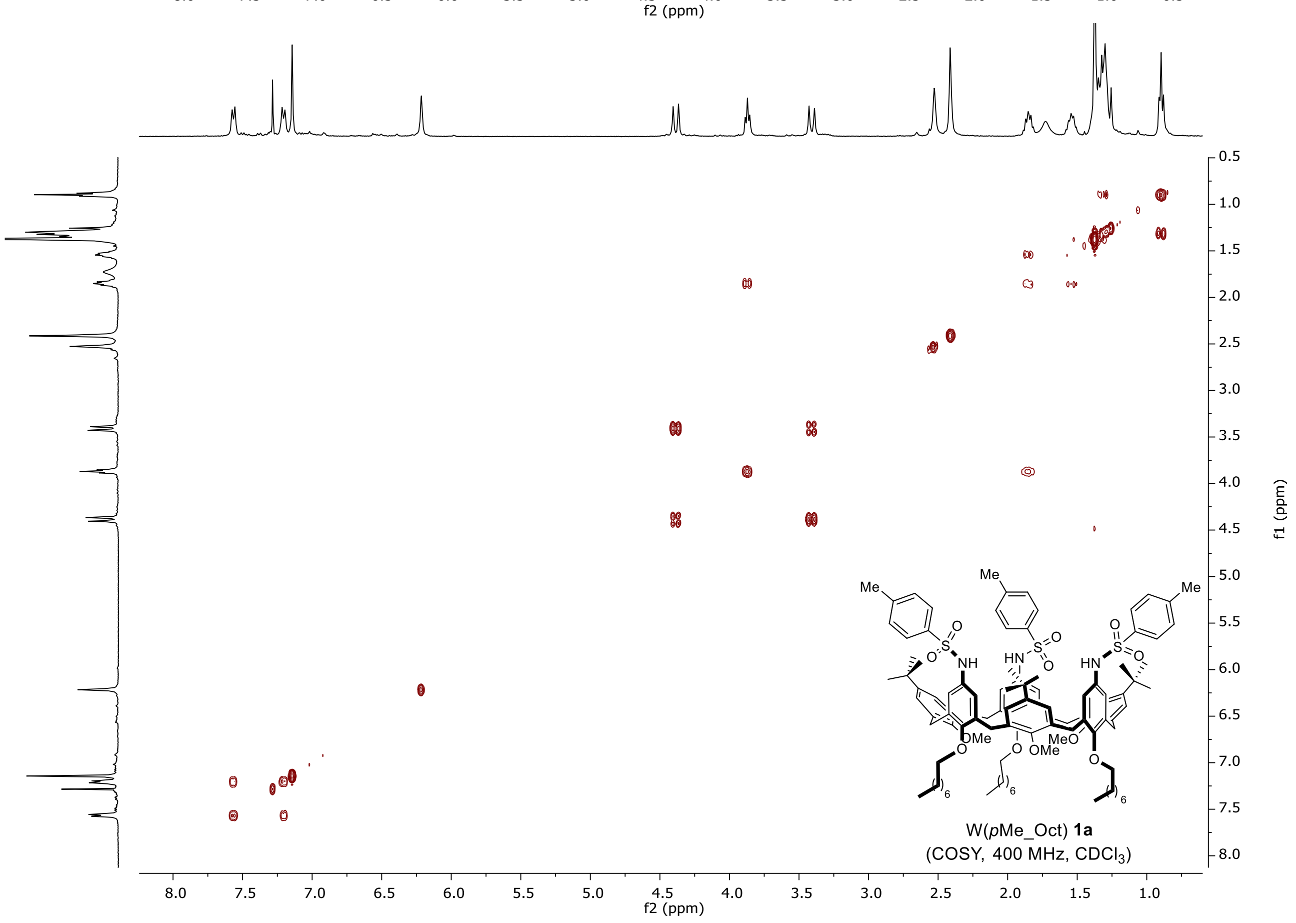
W(pMe_Oct) 1a
(COSY, 400 MHz, CDCl3)
f2 (ppm)
f1 (ppm)

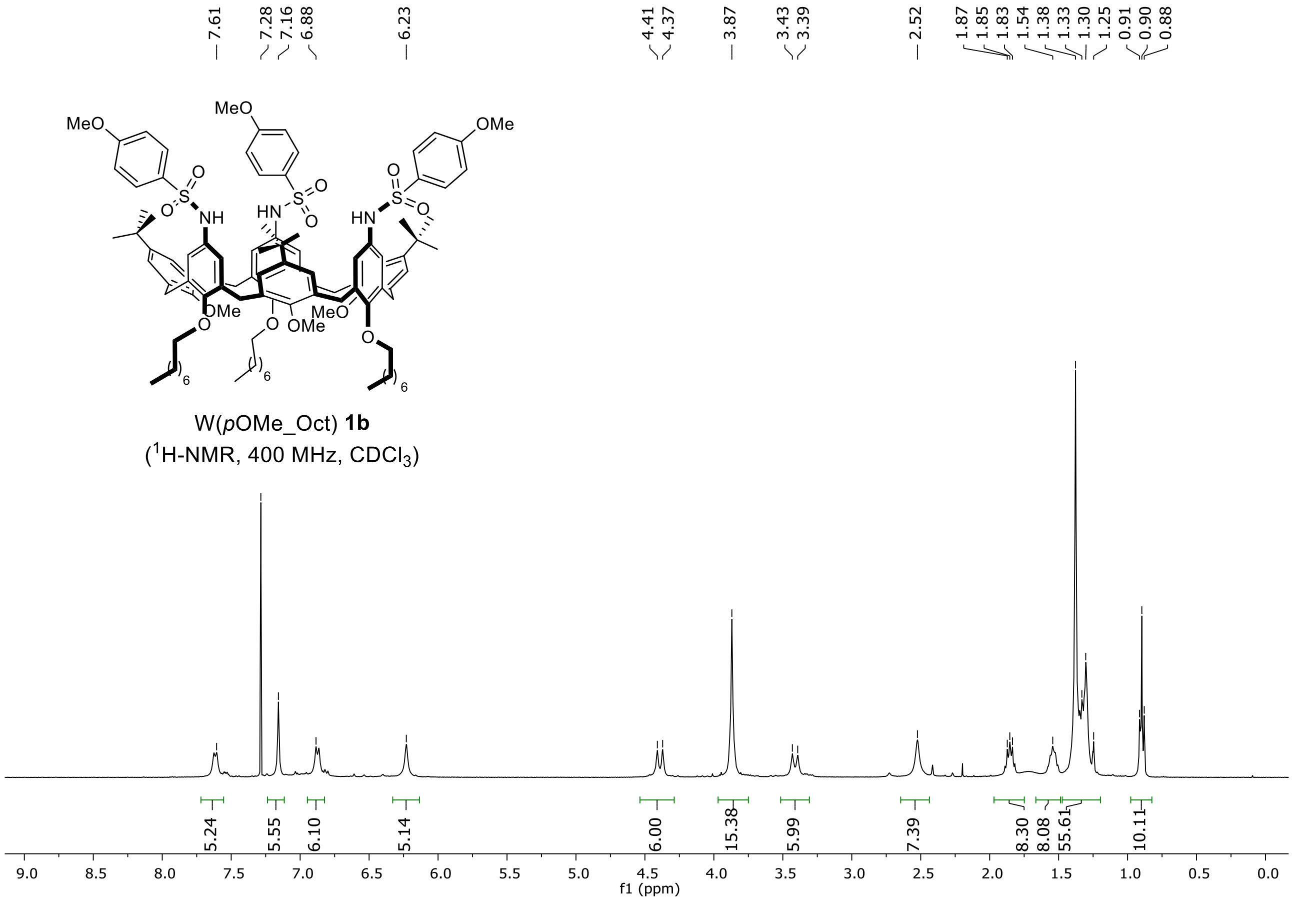
7.61
7.28
7.16
6.88
6.23
4.41
4.37
3.87
3.43
3.39
2.52
1.87
1.85
1.83
1.54
1.38
1.33
1.30
1.25
0.91
0.90
0.88
MeO
MeO
OMe
NH
HN
HN
OMe
OMe
MeO
6
6
6
W(pOMe_Oct) 1b
(1H-NMR, 400 MHz, CDCl3)
5.24
5.55
6.10
5.14
6.00
15.38
5.99
7.39
8.30
8.08
55.61
10.11
f1 (ppm)

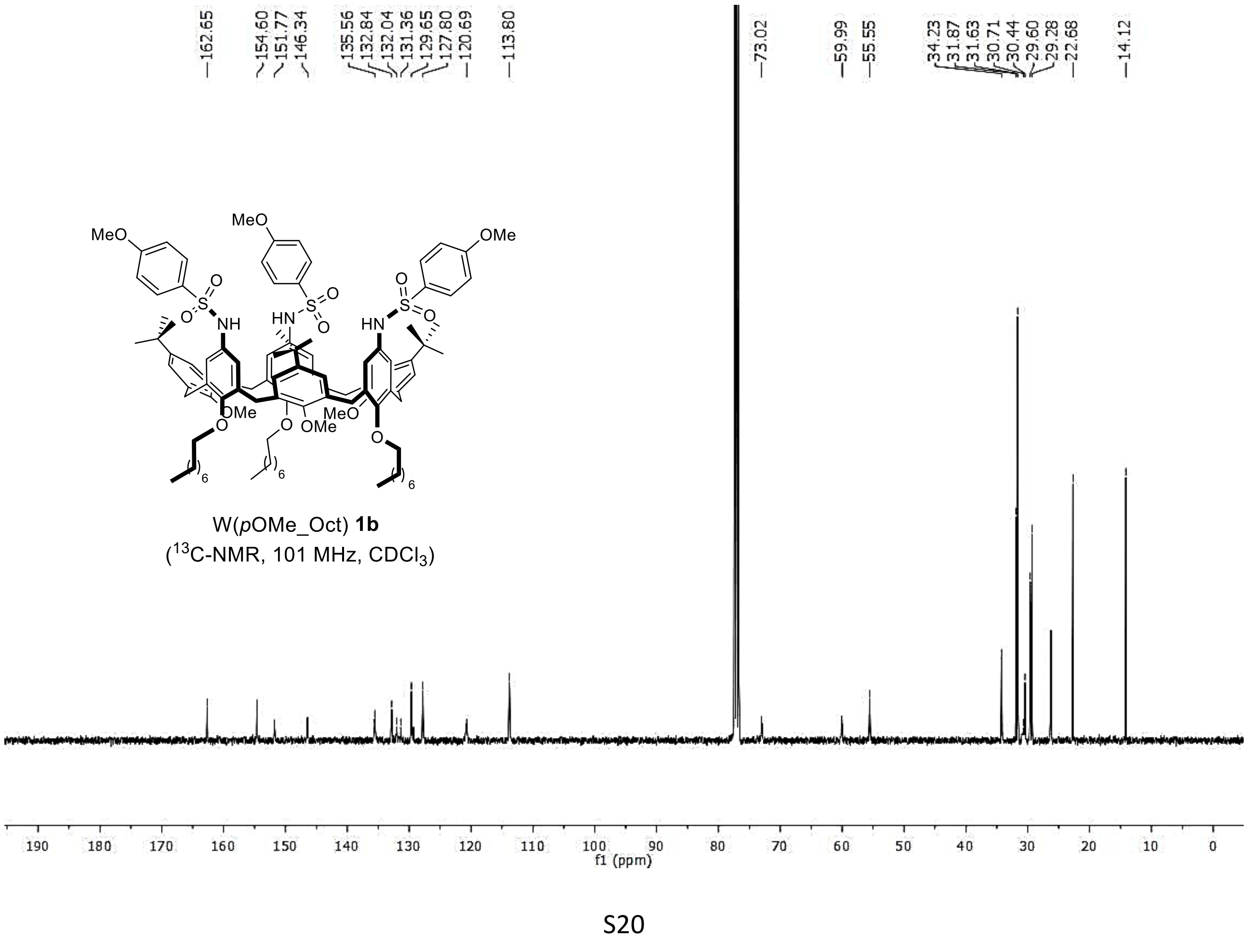
162.65
154.60
151.77
146.34
135.56
132.84
132.04
131.36
129.65
127.80
120.69
113.80
73.02
59.99
55.55
34.23
31.87
31.63
30.71
30.44
29.60
29.28
22.68
14.12
MeO
MeO
OMe
NH
HN
HN
OMe
OMe
MeO
6
6
6
W(pOMe_Oct) 1b
(13C-NMR, 101 MHz, CDCl3)
f1 (ppm)

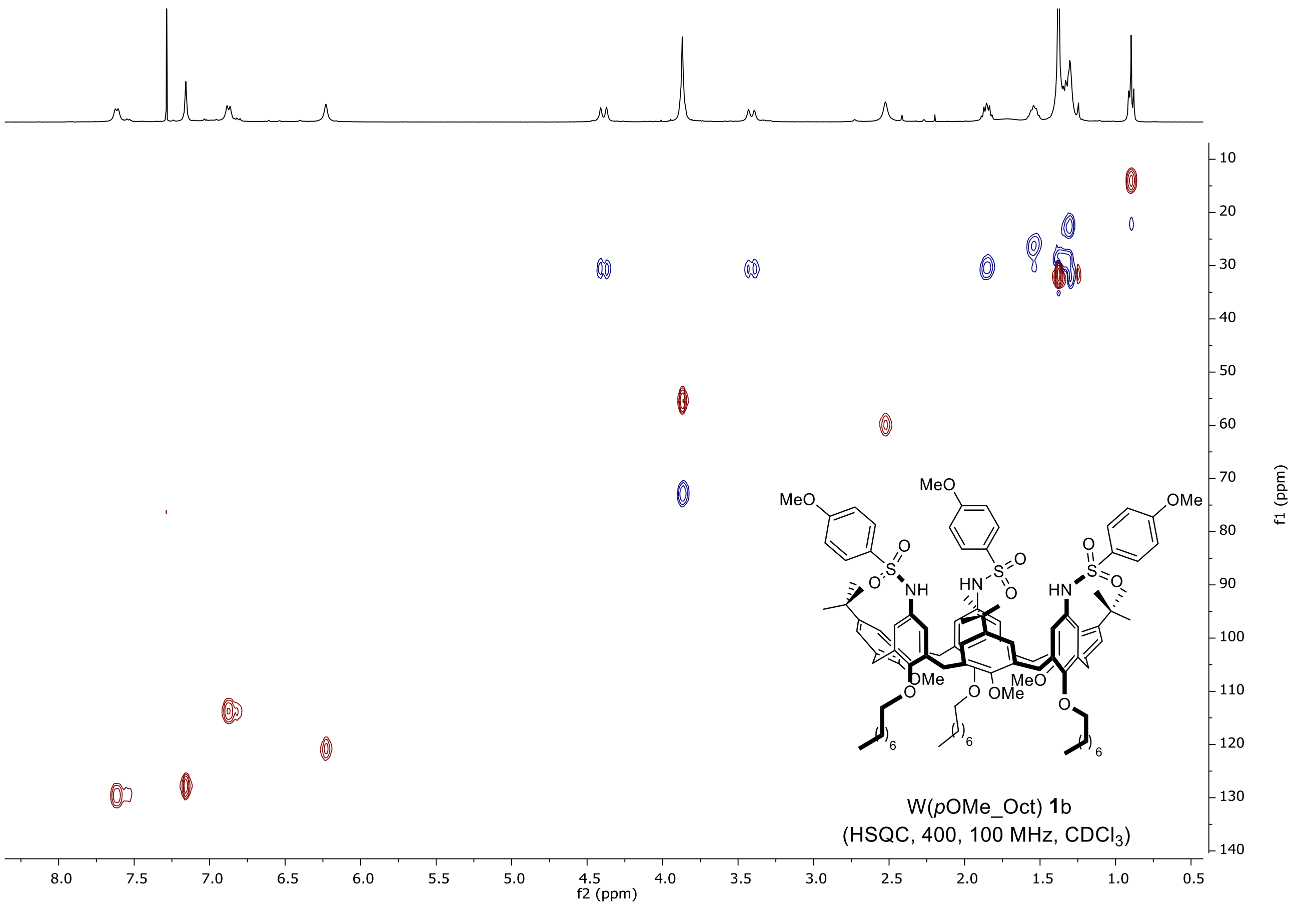
W(pOMe_Oct) 1b
(HSQC, 400, 100 MHz, CDCl3)
f2 (ppm)
f1 (ppm)

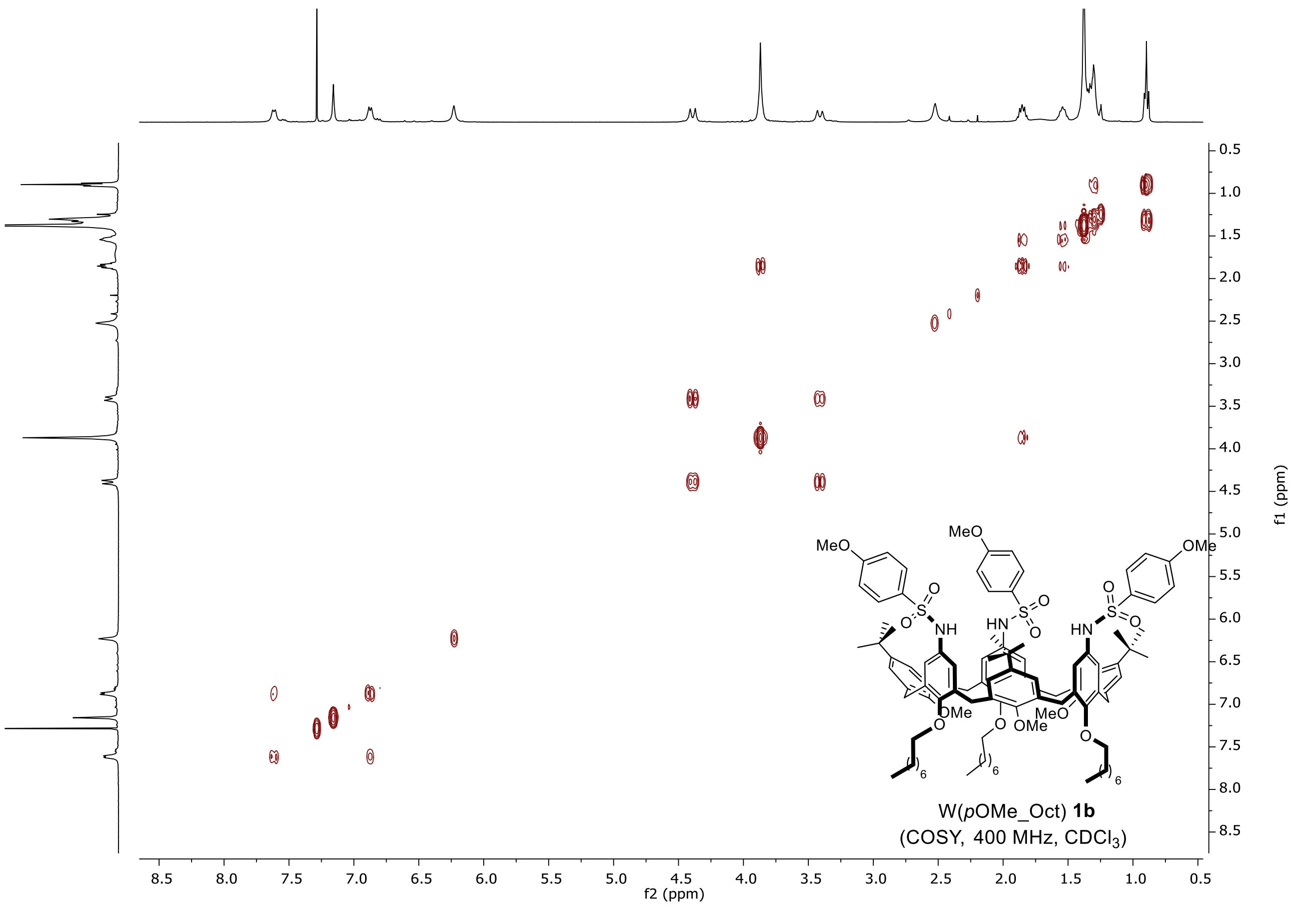
W(pOMe_Oct) 1b
(COSY, 400 MHz, CDCl3)
f2 (ppm)
f1 (ppm)

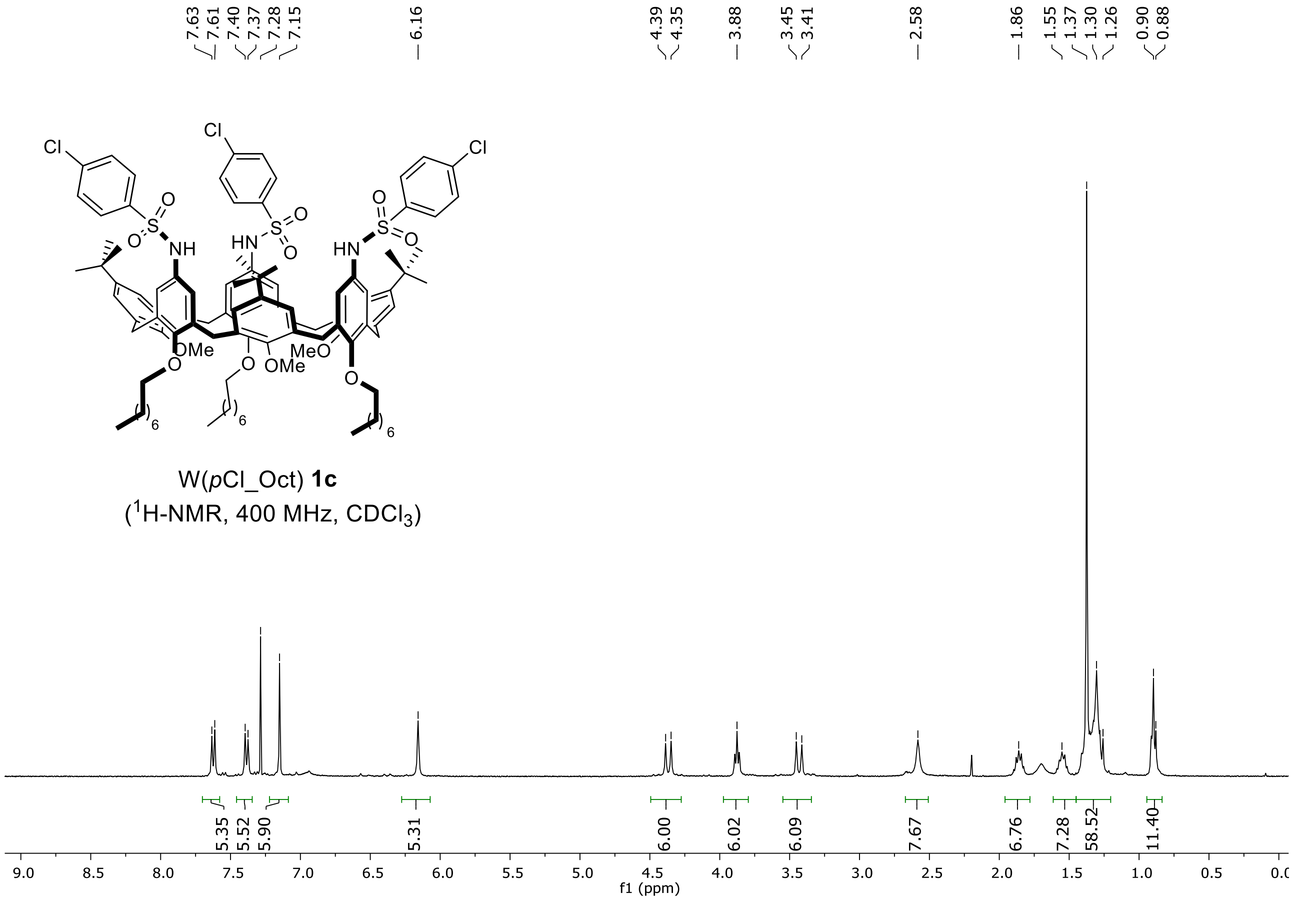
7.63
7.61
7.40
7.37
7.28
7.15
6.16
4.39
4.35
3.88
3.45
3.41
2.58
1.86
1.55
1.37
1.30
1.26
0.90
0.88
W(pCl_Oct) 1c
($^1$H-NMR, 400 MHz, CDCl$_3$)
5.35
5.52
5.90
5.31
6.00
6.02
6.09
7.67
6.76
7.28
58.52
11.40
f1 (ppm)

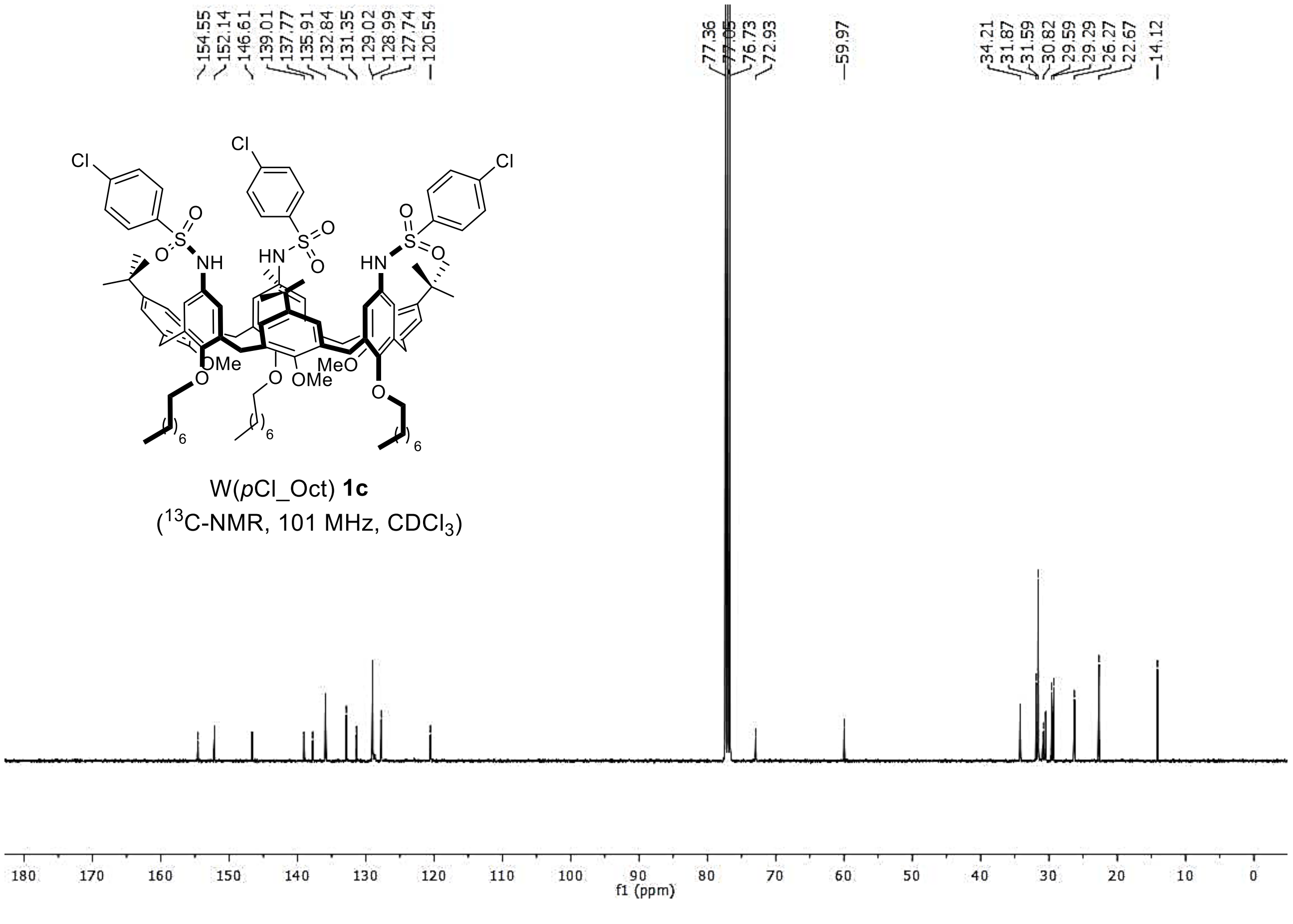
154.55
152.14
146.61
139.01
137.77
135.91
132.84
131.35
129.02
128.99
127.74
120.54
77.36
77.05
76.73
72.93
59.97
34.21
31.87
31.59
30.82
29.59
29.29
26.27
22.67
14.12
W(pCl_Oct) 1c
($^{13}$C-NMR, 101 MHz, CDCl$_3$)
f1 (ppm)

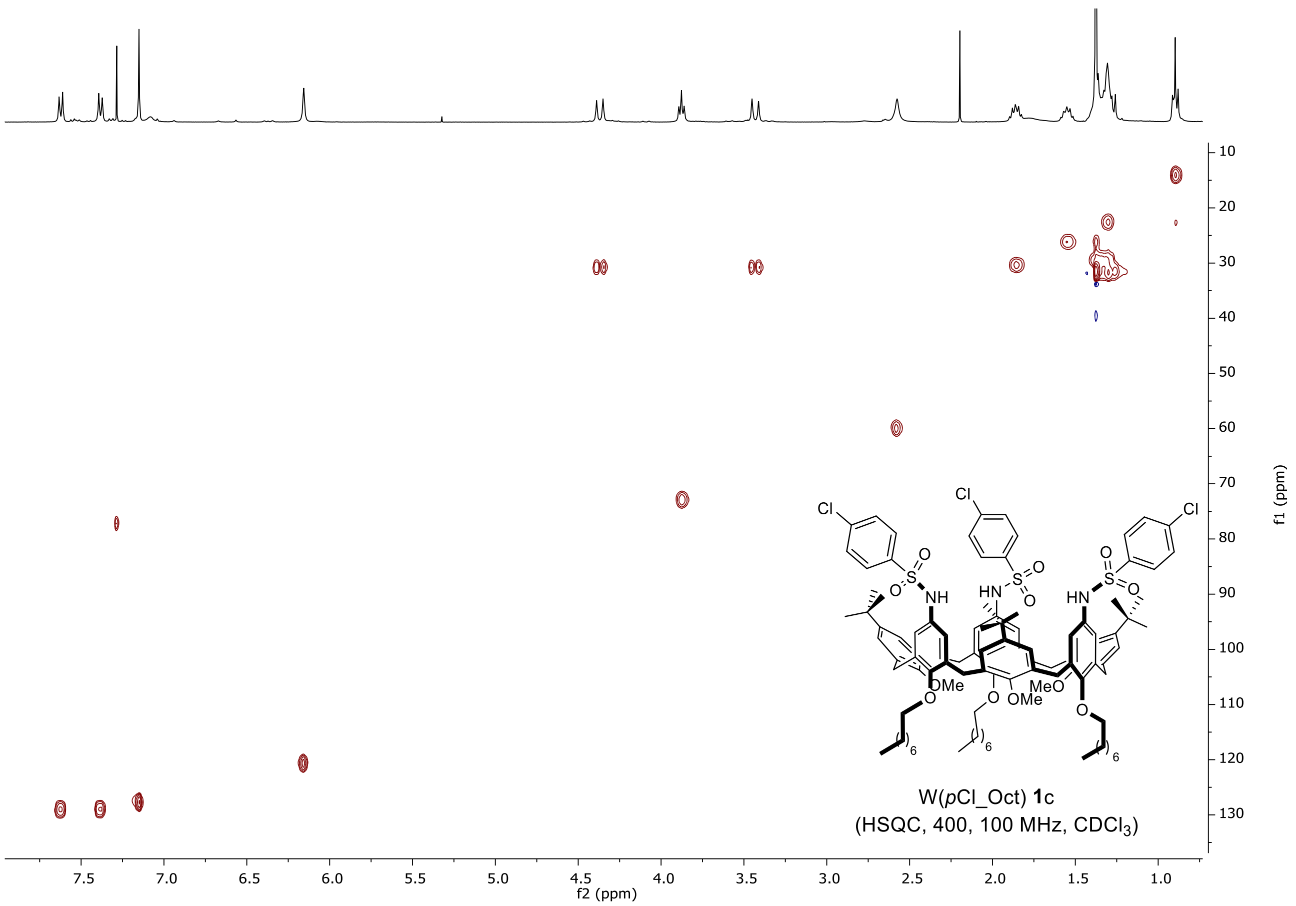
W(pCl_Oct) 1c
(HSQC, 400, 100 MHz, CDCl3)
f2 (ppm)
f1 (ppm)

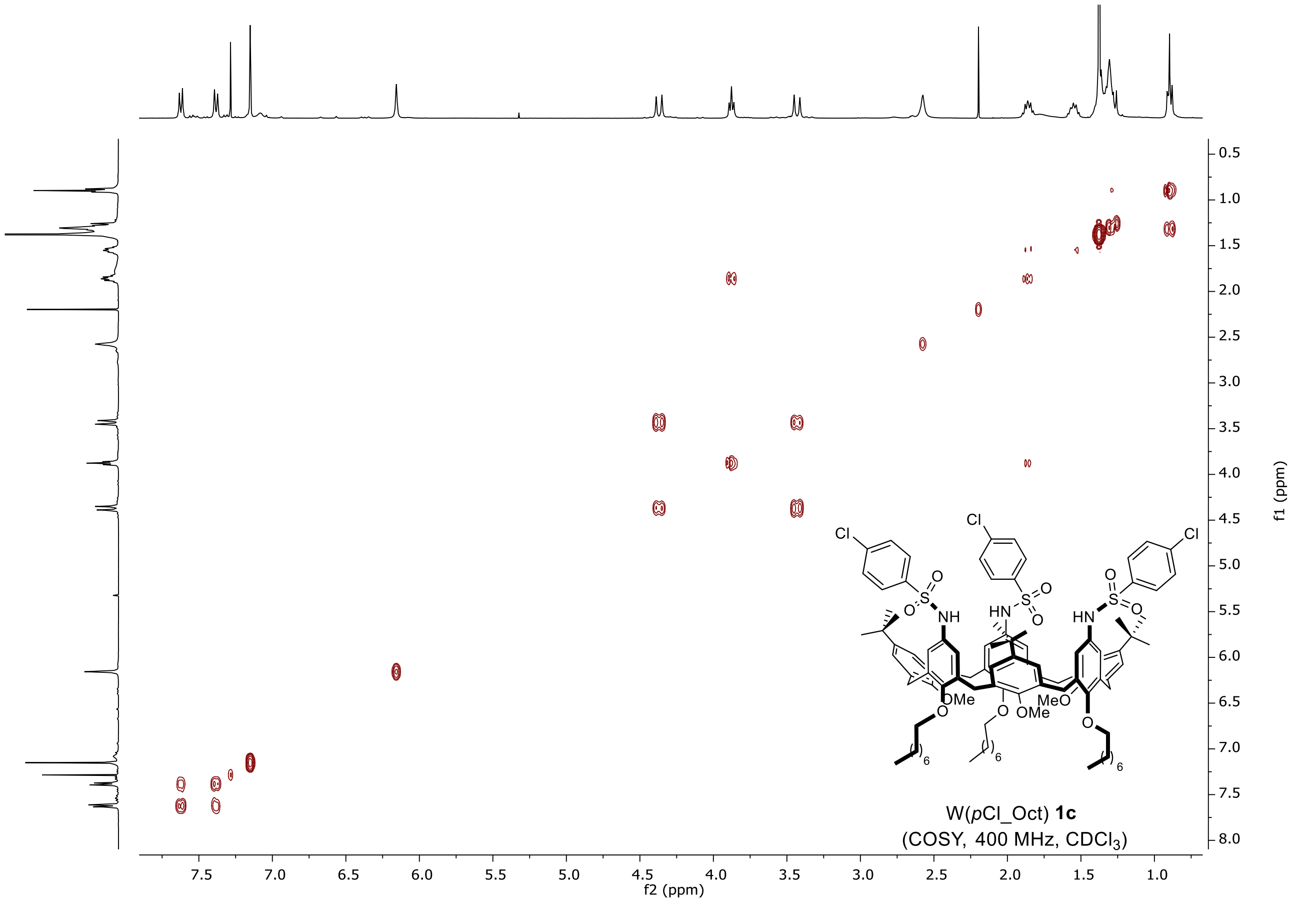
W(pCl_Oct) 1c
(COSY, 400 MHz, CDCl3)
f2 (ppm)
f1 (ppm)

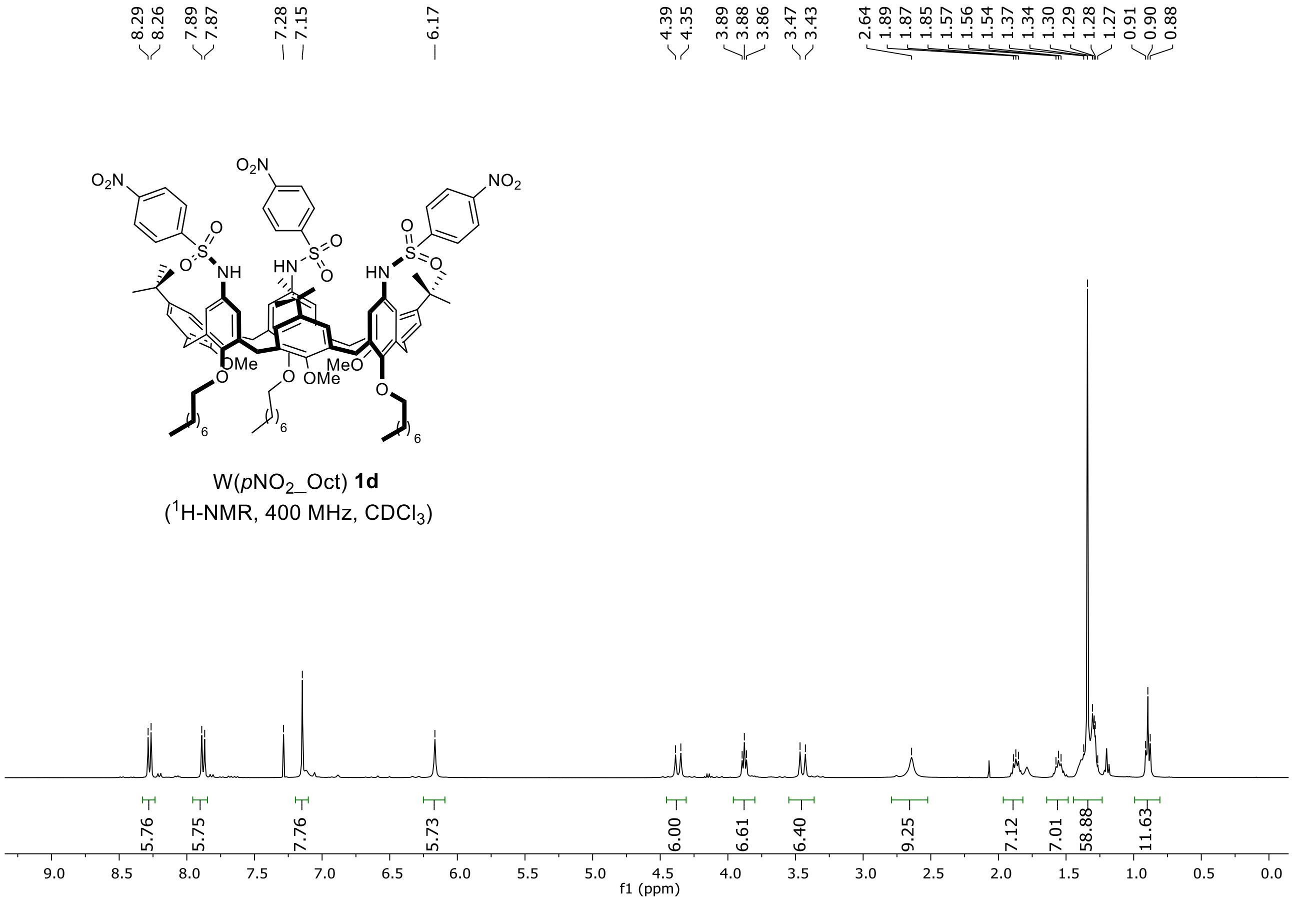


W(*p*NO$_2$_Oct) **1d**
($^1$H-NMR, 400 MHz, CDCl$_3$)

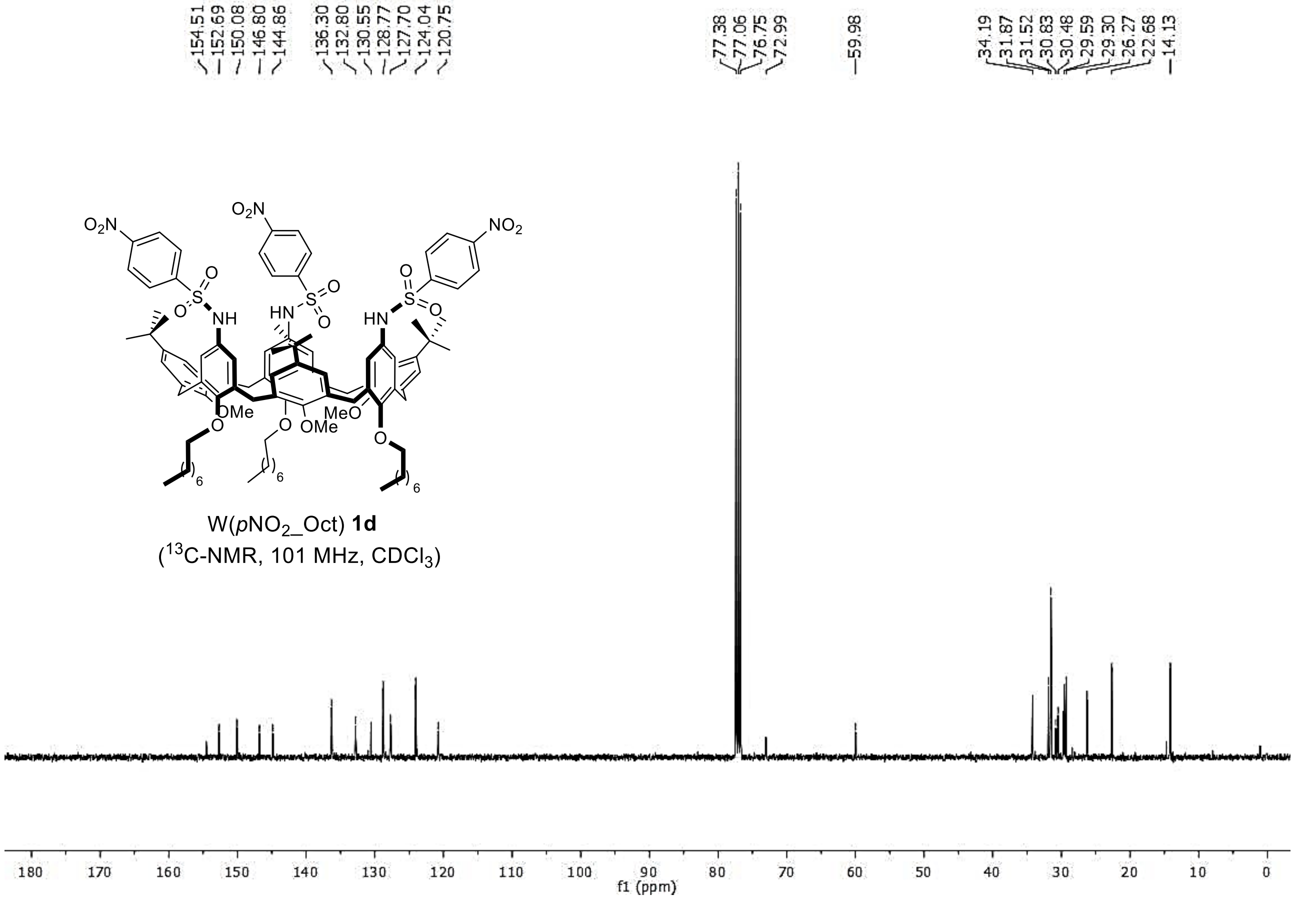


W(*p*NO$_2$_Oct) **1d**
($^{13}$C-NMR, 101 MHz, CDCl$_3$)

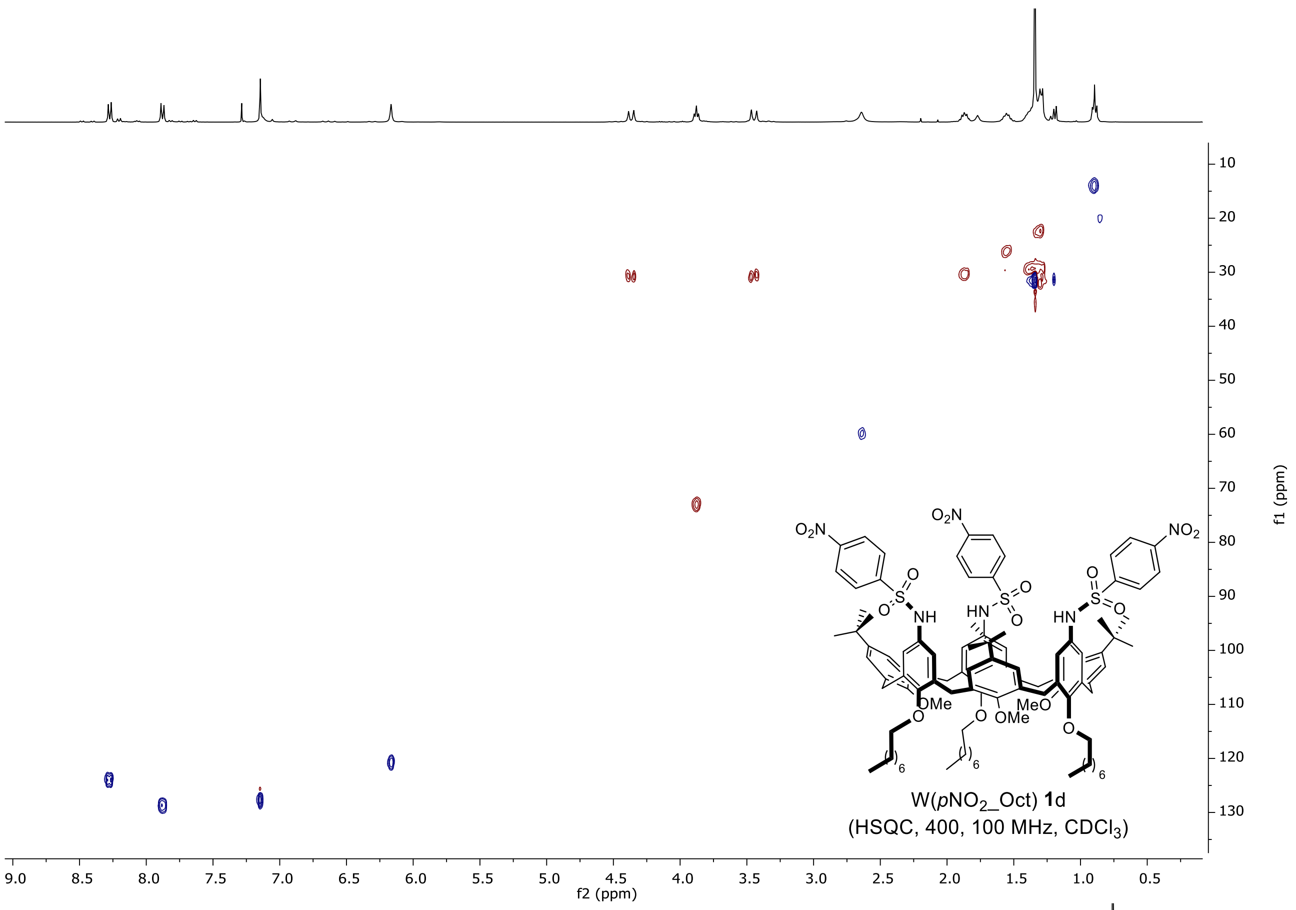

f1 (ppm)
f2 (ppm)
$O_2N$
$NO_2$
NH
HN
OMe
MeO
W(pNO2_Oct) 1d
(HSQC, 400, 100 MHz, $CDCl_3$)


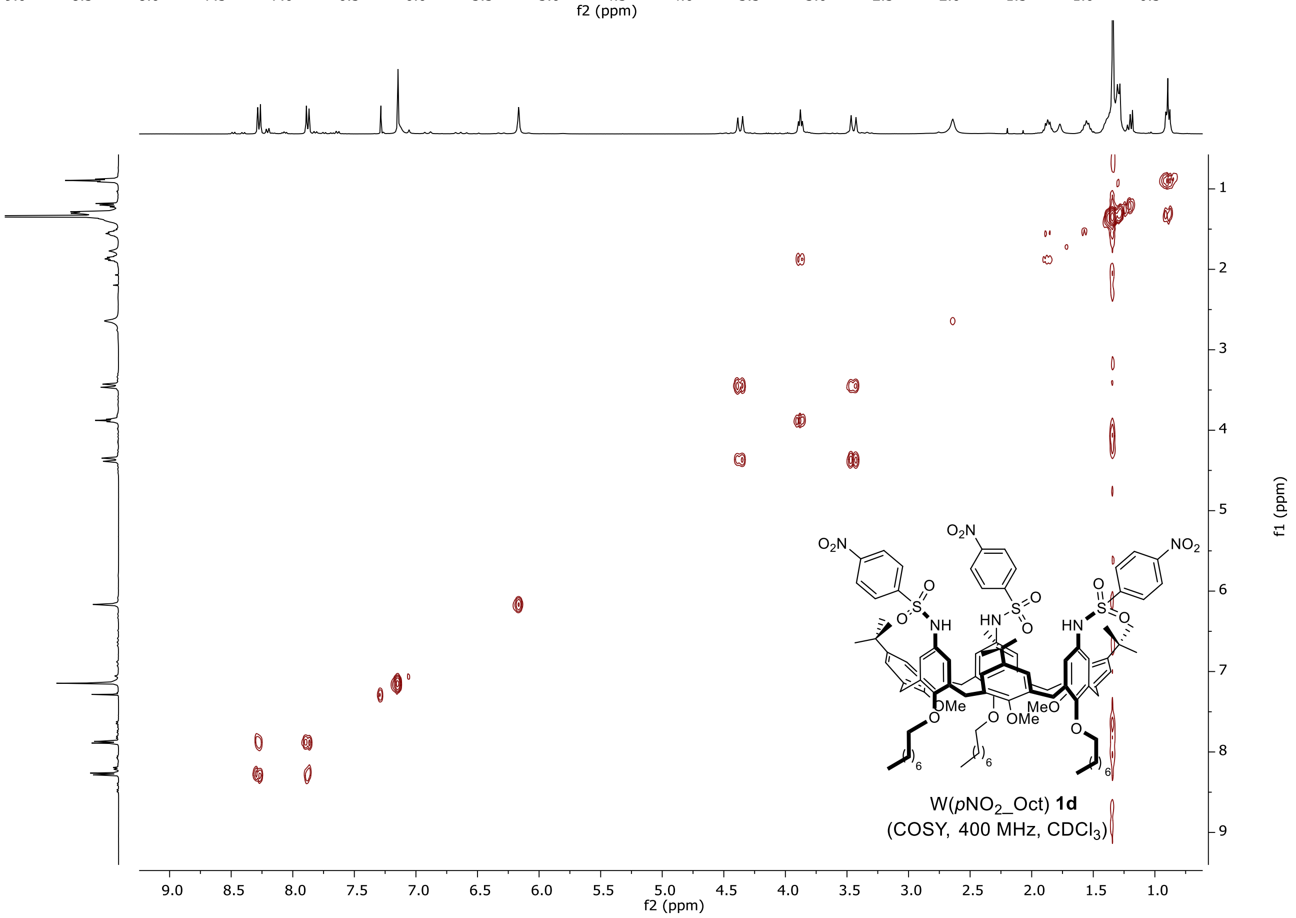

f1 (ppm)
f2 (ppm)
$O_2N$
$NO_2$
NH
HN
OMe
MeO
W(pNO2_Oct) 1d
(COSY, 400 MHz, $CDCl_3$)

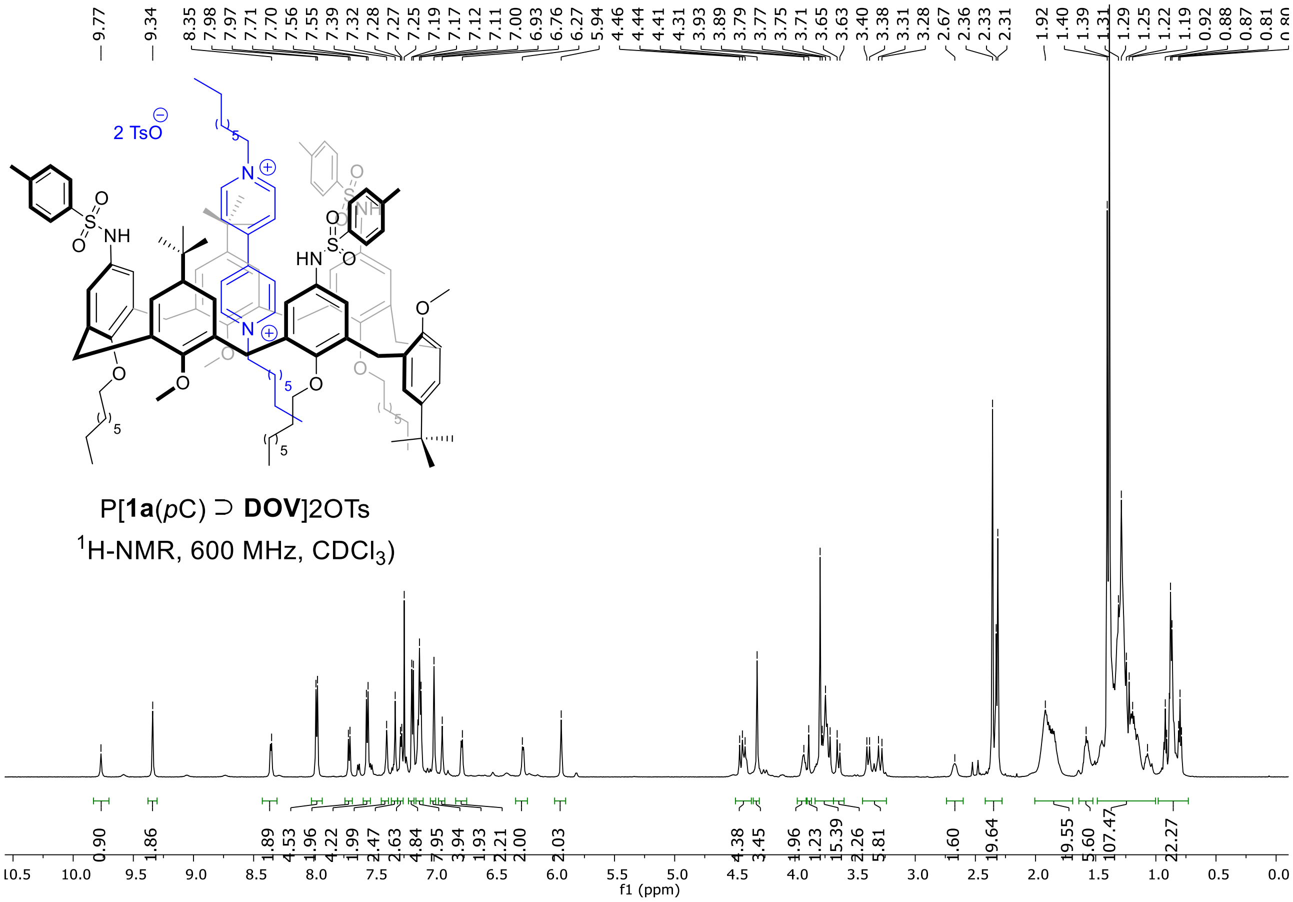
2 TsO
P[1a(pC) ⊃ DOV]2OTs
1H-NMR, 600 MHz, CDCl3)
f1 (ppm)

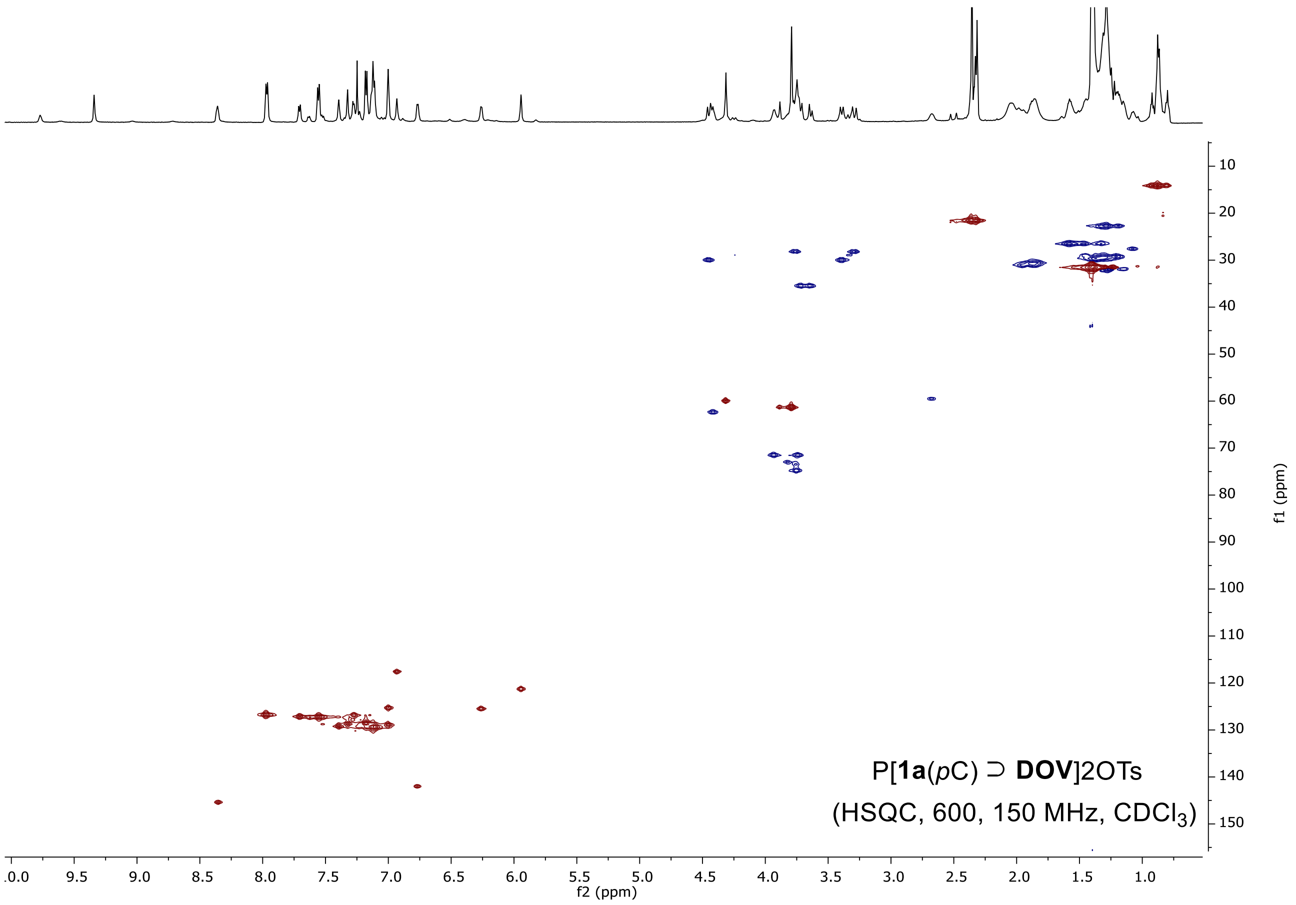
P[1a(pC) ⊃ DOV]2OTs
(HSQC, 600, 150 MHz, CDCl3)
f2 (ppm)
f1 (ppm)

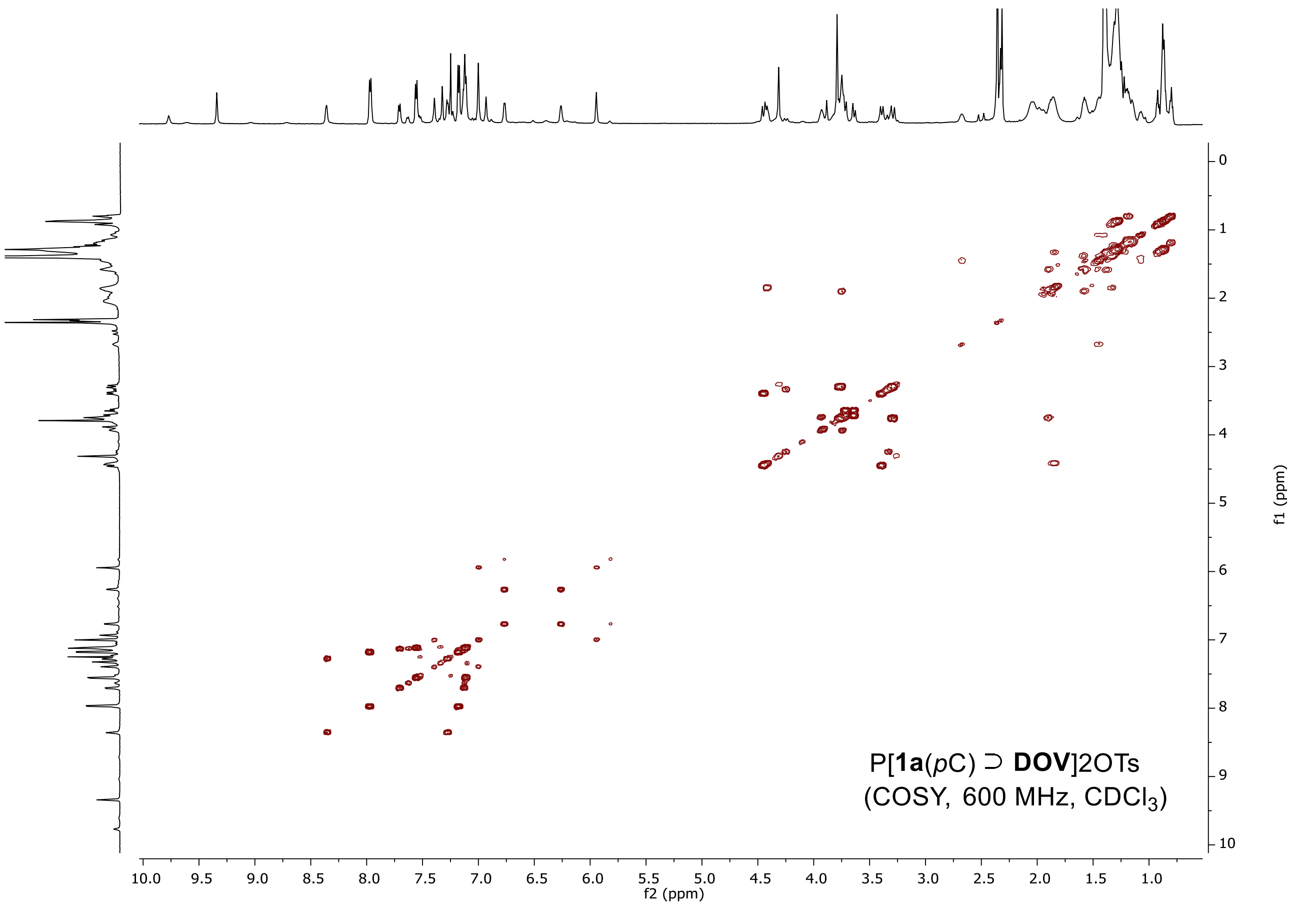

P[**1a**(*p*C) ⊃ **DOV**]2OTs
(COSY, 600 MHz, $CDCl_3$)
f1 (ppm)
f2 (ppm)


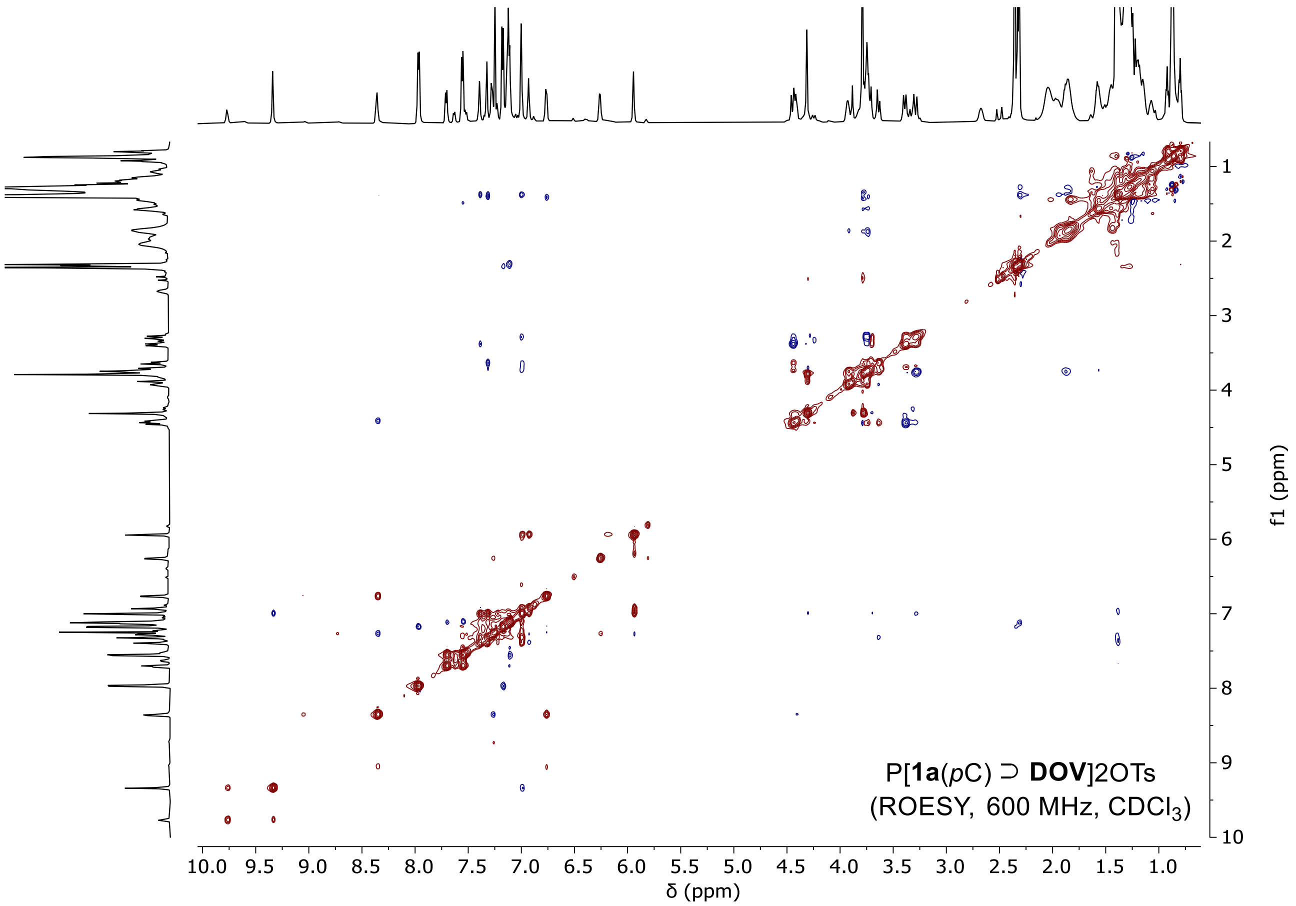

P[**1a**(*p*C) ⊃ **DOV**]2OTs
(ROESY, 600 MHz, $CDCl_3$)
f1 (ppm)
δ (ppm)

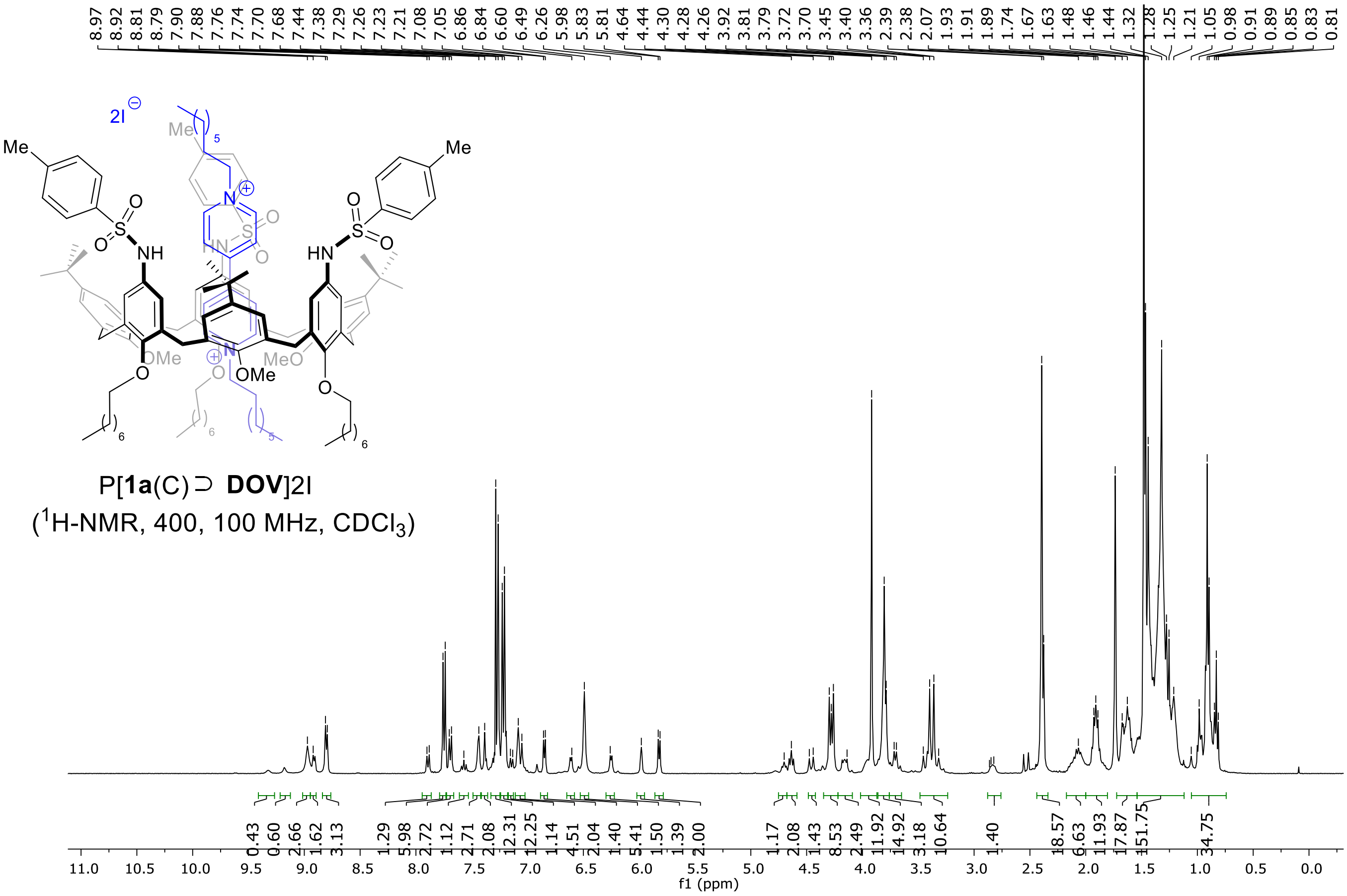


P[**1a**(C)⊃ **DOV**]2I
($^{1}$H-NMR, 400, 100 MHz, $CDCl_3$)



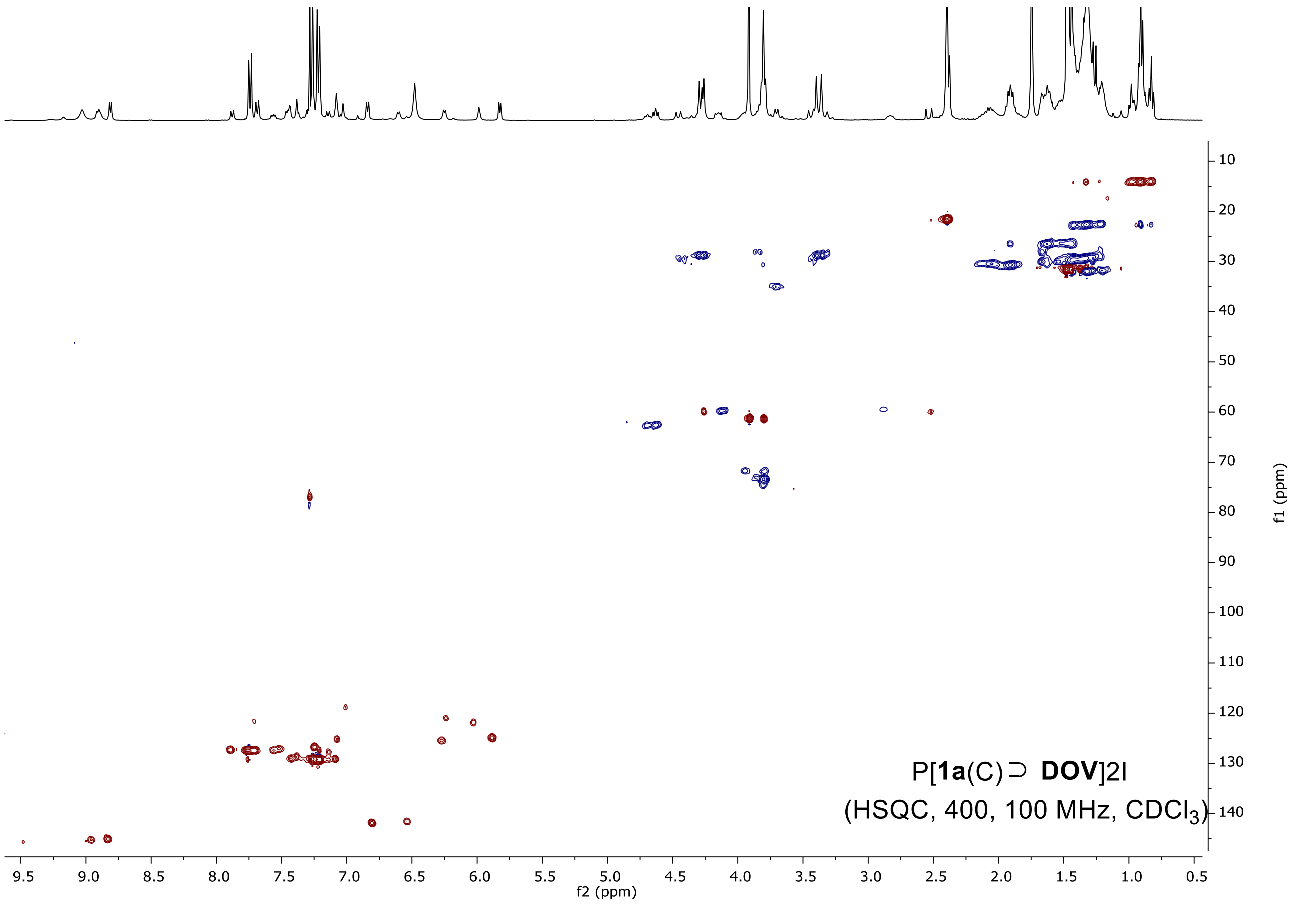


P[**1a**(C)⊃ **DOV**]2I
(HSQC, 400, 100 MHz, $CDCl_3$)

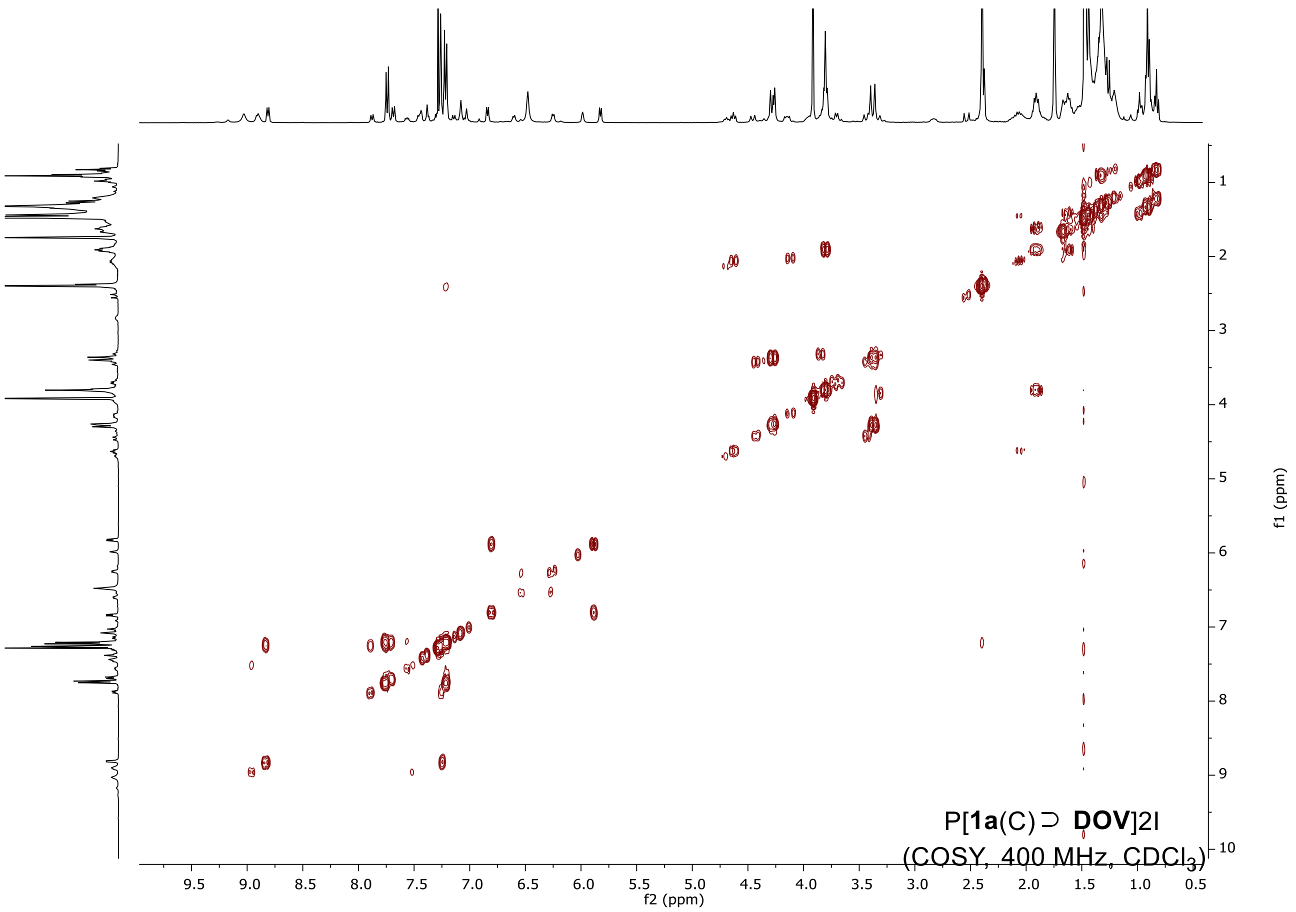
P[1a(C)⊃ DOV]2I
(COSY, 400 MHz, CDCl3)
f2 (ppm)
f1 (ppm)

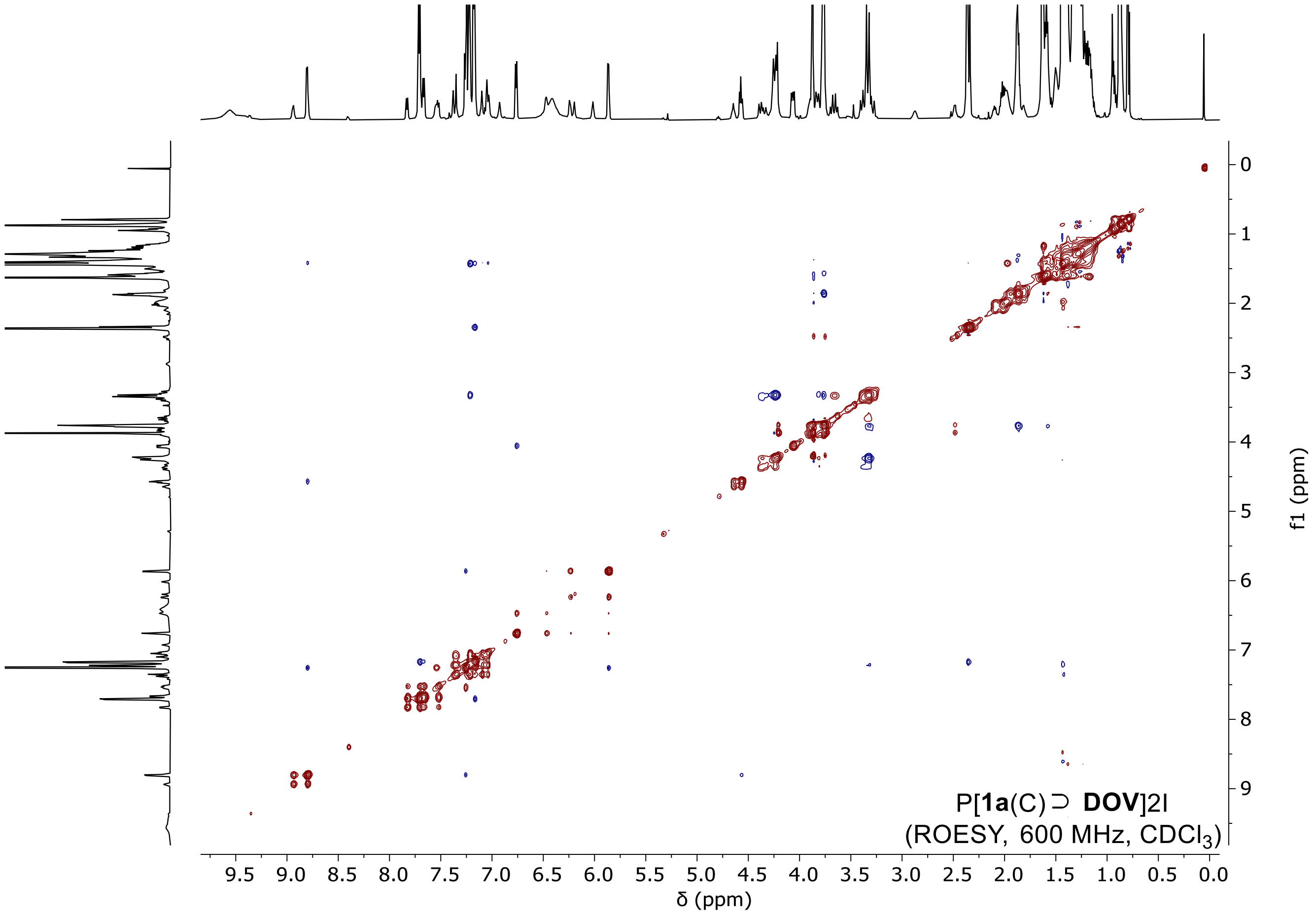
P[1a(C)⊃ DOV]2I
(ROESY, 600 MHz, CDCl3)
δ (ppm)
f1 (ppm)

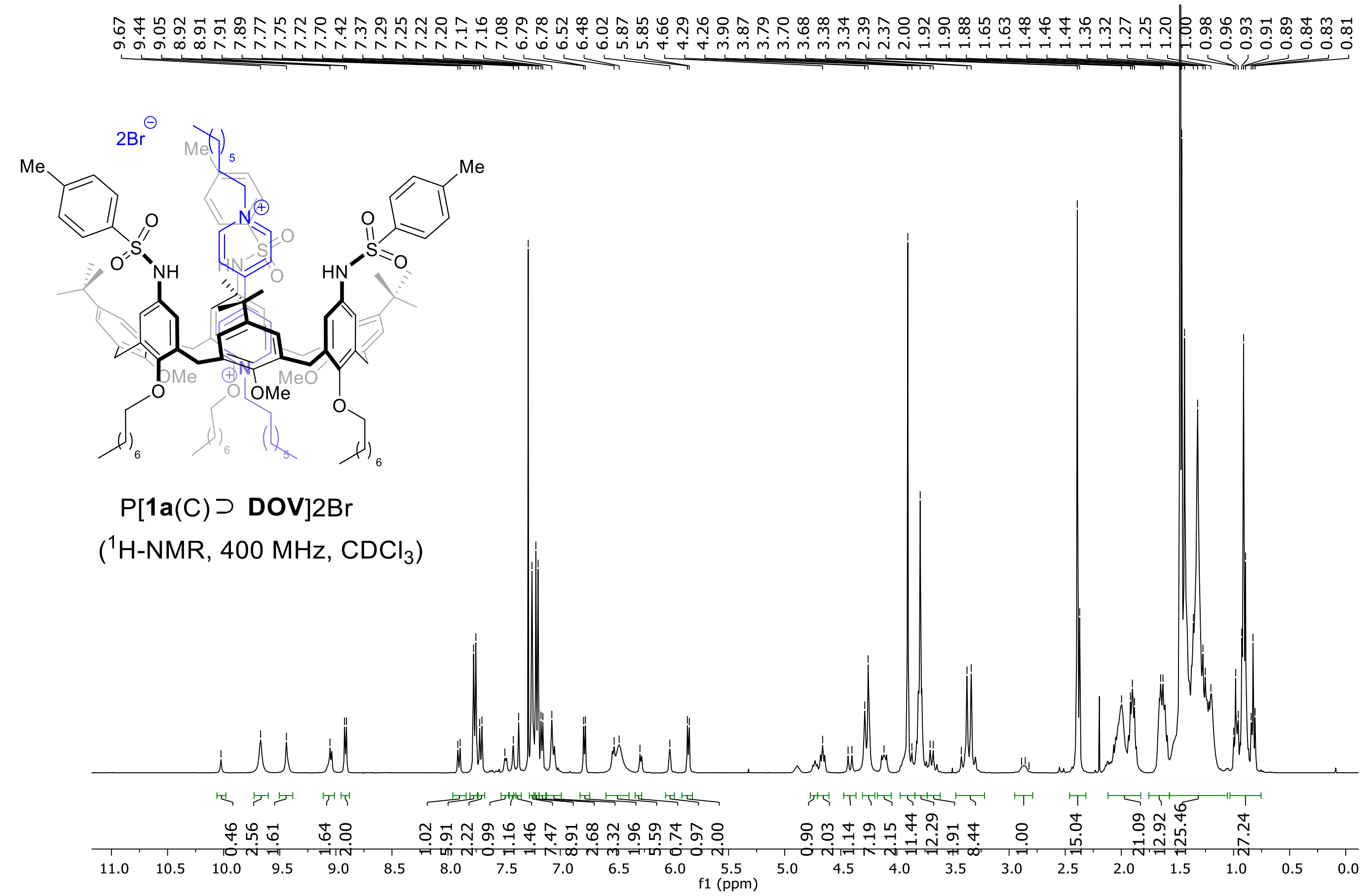
P[1a(C)⊃ DOV]2Br
(1H-NMR, 400 MHz, CDCl3)
f1 (ppm)

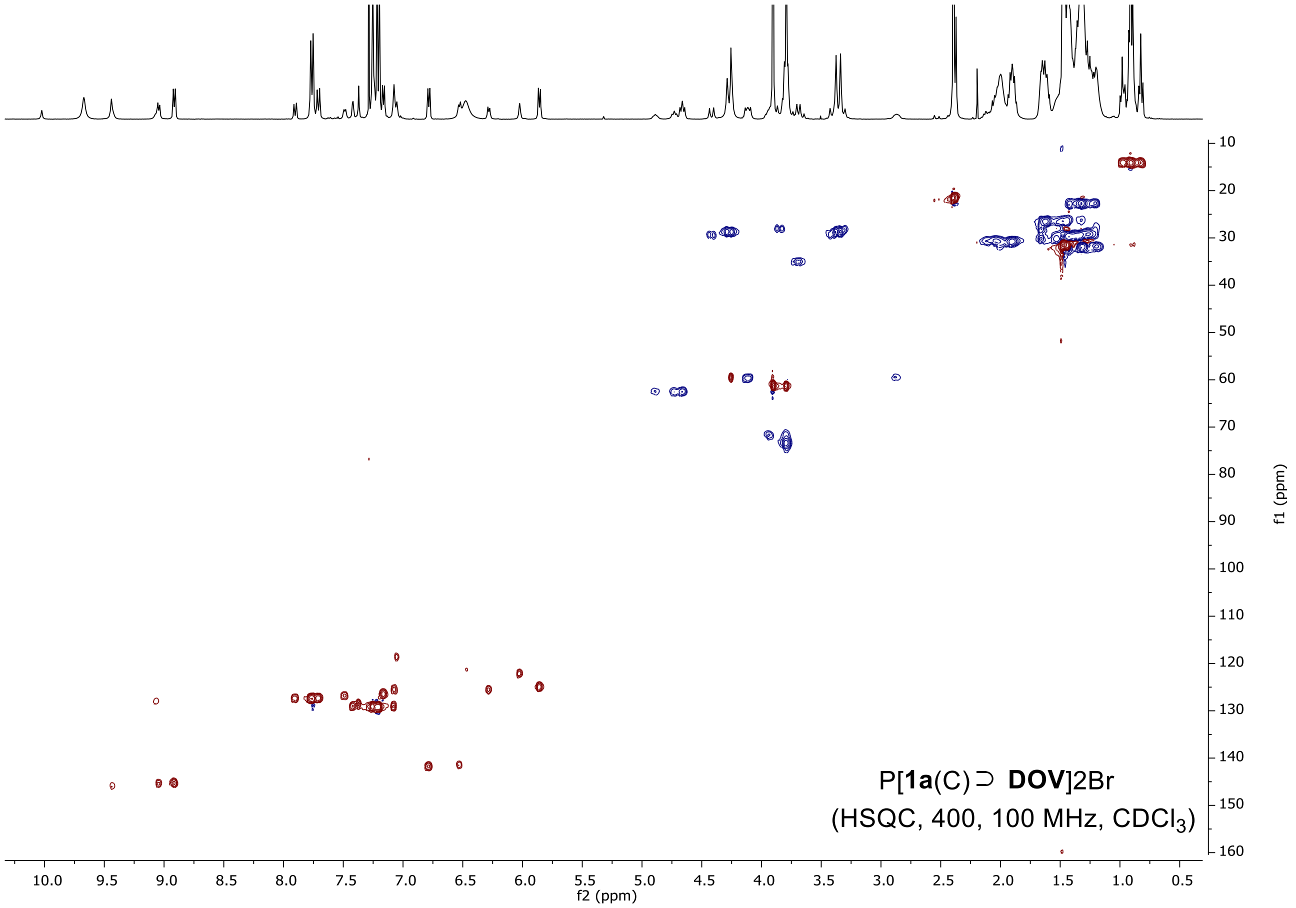
P[1a(C)⊃ DOV]2Br
(HSQC, 400, 100 MHz, CDCl3)
f2 (ppm)
f1 (ppm)

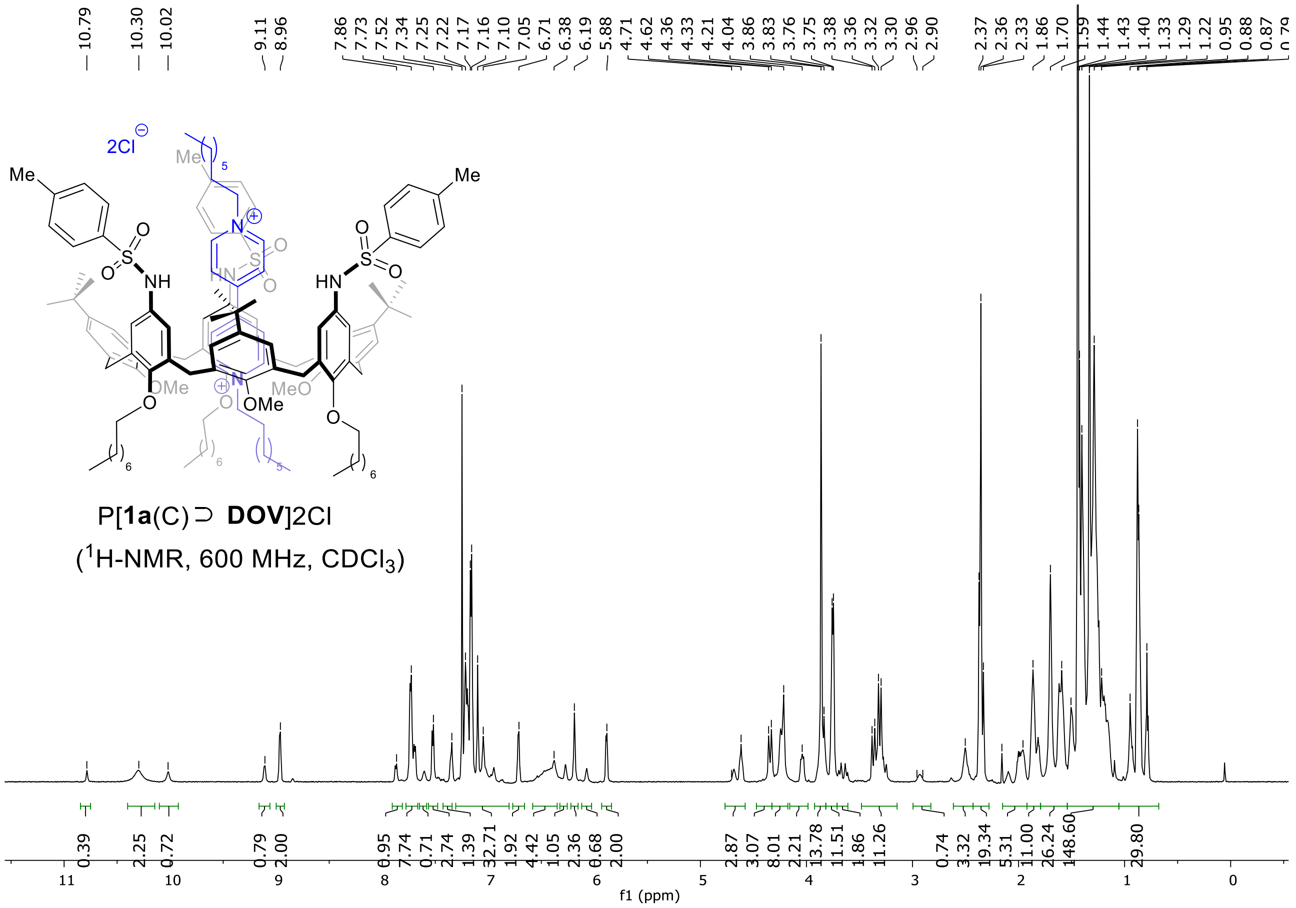

2Cl
Me
Me
NH
HN
OMe
MeO
OMe
P[1a(C)⊃ DOV]2Cl
($^1$H-NMR, 600 MHz, CDCl$_3$)
f1 (ppm)


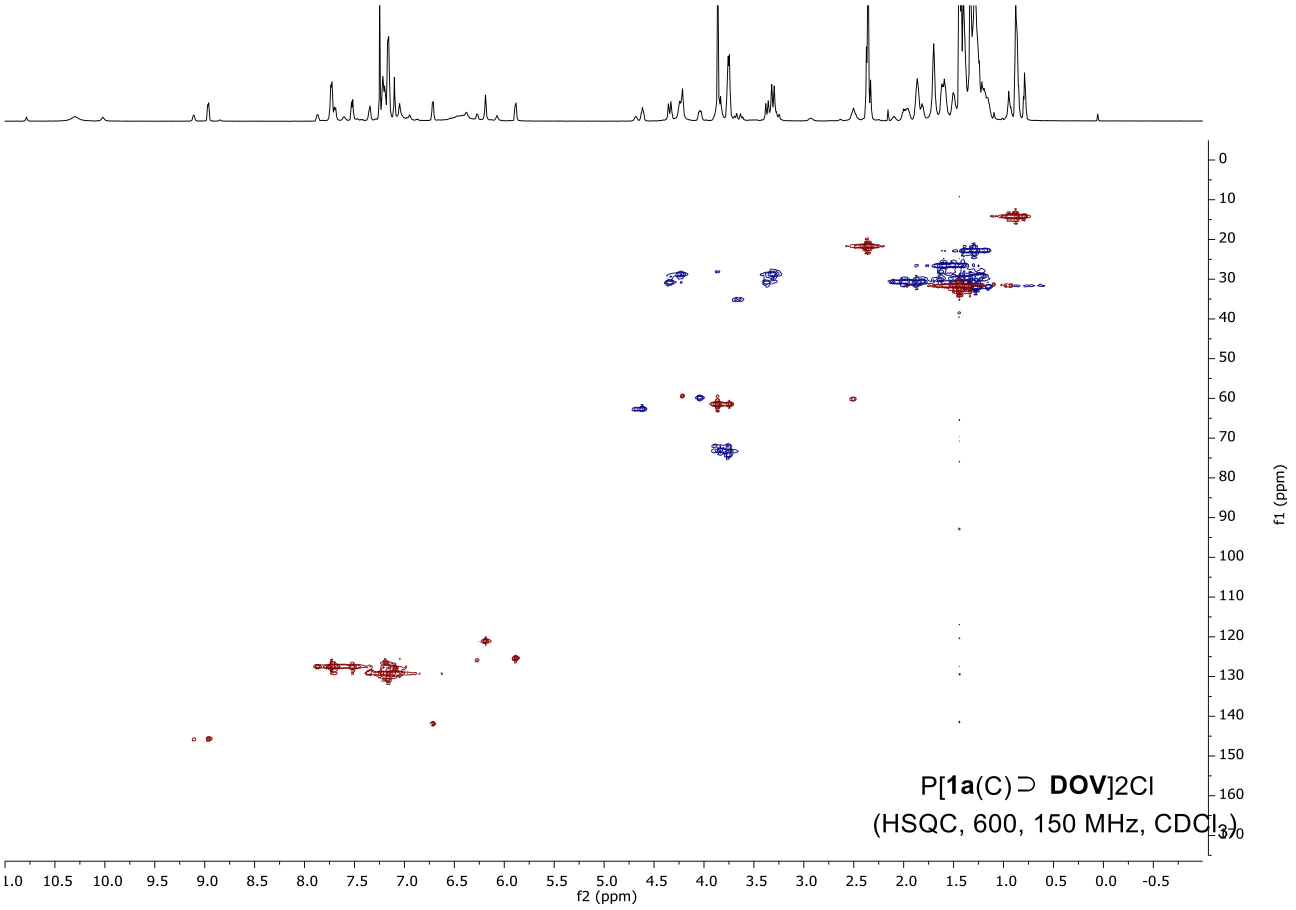

P[1a(C)⊃ DOV]2Cl
(HSQC, 600, 150 MHz, CDCl$_3$)
f2 (ppm)
f1 (ppm)

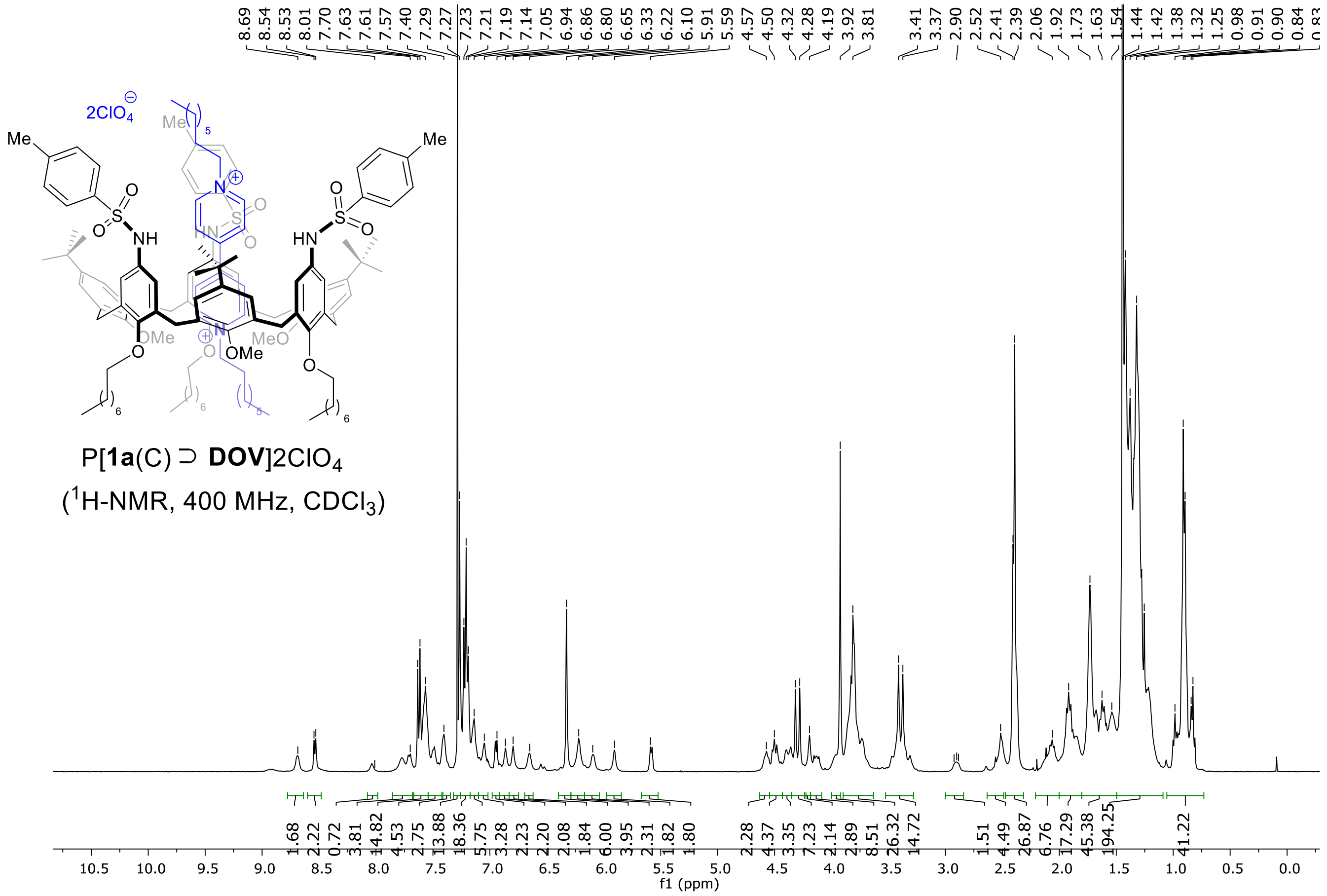

P[1a(C) ⊃ DOV]2ClO4
(1H-NMR, 400 MHz, CDCl3)
f1 (ppm)


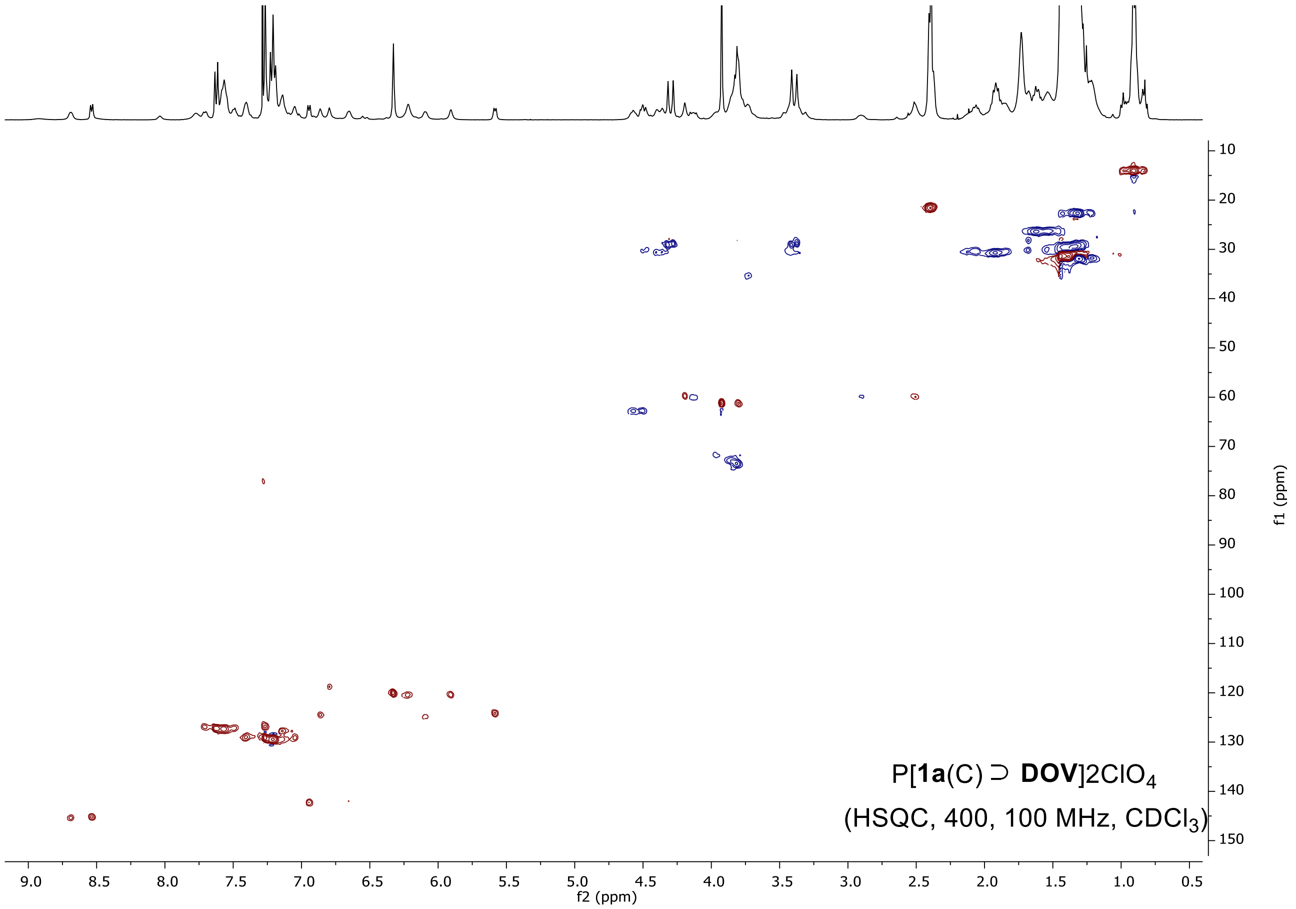

P[1a(C) ⊃ DOV]2ClO4
(HSQC, 400, 100 MHz, CDCl3)
f2 (ppm)
f1 (ppm)

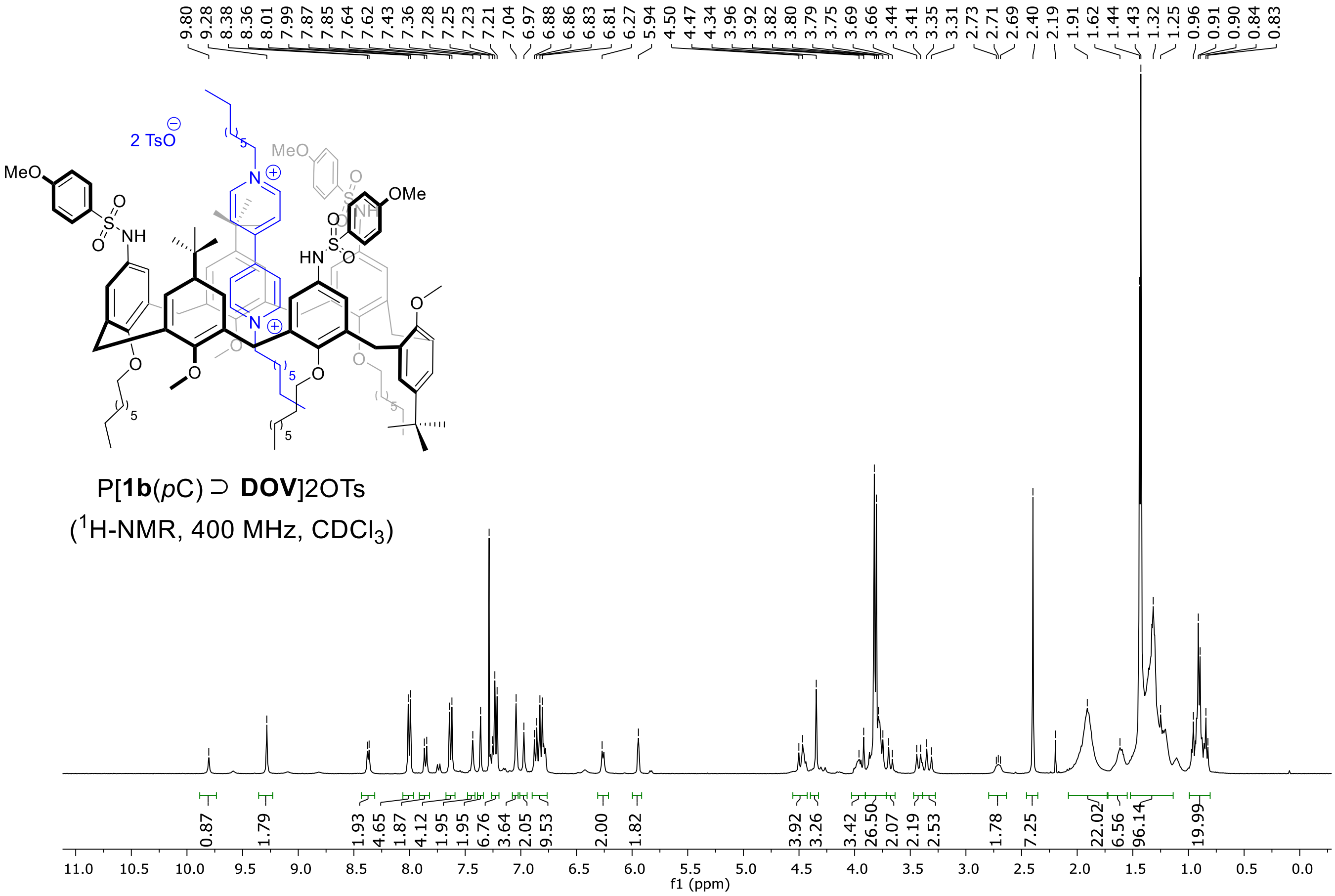

P[**1b**(*p*C) ⊃ **DOV**]2OTs
($^{1}$H-NMR, 400 MHz, $CDCl_3$)

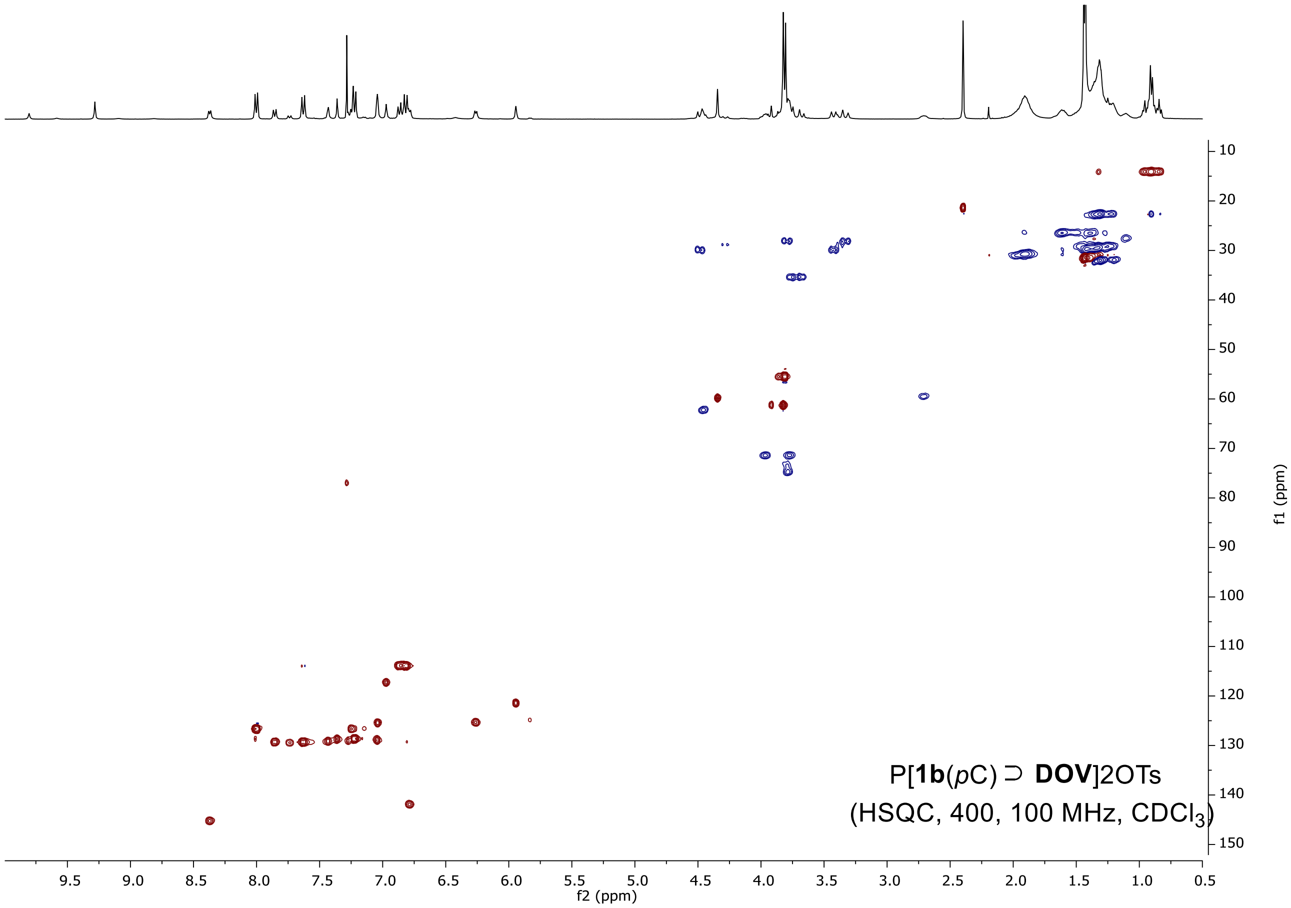

P[**1b**(*p*C) ⊃ **DOV**]2OTs
(HSQC, 400, 100 MHz, $CDCl_3$)

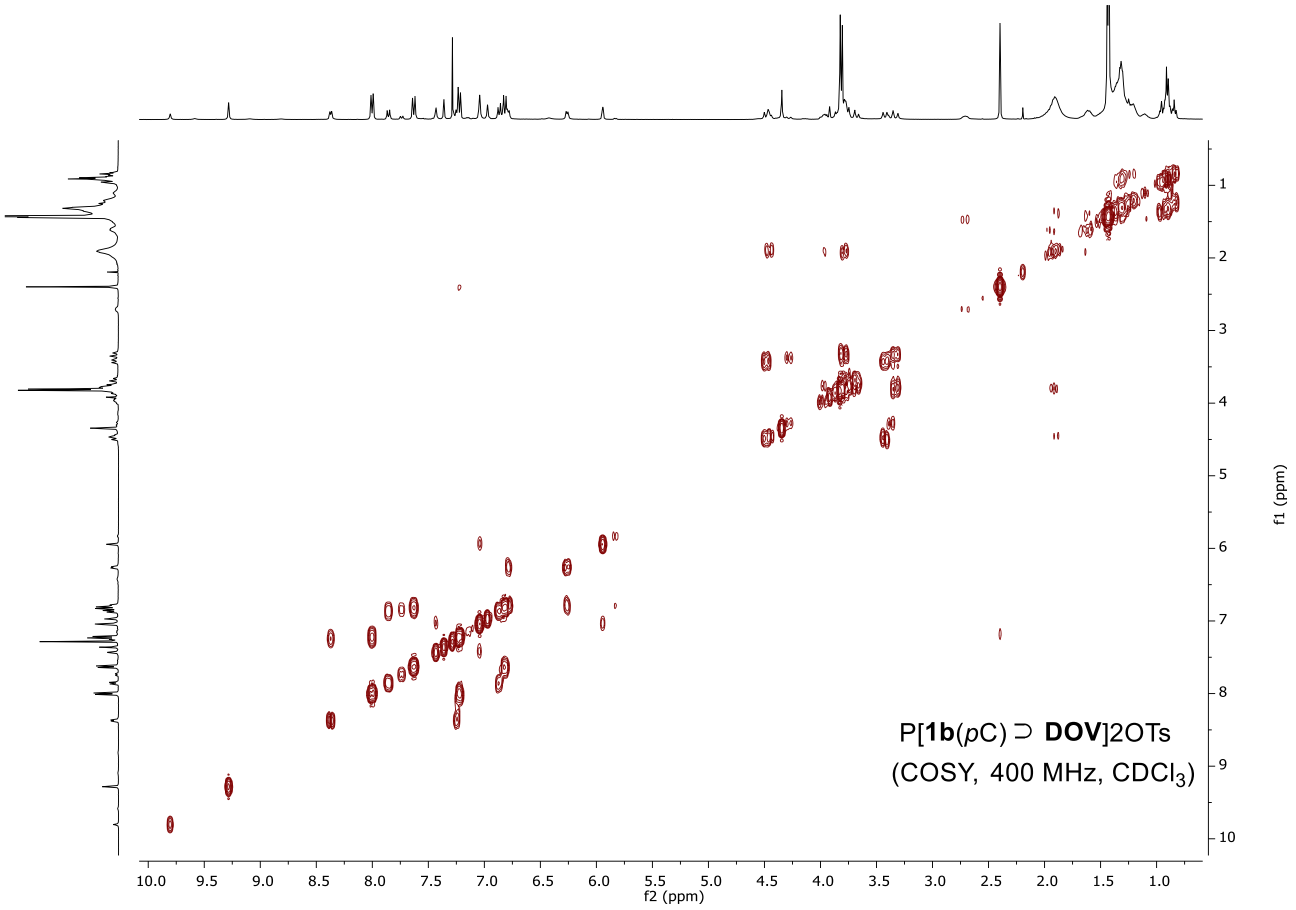
P[1b(pC) ⊃ DOV]2OTs
(COSY, 400 MHz, CDCl3)
f2 (ppm)
f1 (ppm)

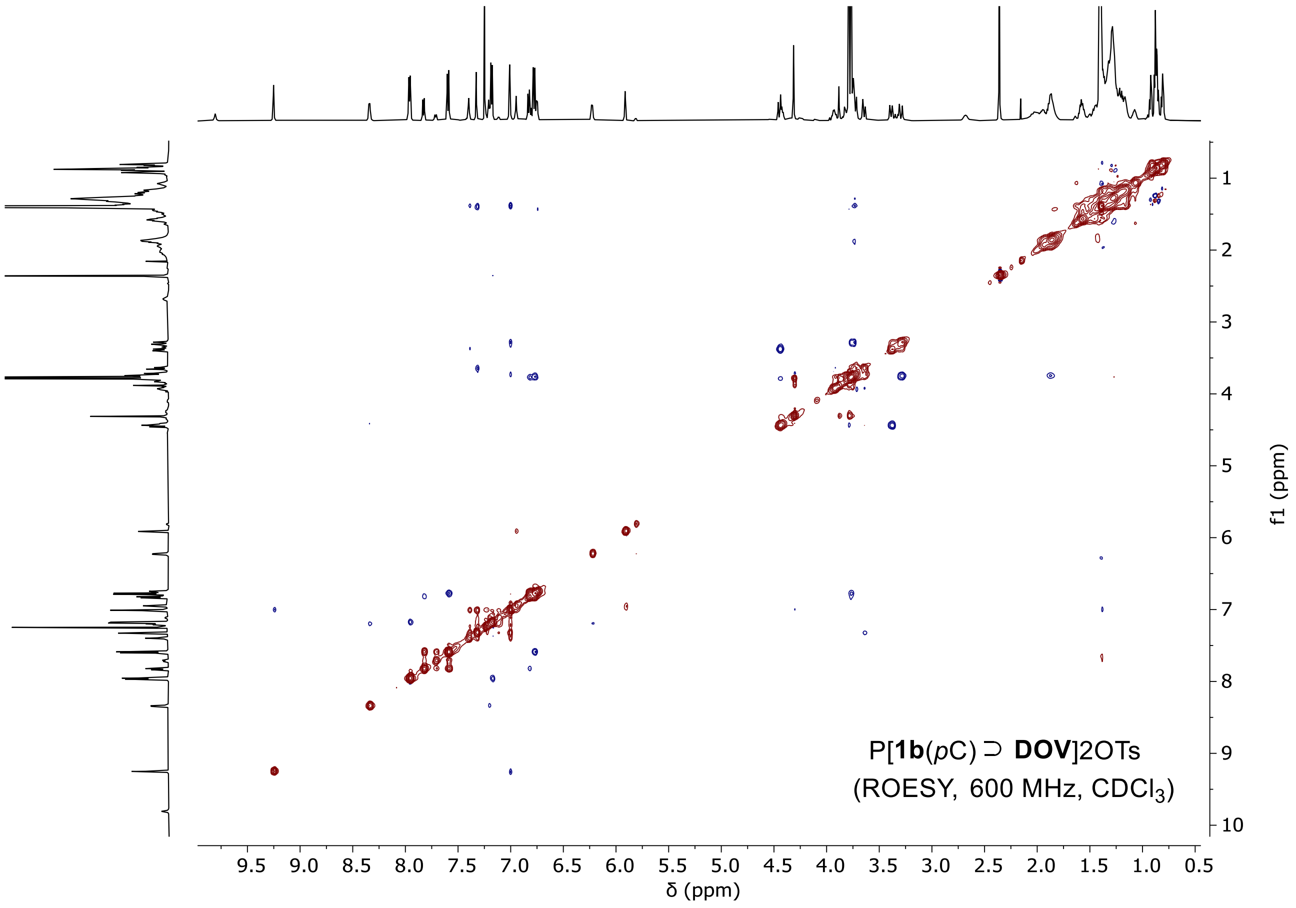
P[1b(pC) ⊃ DOV]2OTs
(ROESY, 600 MHz, CDCl3)
δ (ppm)
f1 (ppm)

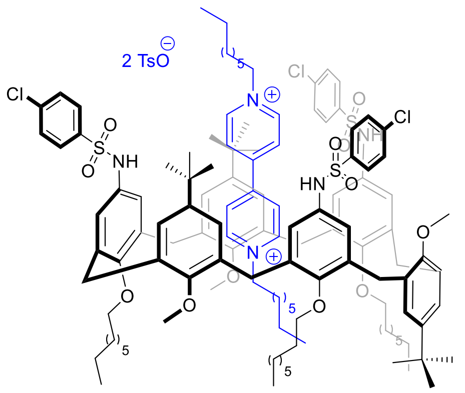


P[**1c**(*p*C) ⊃ **DOV**]2OTs
($^{1}$H-NMR, 400 MHz, $CDCl_3$)

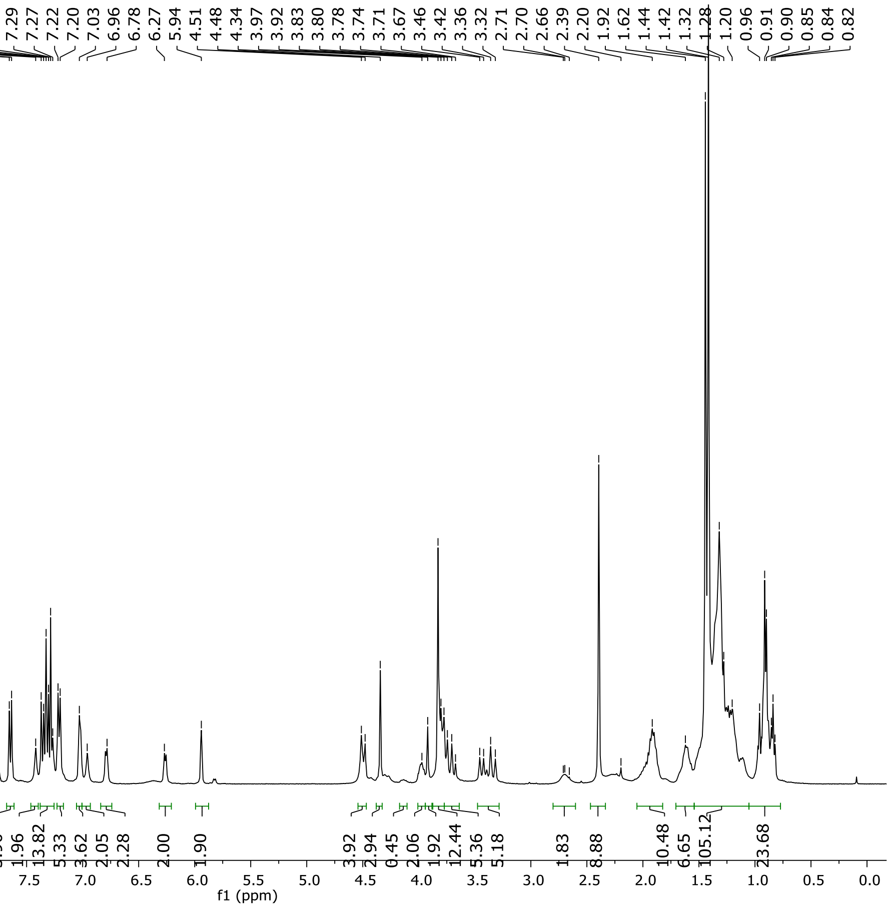


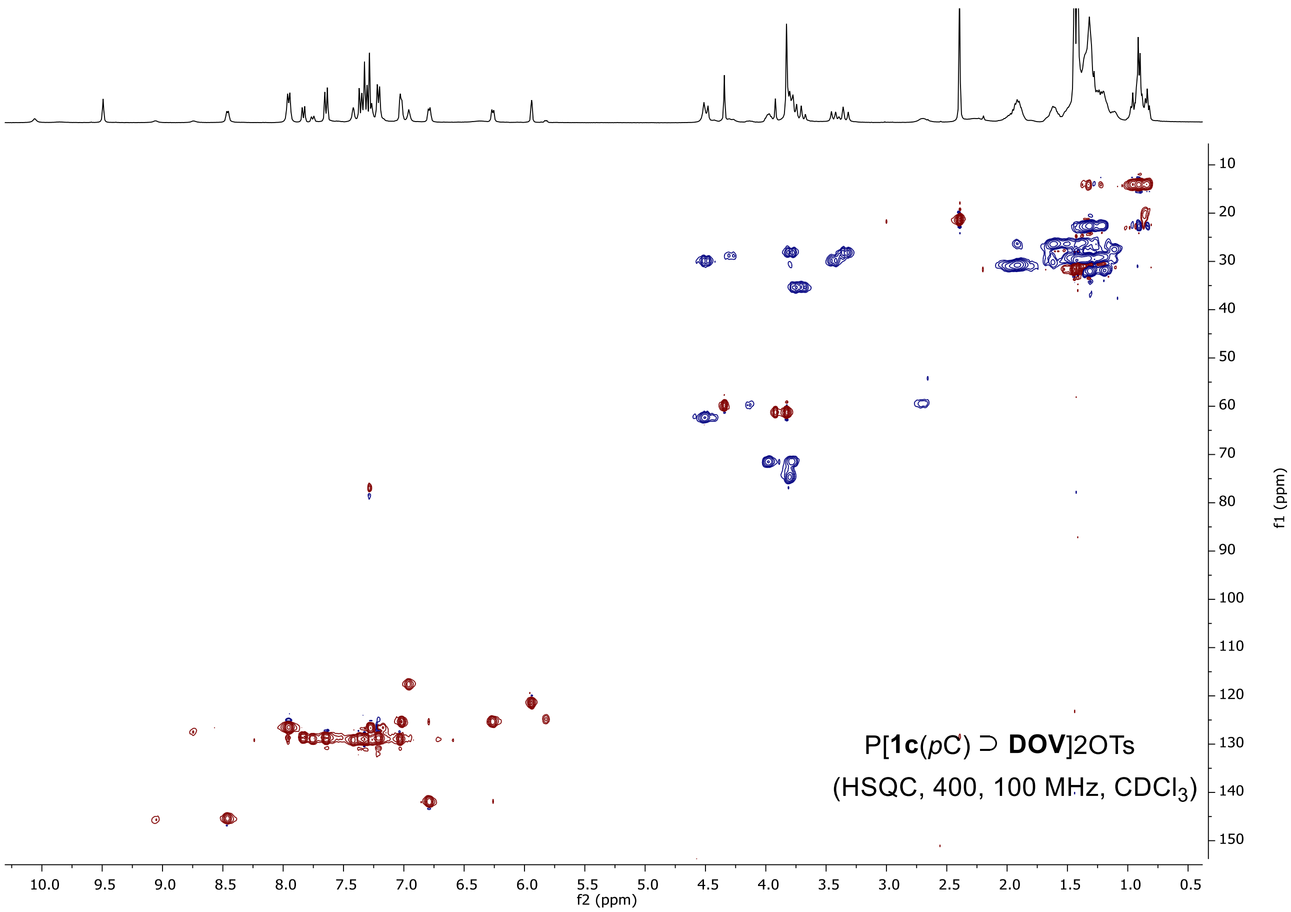


P[**1c**(*p*C) ⊃ **DOV**]2OTs
(HSQC, 400, 100 MHz, $CDCl_3$)

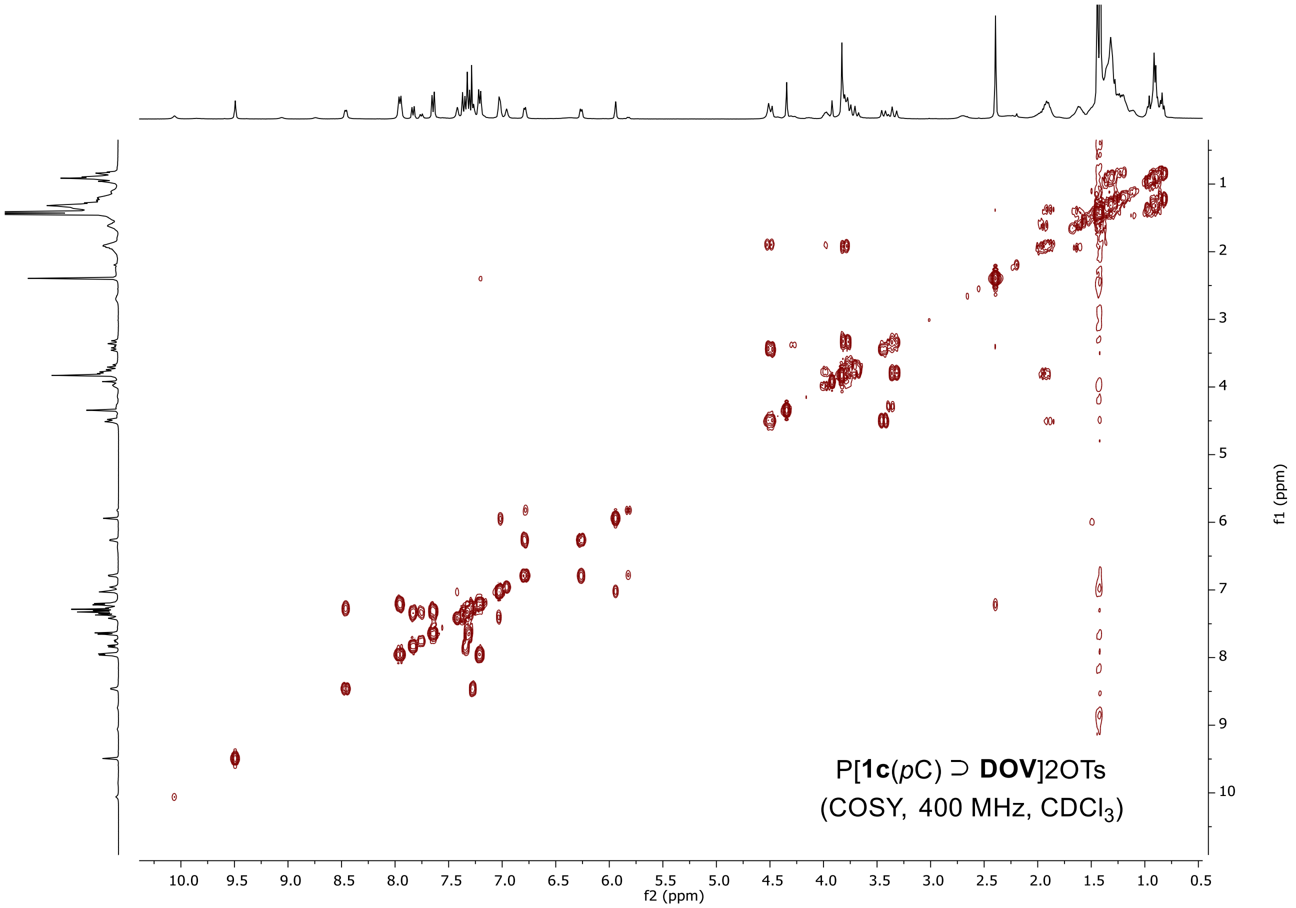
P[**1c**(*p*C) ⊃ **DOV**]2OTs
(COSY, 400 MHz, $CDCl_3$)
f1 (ppm)
f2 (ppm)

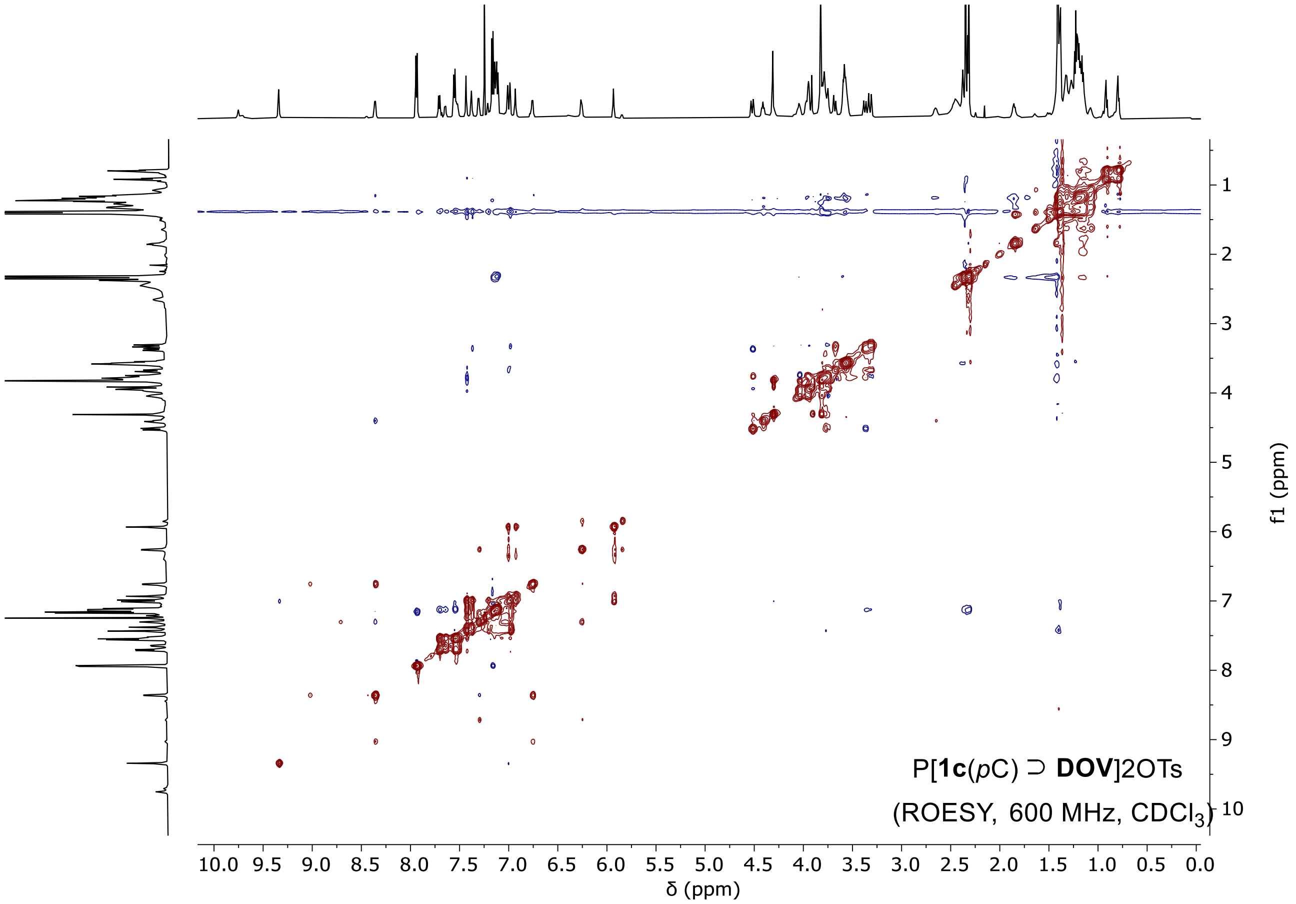
P[**1c**(*p*C) ⊃ **DOV**]2OTs
(ROESY, 600 MHz, $CDCl_3$)
f1 (ppm)
δ (ppm)

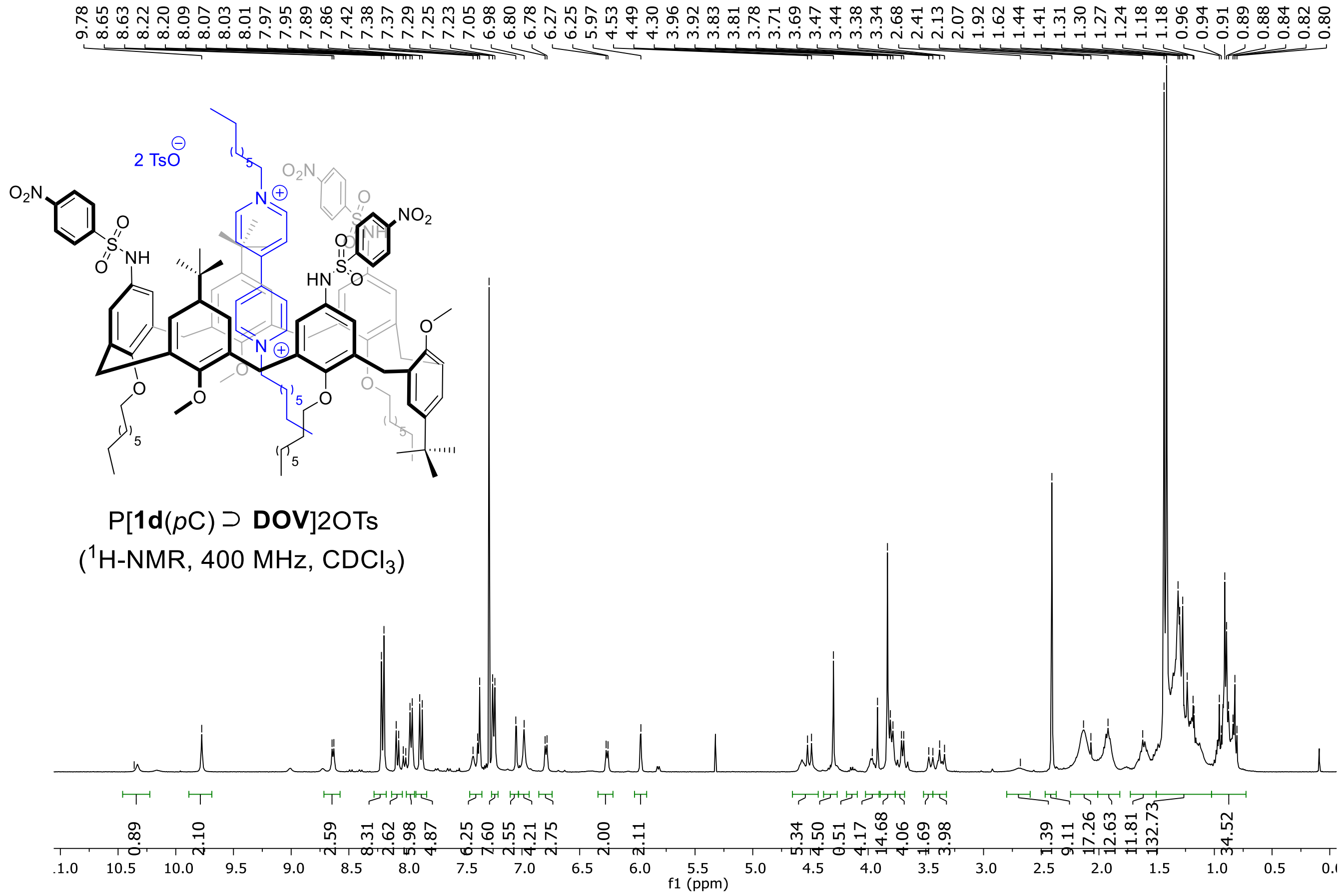
2 TsO
$O_2N$
NH
HN
$NO_2$
P[**1d**(*p*C) ⊃ **DOV**]2OTs
($^1$H-NMR, 400 MHz, $CDCl_3$)
f1 (ppm)

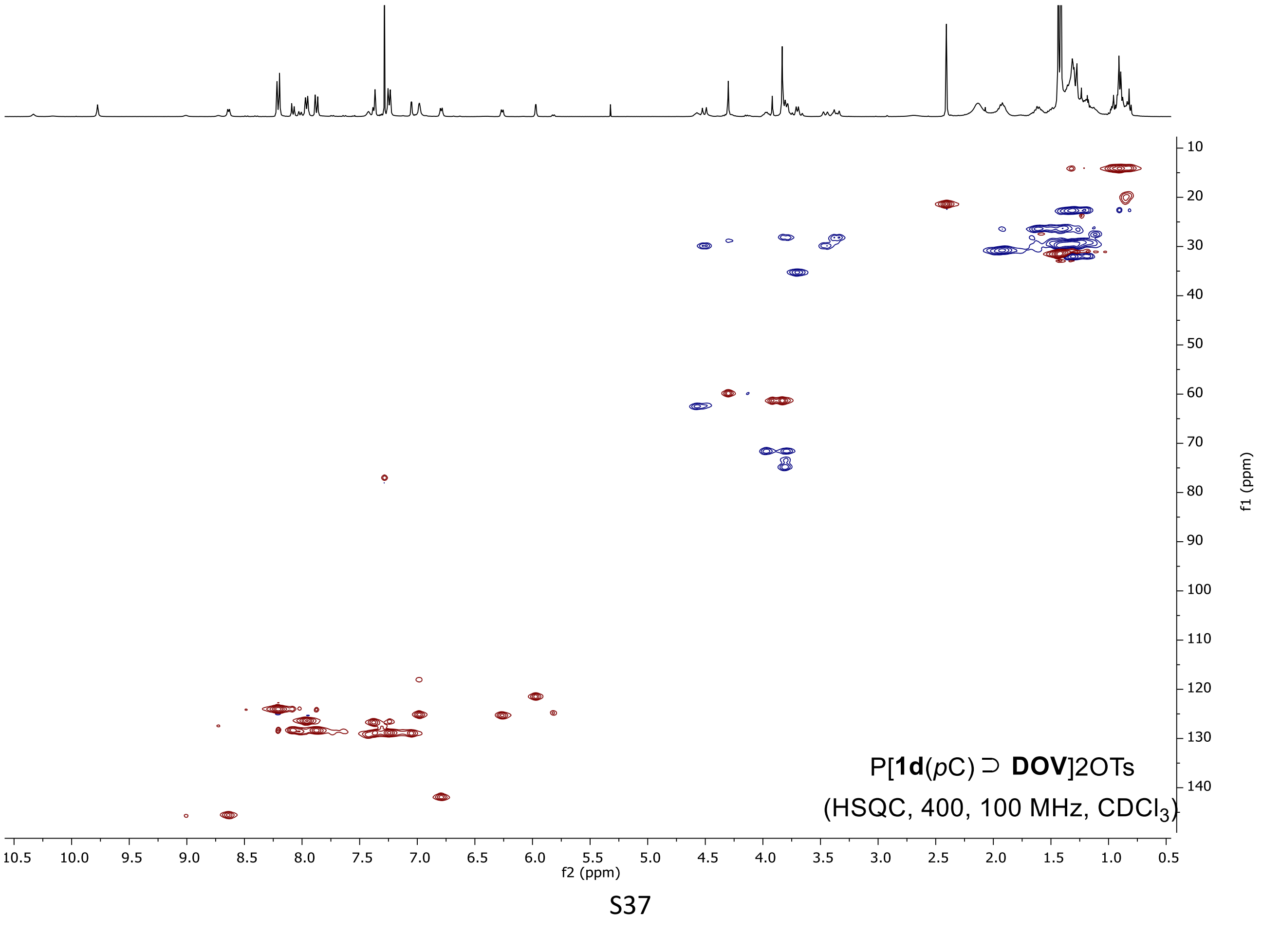
P[**1d**(*p*C) ⊃ **DOV**]2OTs
(HSQC, 400, 100 MHz, $CDCl_3$)
f2 (ppm)
f1 (ppm)

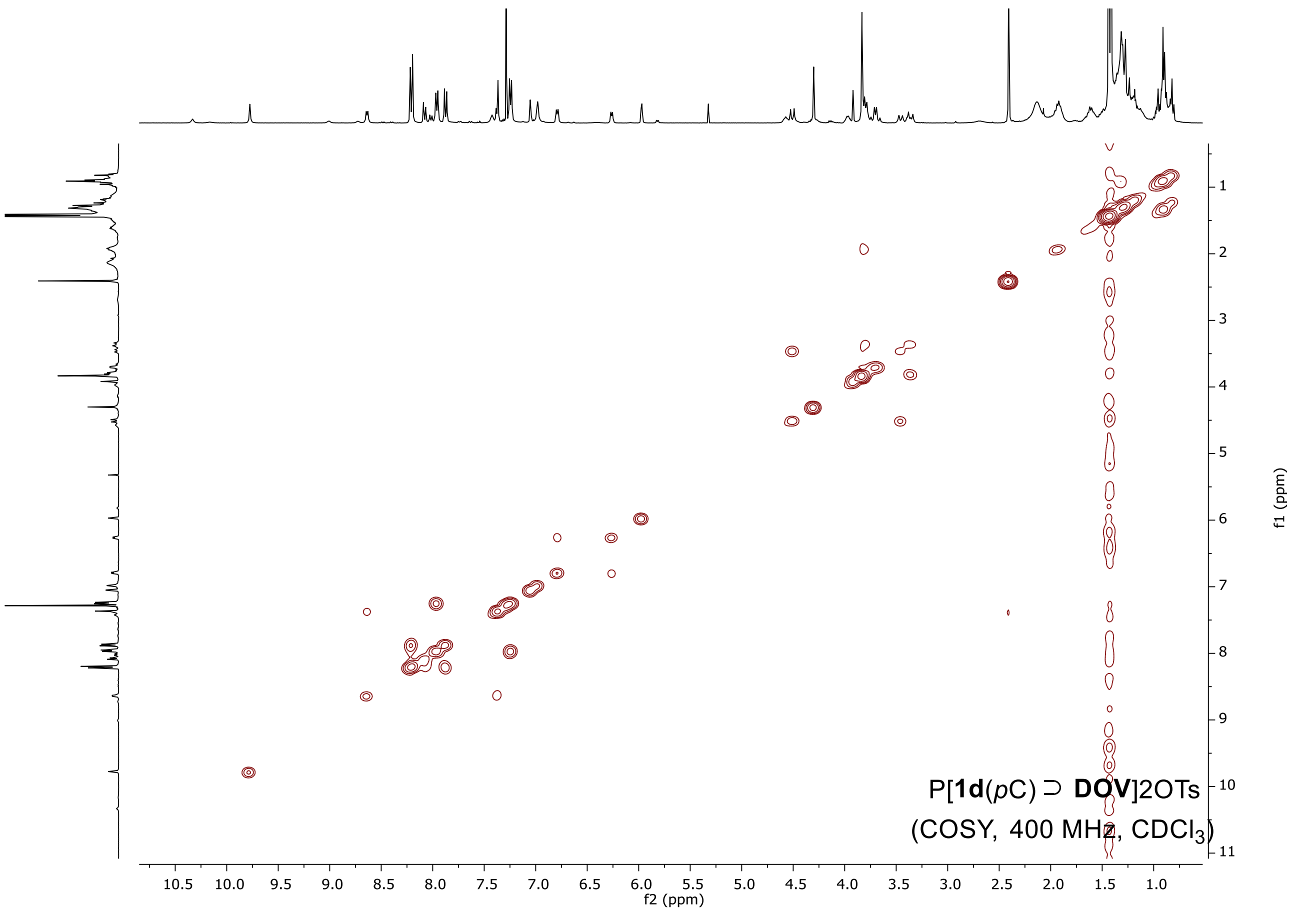
P[**1d**(*p*C) ⊃ **DOV**]2OTs
(COSY, 400 MHz, $CDCl_3$)
f2 (ppm)
f1 (ppm)

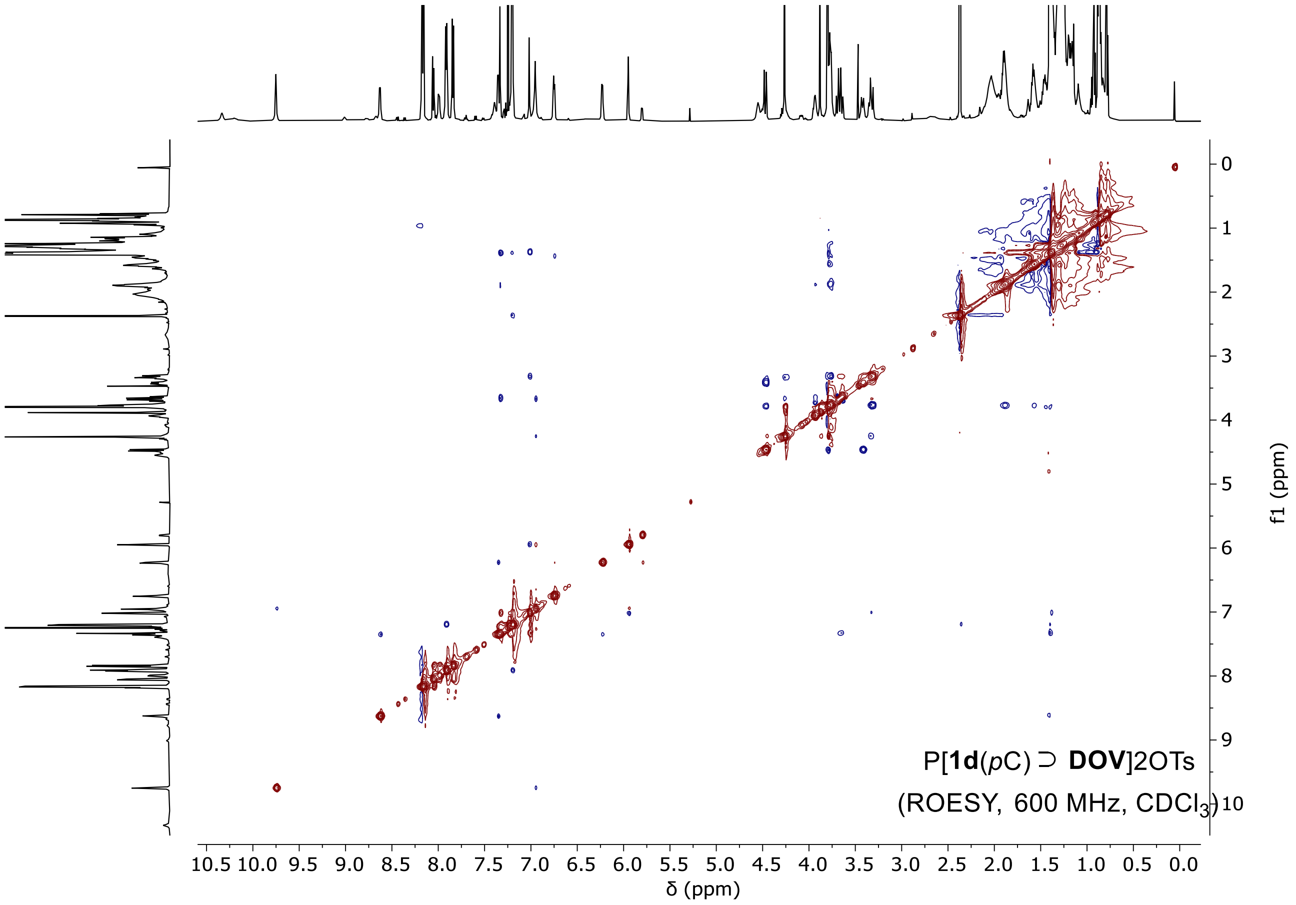
P[**1d**(*p*C) ⊃ **DOV**]2OTs
(ROESY, 600 MHz, $CDCl_3$)
δ (ppm)
f1 (ppm)

# Spectrophotometric Titrations.

[PROGRAM]
Name = SPECFIT
Version = 3.0

[FILE]
Name = TIT_GC6_MULTISCAN17.EXP
Path = C:\SPECFIT\DATA\
Date = 09-gen-20
Time = 10:18:46
Ncomp = 2
Nmeas = 20
Nwave = 181

[MODEL]
Date = 09-gen-20
Time = 10:18:47
Model = 0
Index = 3
Function = 1
Species = 3
Params = 3

| [SPECIES] | [COLORED] | [FIXED] | [SPECTRUM] |
|---|---|---|---|
| 1 0 0 | True | True | TITGC6_SINGLE_HOST.FIX |
| 0 1 0 | True | True | TIT_GC6_SINGLE_GUEST.FIX |
| 1 1 0 | True | False | |

| [SPECIES] | [FIXED] | [PARAMETER] | | [ERROR] |
|---|---|---|---|---|
| 1 0 0 | True | 0.00000E+00 | +/- | 0.00000E+00 |
| 0 1 0 | True | 0.00000E+00 | +/- | 0.00000E+00 |
| 1 1 0 | False | 4.37295E+00 | +/- | 1.05762E-01 |

[CONVERGENCE]
Iters = 3
Convergence = 1.000E-03
MarqConv = 7.014E-15
MarqPar = 0.0
SqSumY = 2.453E-01
SigmaY = 8.234E-03

[COVARIANCE]
7.603E-02

[CORRELATION]
1.000E+00

[END FILE]

P[**1a** ⊃ **DOV**]**2OTs**

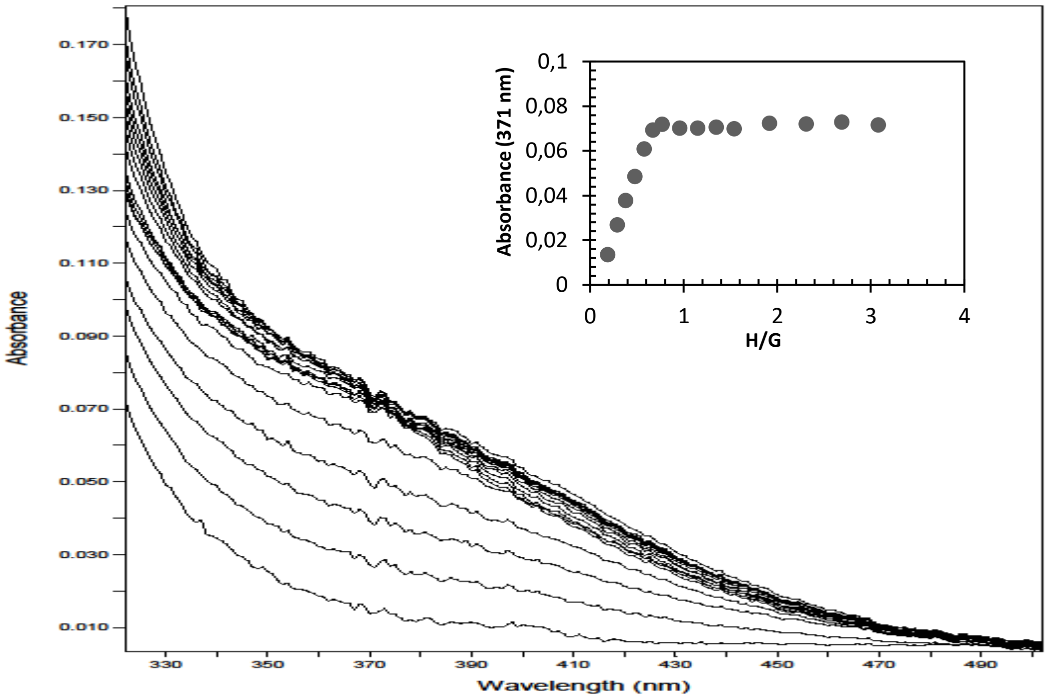

P[**1b** ⊃ **DOV**]2OTs

[PROGRAM]
Name = SPECFIT
Version = 3.0

[FILE]
Name = TITOLAZIONEMETOSSI7.EXP
Path = C:\SPECFIT\DATA\
Date = 09-gen-20
Time = 10:39:34
Ncomp = 2
Nmeas = 17
Nwave = 161

[MODEL]
Date = 09-gen-20
Time = 10:39:34
Model = 0
Index = 0
Function = 1
Species = 3
Params = 3

| [SPECIES] | [COLORED] | [FIXED] | [SPECTRUM] |
|---|---|---|---|
| 1 0 0 | True | True | BIANCORUOTAMETOSSI.FIX |
| 0 1 0 | True | True | BIANCOSALEOME.FIX |
| 1 1 0 | True | False | |

| [SPECIES] | [FIXED] | [PARAMETER] | | [ERROR] |
|---|---|---|---|---|
| 1 0 0 | True | 0.00000E+00 | +/- | 0.00000E+00 |
| 0 1 0 | True | 0.00000E+00 | +/- | 0.00000E+00 |
| 1 1 0 | False | 4.84295E+00 | +/- | 1.17137E-01 |

[CONVERGENCE]
Iters = 3
Convergence = 1.000E-03
MarqConv = 2.714E-09
MarqPar = 0.0
SqSumY = 1.718E-02
SigmaY = 2.506E-03

[COVARIANCE]
9.585E-02

[CORRELATION]
1.000E+00

[END FILE]

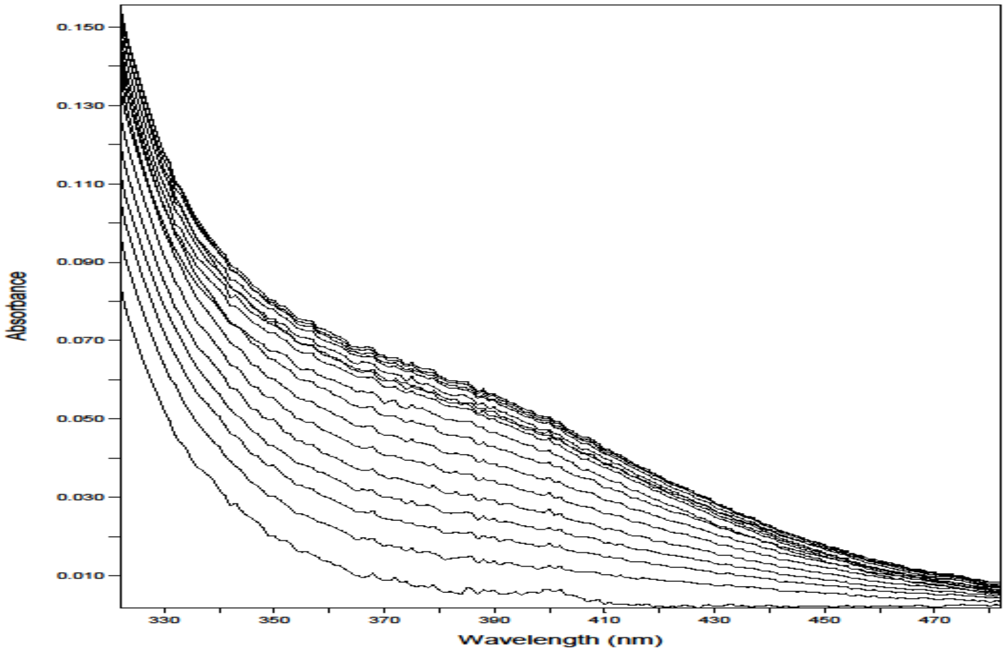

[PROGRAM]
Name = SPECFIT
Version = 3.0

[FILE]
Name = TITOLAZIONECLORO7.EXP
Path = C:\SPECFIT\DATA\
Date = 09-gen-20
Time = 10:32:36
Ncomp = 2
Nmeas = 17
Nwave = 131

**P[1c ⊃ DOV]2OTs**

[MODEL]
Date = 09-gen-20
Time = 10:32:36
Model = 0
Index = 0
Function = 1
Species = 3
Params = 3

| [SPECIES] | [COLORED] | [FIXED] | [SPECTRUM] |
|---|---|---|---|
| 1 0 0 | True | False | |
| 0 1 0 | True | True | BIANCOSALECLORO.FIX |
| 1 1 0 | True | False | |

| [SPECIES] | [FIXED] | [PARAMETER] | | [ERROR] |
|---|---|---|---|---|
| 1 0 0 | True | 0.00000E+00 | +/- | 0.00000E+00 |
| 0 1 0 | True | 0.00000E+00 | +/- | 0.00000E+00 |
| 1 1 0 | False | 4.05203E+00 | +/- | 2.81046E-01 |

[CONVERGENCE]
Iters = 5
Convergence = 1.000E-03
MarqConv = -6.889E-04
MarqPar = 0.0
SqSumY = 1.062E-02
SigmaY = 2.184E-03

[COVARIANCE]
8.282E-01

[CORRELATION]
1.000E+00

[END FILE]

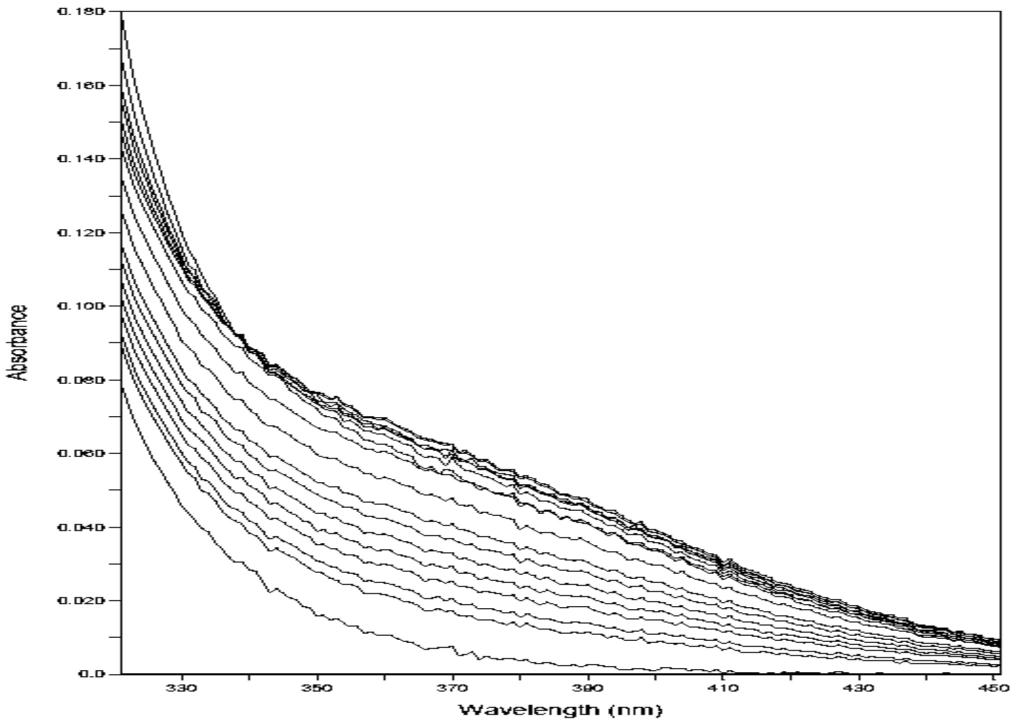

[PROGRAM]
Name = SPECFIT
Version = 3.0

[FILE]
Name = TITOLAZIONENITRO1.EXP
Path = C:\SPECFIT\DATA\
Date = 16-dic-19
Time = 17:01:17
Ncomp = 2
Nmeas = 17
Nwave = 252

P[**1d** ⊃ **DOV]2OTs**

[MODEL]
Date = 16-dic-19
Time = 17:01:17
Model = 0
Index = 0
Function = 1
Species = 3
Params = 3

| [SPECIES] | [COLORED] | [FIXED] | [SPECTRUM] |
|---|---|---|---|
| 1 0 0 | True | True | TITOLAZIONERUOTANITRO.FIX |
| 0 1 0 | True | True | TITOLAZIONESALENITRO.FIX |
| 1 1 0 | True | False | |

| [SPECIES] | [FIXED] | [PARAMETER] | | [ERROR] |
|---|---|---|---|---|
| 1 0 0 | True | 0.00000E+00 | +/- | 0.00000E+00 |
| 0 1 0 | True | 0.00000E+00 | +/- | 0.00000E+00 |
| 1 1 0 | False | 3.77853E+00 | +/- | 1.12010E-01 |

[CONVERGENCE]
Iters = 3
Convergence = 1.000E-03
MarqConv = 1.355E-15
MarqPar = 0.0
SqSumY = 1.639E+00
SigmaY = 1.956E-02

[COVARIANCE]
8.657E-02

[CORRELATION]
1.000E+00

[END FILE]

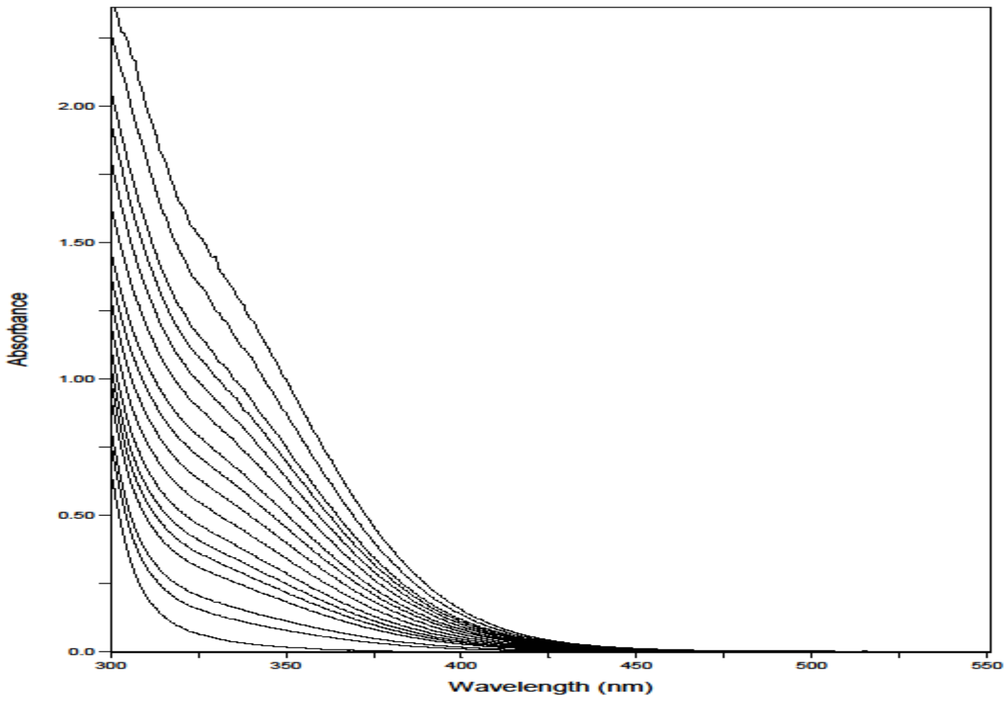

P[1a ⊃ DOV]2I

[PROGRAM]
Name = SPECFIT
Version = 3.0

[FILE]
Name = TITOLAZIONEIODURO3.EXP
Path = C:\SPECFIT\DATA\
Date = 24-gen-20
Time = 11:06:15
Ncomp = 2
Nmeas = 17
Nwave = 411

[MODEL]
Date = 24-gen-20
Time = 11:06:15
Model = 0
Index = 3
Function = 1
Species = 3
Params = 3

| [SPECIES] | [COLORED] | [FIXED] | [SPECTRUM] |
|---|---|---|---|
| 1 0 0 | True | True | BIANCORUOTATOSILE.FIX |
| 0 1 0 | True | True | SALEIODURO.FIX |
| 1 1 0 | False | False | |

| [SPECIES] | [FIXED] | [PARAMETER] | | [ERROR] |
|---|---|---|---|---|
| 1 0 0 | True | 0.00000E+00 | +/- | 0.00000E+00 |
| 0 1 0 | True | 0.00000E+00 | +/- | 0.00000E+00 |
| 1 1 0 | False | 3.29791E+00 | +/- | 8.81839E-02 |

[CONVERGENCE]
Iters = 3
Convergence = 1.000E-03
MarqConv = 1.547E-16
MarqPar = 0.0
SqSumY = 5.740E+00
SigmaY = 2.866E-02

[COVARIANCE]
5.069E-02

[CORRELATION]
1.000E+00

[END FILE]

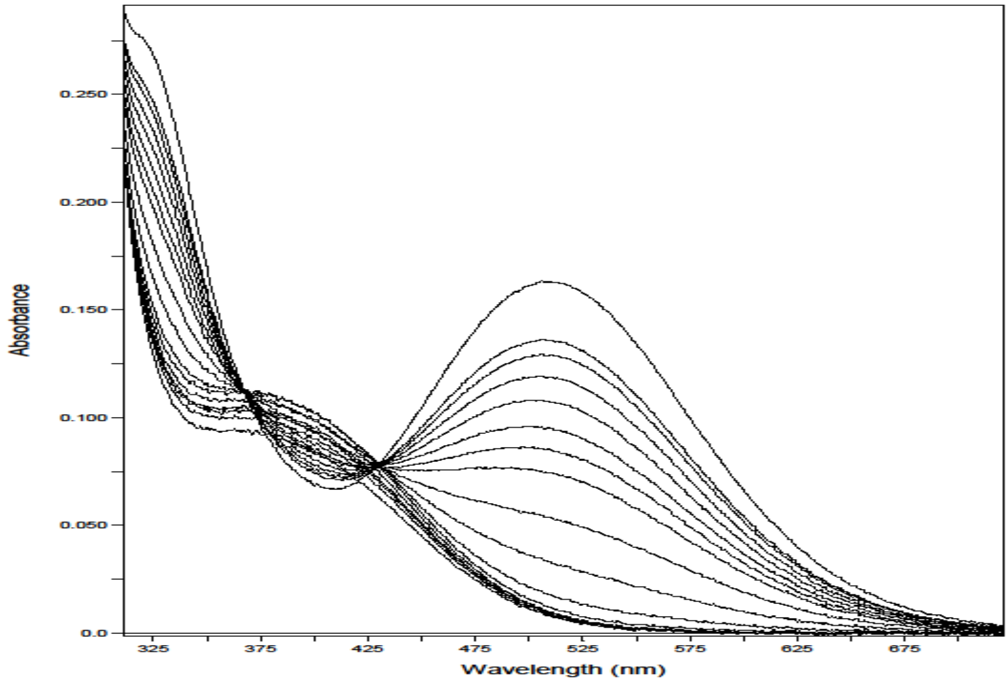

**General:** The geometry of pseudorotaxanes P[**1a**(C)⊃DOV]2TsO and P[**1a**(*p*C)⊃DOV]2TsO, and of P[**1a**(C)⊃DOV]2Cl and P[**1a**(*p*C)⊃DOV]2Cl (Fig. S15) was initially minimized with the MMFF94 force field[7] using the Avogadro software,[8] and then refined at PM6-DH+ and PM7 level[9,10] using the MOPAC2016 software.[11] The rendering of minimized structures was obtained using the Platon software.[12] The NCI analysis[13] of the minimized structures of P[**1a**(C)⊃DOV]2TsO and P[**1a**(*p*C)⊃DOV]2TsO (Fig. S17) was carried out with a script implemented in Jmol,[14] which was also used for the structures rendering.

**Figure S15.** Side and top views of the minimized structures (PM7) of pseudorotaxanes P[**1a**(C)⊃DOV]2Cl (a) and (c) ($\Delta H_f°$= -701.72889 kcal/mol) and P[**1a**(*p*C)⊃DOV]2Cl (b) and (d) ($\Delta H_f°$= -702.2226 kcal/mol). For sake of comprehension, the backbone of the dioctylviologen thread was colored in brown and the "flipped" aromatic ring of the paCo conformation in drawings (b) and (d) has been highlighted with a light blue color. All hydrogen atoms except those participating in H-bonding have been omitted. Colors: C, black; N, blue; O, red; Cl, yellow; H, white; S, green, H-bonds, dashed black lines.

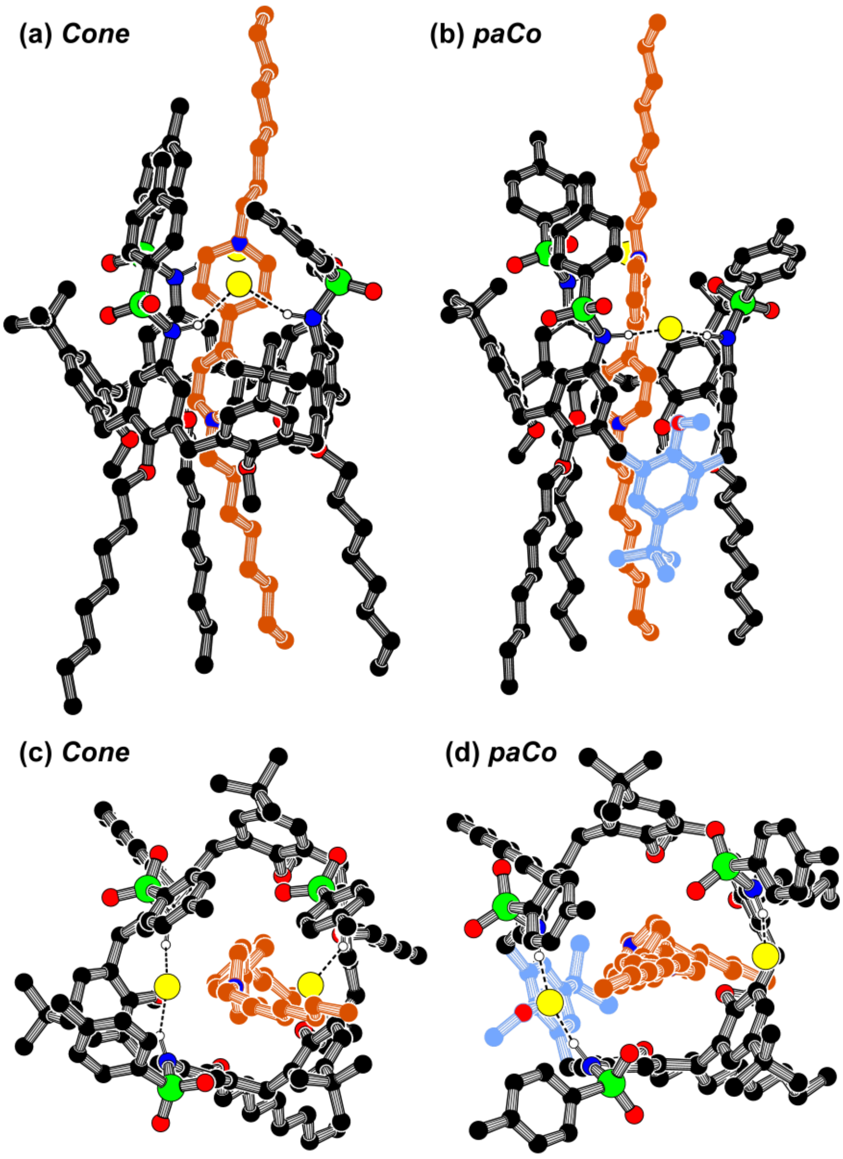

**Fig. S16.** Non-covalent interaction (NCI)-plot of pseudorotaxanes P[**1a**(C)⊃DOV]2TsO (left) and P[**1a**(*p*C)⊃DOV]2TsO (right) visualizing vdW and H-bond-like interactions as green and blue surfaces, respectively. The red surfaces represent the steric effects. To improve plot readability, all hydrogen atoms have been omitted. Colors: C, black; N, blue; O, red.

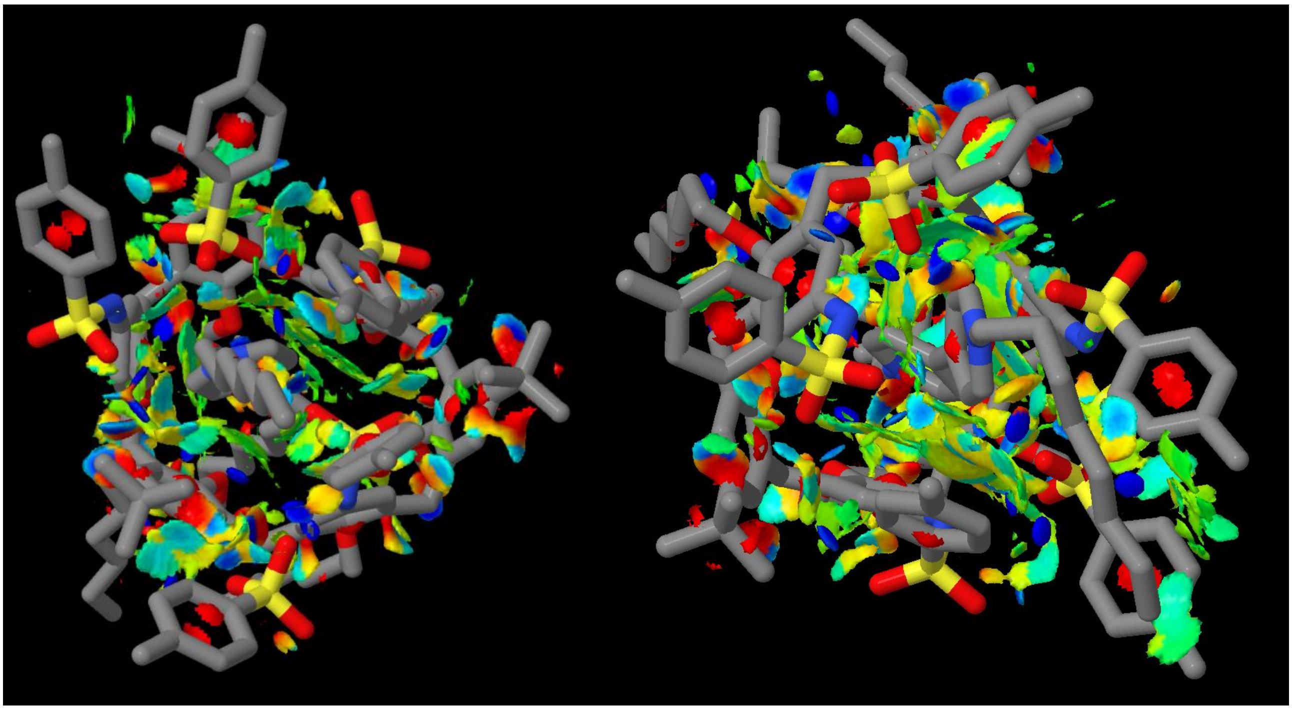

# References


(1) Gonzàlez, J.J., Ferdani, R., Albertini, E., Blasco, J. M., Arduini, A., Pochini, A., Prados, P., de Mendoza, J., *Chem. -Eur. J.*, **2000**, *6*, 73.

(2) Arduini, A., Bussolati, R., Credi, A., Secchi, A., Silvi, S., Semeraro, M., Venturi, M., *J. Am. Chem. Soc.*, **2013**, *135*, 9924.

(3) Rueping, M., Nikolaienko, P., Lebedev, Y., Adams, A., *Green Chem.*, **2017**, *19*, 2571.

(4) Ogoshi, T., Kanai, S., Fujinami, S., Yamagishi, T., Nakamoto, Y., *J. Am. Chem. Soc.*, **2008**, *130*, 5022.

(5) Vedernikov, A. I.; Ushakov, E. N.; Efremova, A. A.; Kuz'mina, L. G.; Moiseeva, A. A.; Lobova, N. A.; Churakov, A. V.; Strelenko, Y. A.; Alfimov, M. V.; Howard, J. A. K.; Gromov, S. P. *J. Org. Chem.* **2011**, *76*, 6768.

(6) Binstead, R. A. *SPECFIT/32*, Fitting Software, Spectrum Software Associates, Chapel Hill, **1996**.

(7) Halgren, T. A. *J. Comput. Chem.*, **1996**, *17*, 616-641.

(8) Hanwell, M. D.; Curtis, D. E.; Lonie, D. C.; Vandermeersch, T.; Zurek, E.; Hutchison, G. R. Avogadro: An Advanced Semantic Chemical Editor, Visualization, and Analysis Platform. *J. Cheminform.* **2012**, *4*, 17. https://doi.org/10.1186/1758-2946-4-17.

(9) Korth, M. J. *Chem. Theory Comput.*, **2010**, *6*, 3808-3816

(10) Stewart, J. J. P. *J. Mol. Mod.*, **2013**, *19*, 32.

(11) Stewart, J. J. P. *MOPAC2016*, Version: 19.206W, http://OpenMOPAC.net.

(12) Spek, A.L. *J. Appl. Cryst.* **2003**, *36*, 7-11

(13) Johnson, E. R.; Keinan, S.; Mori-Sanchez, P.; Contreras-Garcia, J.; Cohen, A. J.; Yang, W. *J. Am. Chem. Soc.* **2010**, *132*, 6498-6506.

(14) *Jmol: an open-source Java viewer for chemical structures in 3D*. http://www.jmol.org/
